# Cep57 organizes pericentriolar material through dual architectural modules and prevents human mosaic variegated aneuploidy

**DOI:** 10.1038/s41467-026-77795-4

**Published:** 2026-09-30

**Authors:** Jong Il Ahn, Jung-Eun Park, Hye-Yeoung Yun, Yan Zeng, Klara Kirsch, Vipin Kumar, Bonsu Ku, Arlene Bartolome, Harsha Ravishankar, Ruchi Bhardwaj, Rodolfo Ghirlando, Mahaiwon Shadang, Mohamadreza Fazel, David A. Ball, Jessica Matthias, Seung Jun Kim, Neerja Gupta, Jayanth Kumar Palanichamy, Tatiana S. Karpova, Kyung S. Lee

**Affiliations:** 1 Cancer Innovation Laboratory, National Cancer Institute, National Institutes of Health, Bethesda, MD USA; 2 Orphan Disease Therapeutic Target Research Center, Korea Research Institute of Bioscience and Biotechnology, Daejeon, South Korea; 3 Department of Biochemistry, All India Institute of Medical Sciences, New Delhi, India; 4 Laboratory of Molecular Biology, National Institute of Diabetes and Digestive and Kidney Diseases, National Institutes of Health, Bethesda, MD USA; 5 Laboratory of Receptor Biology and Gene Expression, Optical Microscopy Core, National Cancer Institute, National Institutes of Health, Bethesda, MD USA; 6 Abberior Instruments America, Bethesda, MD USA; 7 Critical Diseases Diagnostics Convergence Research Center, Korea Research Institute of Bioscience and Biotechnology, Daejeon, South Korea; 8 Department of Pediatrics, Division of Genetics, All India Institute of Medical Sciences, New Delhi, India

**Keywords:** Cell division, X-ray crystallography, Cytoskeletal proteins, Supramolecular assembly

## Abstract

Mosaic variegated aneuploidy (MVA) is a developmental disorder associated with chromosome segregation defects. Mutations in the centrosomal protein Cep57 cause MVA2 syndrome, yet the molecular basis of Cep57 function has remained unclear. Here, using MINFLUX nanoscopy, structural biology, biochemical approaches, and analyses of patient-derived cells, we show how Cep57 organizes the inner pericentriolar material (PCM) to support centriole biogenesis. We show that Cep57 acts as a molecular bridge between centriolar microtubules and the PCM through two architecturally distinct interactions. Its oligomeric C-terminus anchors to centriolar microtubules, whereas its dimeric N-terminus engages Cep63 through a conserved bundle-like interaction, thereby linking the Cep63–Cep152 platform to the centriole and recruiting Plk4 and other components required for procentriole formation. Analysis of primary T lymphocytes from MVA2 patients carrying the Cep63-binding-defective *CEP57*^*∆exon 3*^ mutation reveals a predominant centriole-duplication defect accompanied by minor centriole amplification, likely resulting from disorganized PCM-induced premature centriole disengagement. Our findings show Cep57 as a central architectural organizer that couples centriolar microtubules to the molecular machinery driving centriole biogenesis, providing a mechanistic framework for understanding centrosome dysfunction and aneuploidy-associated diseases.

## Abstract (web summary)

Cep57 bridges centriolar microtubules and the pericentriolar material to organize centriole biogenesis. Structural, biochemical, and patient cell analyses reveal how disruption of this architecture causes centrosome defects underlying MVA2 syndrome.

## Introduction

Mosaic Variegated Aneuploidy (MVA) is a rare genetic disorder characterized by variable gains and losses of unspecified chromosomes. It is known to be caused by mutations in the critical components of the spindle assembly checkpoint (SAC) (e.g., BubR1, Bub1, Bub3, TRIP13, Mad1, and p31Comet) or centrosome duplication (e.g., Cep57, Cep192, and CENATAC), whose dysregulation causes chromosome missegregation during cell division^1^. Patients with MVA exhibit diverse developmental disorders, such as pre- and postnatal growth retardation, microcephaly, dysmorphic facial features, and a high predisposition to cancer due to chromosome instability. Therefore, investigating MVA is vital for understanding the mechanism linking aneuploidy to disease and for developing targeted therapies that stabilize chromosome segregation.

The molecular mechanisms underlying how mutations in regulators of the SAC cause chromosome segregation errors^2^ and MVA^1^ are well understood, thanks to extensive studies of these proteins. However, the mechanisms by which mutations in *CEP57* (also known as MVA2)^3^, *CEP192*^4^, or *CCDC84* (encoding CENATAC)^5^ induce the MVA syndrome remain largely elusive. Interestingly, various studies with *CEP57* showed that either C-terminal truncating mutations (c.241 C > T, c.312 T  >  G, c.520_521delGA, c.679delA, c.915_925dup11, etc.)^6–10^ depleting its implied MT binding activity^11^ or an N-terminal exon 3 deletion (c.382+2 T > C; refer to as *∆exon 3* hereafter)^12^ missing the Cep63-binding motif (i.e., Cep57 (64–95) motif)^13,14^ causes the MVA2 syndrome with a similar degree of phenotypic severity. These findings suggest that two disparate domains act in concert to promote Cep57 function, which is crucial for maintaining genomic integrity.

At the cellular level, Cep57 is reported to be recruited to the outer region of newly formed mother centrioles during G1, forming a torus-like morphology^14,15^. Tens of other PCM proteins are found around the centriole, often exhibiting concentric, cylinder-like localizations with distinct diameters^16–19^. A long-standing question has been understanding how these proteins are organized around the centriole. A fascinating glimpse into this organization came from a study of two inner PCM scaffold proteins, Cep63 and Cep152, which cooperatively generate a higher-order cylindrical self-assembly by forming an antiparallel heterotetrameric complex^20^. This pericentriolar architecture appears central to modulating the function of polo-like kinase 4 (Plk4)^20,21^, a key regulator of centriole biogenesis that binds to the N-terminal motif of Cep152^22,23^. Mechanisms that address how activated Plk4 promotes the recruitment of various downstream components, including STIL, SAS6, and CP110, have been extensively studied^24–27^. However, how the self-assembled Cep63•Cep152 platform, pivotal for positioning Plk4, is stably anchored to the centriolar structure, and what roles Cep57 plays in this event, remain central to understanding the architectural foundation for organizing PCM.

In this study, we combine high-resolution MINFLUX nanoscopy^28^, biochemical and structural analyses, and cell-biological analyses using primary T cells from MVA2 patients to investigate how Cep57 organizes inner PCM assemblies and how a defect in this process leads to the development of MVA2 syndrome. Our results show that Cep57 is a dual structural scaffold that anchors the Cep63•Cep152-assembled platform to centriolar MTs through its two distinct, N-terminal Cep63-binding and C-terminal MT-bundling properties. Complete loss of Cep63 binding by the MVA2-linked *∆exon 3* mutation in *CEP57*, greatly destabilizes Cep63, Cep152, and other associated proteins, resulting in centriole duplication failure and precocious centriole disengagement, thereby underpinning the development of the MVA2 syndrome. In addition, disrupting the α-helix (301–316) motif, within which a MVA2-linked p.L309Pfs*9 mutation was found, deprived the capacity of Cep57 to oligomerize, stably bind to centriolar MTs, and promote centriole duplication. Thus, the dual architectural properties of Cep57—its N-terminal coiled-coil-dependent Cep63 binding and its C-terminal domain-dependent centriolar MT binding—are fundamental in organizing inner PCM assemblies that ensure proper centriole duplication while preventing precocious centriole disengagement and aneuploidy. A failure in the function of any one of the two Cep57 domains causes MVA2 syndrome.

## Results

### Nanoscopic mapping of Cep57 in the PCM space

**Fig. 1:**
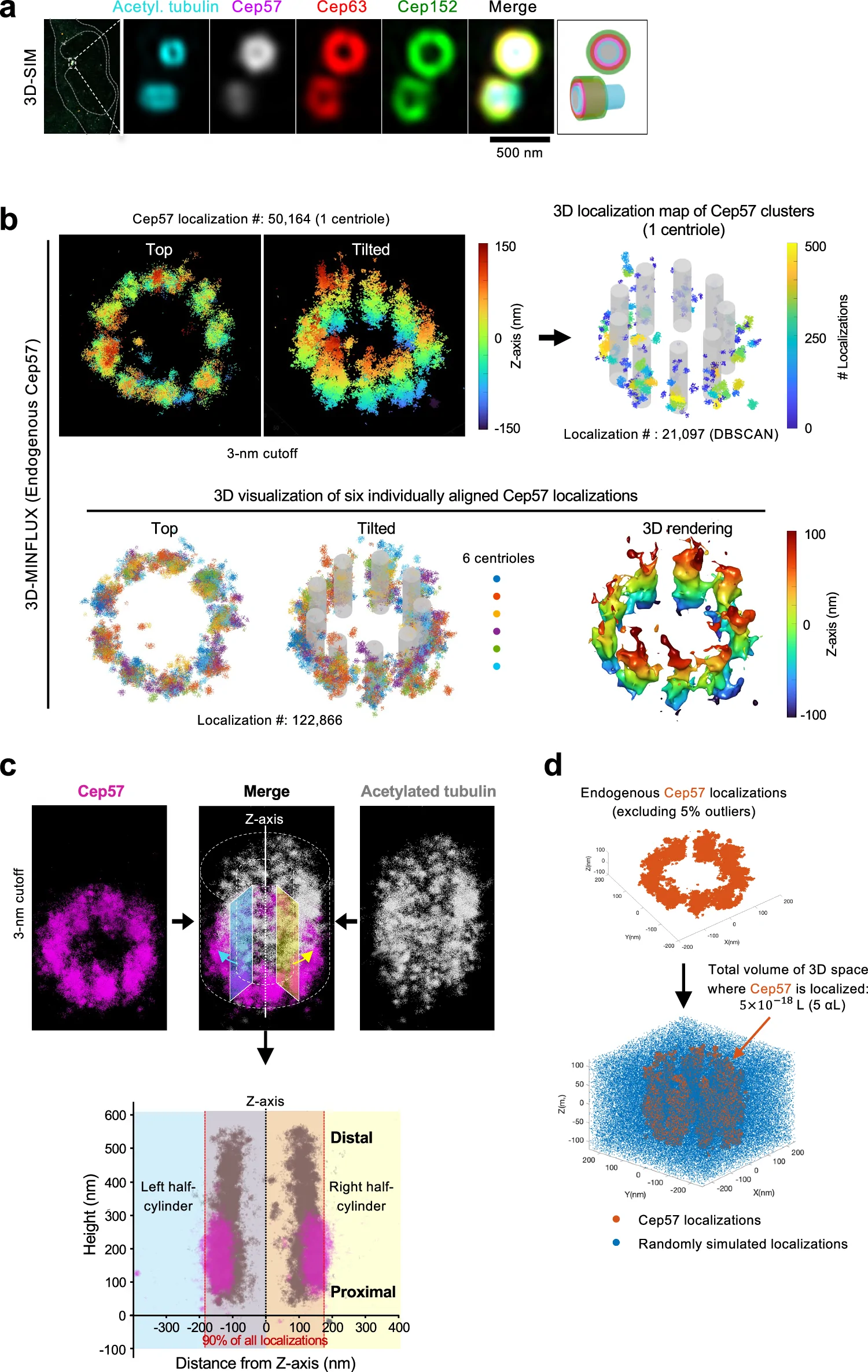
3D-MINFLUX nanoscopy visualizes the close spatial association of Cep57 and acetylated tubulin along the longitudinal axis of a centriole. **a** Representative 3D-SIM images of concentrically localized Cep57, Cep63, and Cep152 signals around the acetylated tubulin-decorated centriole in immunostained U2OS cells. A schematic diagram (right) shows the localization patterns of all four proteins. **b** 3D-MINFLUX imaging in asynchronously growing U2OS cells visualizes endogenous Cep57 localizations along the longitudinal axis of a mother centriole with nine-fold rotational symmetry. Filtered localizations (top left; 3-nm cutoff) and DBSCANed clustered localizations (top right) of Cep57 were detected along a 9-fold symmetrical template (gray) [200 nm (H) x 240 nm (D)]. Afterward, localizations from 6 processed datasets were individually aligned and fitted using an MC-randomized search^32^ and a self-developed ICP algorithm. A composite map of all Cep57 localizations around the centriole (**b**, bottom left) and its 3D surface-rendered image (**b**, bottom right) was generated by MATLAB. **c** Merge of the relative molecular density maps showing a substantial fraction of Cep57 localizations overlapping with acetylated tubulin localizations. The merged dataset (middle) was generated by aligning the proximal ends of the cylindrical Cep57 localizations (Supplementary Fig. 1c) and the acetylated tubulin localizations (Supplementary Fig. 1d) (see Methods for details). The 2D view (bottom) showing the localizations of Cep57 (magenta) and acetylated tubulin (gray) was generated by cumulatively plotting all localizations detected in the left- or right-half cylinder. **d** A schematic diagram showing how the volume occupied by Cep57 localizations was estimated. 100,000 localizations (blue dots) were simulated with a uniform, random distribution within the bounding box of the structure. The bounding box measured 400 × 400 × 200 nm for a volume of 3.2 × 10^−17 ^L. The fraction of blue spots within 6 nm of Cep57 localizations (red dots) was estimated as described in Methods, and then multiplied by the box volume to determine the volume where the Cep57 localizations were identified (See Methods for details).

Cep57 is found at the outskirt of a centriole^14,15^, showing a concentrically organized pattern with outer Cep63 and Cep152 signals at the proximal end of a centriole (Fig. 1a and Supplementary Fig. 1a)^29^. Expansion microscopy revealed 9-fold symmetrically located Cep57 signals at the outer edge of centriolar MTs^30^. Since Cep57 can associate with MTs^11^, this hints that centriolar MTs govern the pericentriolar organization of Cep57. To closely investigate the underlying mechanism of Cep57 localization in the crowded milieu of unexpanded pericentriolar space, we employed the 3-dimensional (3D) Minimal Photon FLUX (MINFLUX) nanoscopy^28,31^, which enables nanometer-scale precision mapping of individual fluorescent emitters at high spatial resolution.

To achieve localization-precision-based analysis, MINFLUX localizations of Cep57 acquired from immunostained U2OS cells were first filtered by applying an interposition distance of 3 nm (i.e., a 3-nm cutoff) (Supplementary Fig. 1b and Supplementary Movie 1). The resulting localizations were then subjected to density-based spatial clustering (DBSCAN) to identify densely clustered areas (Supplementary Fig. 1c) for further analysis (see below). The localizations were aligned and fitted using a Monte Carlo (MC) randomized search^32^ and a self-developed 3D Iterative Closest Point (ICP) algorithm (See Codes for MINFLUX data analysis attached) using a 9-fold symmetrically arranged cylindrical template (gray cylinders in Fig. 1b). The resulting six individually mapped Cep57 localizations were combined to generate a high-resolution map that reveals the relative molecular density of Cep57 in the 3D pericentriolar space (Fig. 1b, bottom, and Supplementary Fig. 1c, right). The result revealed patches of heterogeneously clustered Cep57 localizations, showing distinct 200-nm-high, elongated zones arranged in 9-fold symmetry. The varying degree of clustered Cep57 localizations may, in part, be due to the distinct physicochemical state of Cep57 or to the inherent stochasticity of fluorophore emission and detection. A second 3D-MINFLUX imaging, performed similarly to detect acetylated tubulin of the mother centriole, revealed centriolar MTs with an approximate height of 500 nm, showing clustered localizations distributed along their length (Fig. 1c, Supplementary Fig. 1d, and Supplementary Movie 2).

Due to the technical challenges of performing two-color MINFLUX imaging, the merged relative molecular density maps of Cep57 and acetylated tubulin were generated by concentrically aligning their nine axes (Supplementary Fig. 1c, d), as previously reported^30^, and leveling their proximal ends (see Methods for details). Inspection of the merged maps revealed that a substantial fraction of the mapped Cep57 and acetylated tubulin localizations are found in close proximity, showing considerable spatial coincidence (the overlapping space in Fig. 1c and Supplementary Movie 2). Since Cep57 can bind MTs directly (see below), its localization could be directed by the 9-fold-symmetrical centriolar MTs. Interestingly, when densely clustered Cep57 localizations containing 50–500 localizations within a 5-nm interposition distance (see details in Methods) were analyzed, more than half of the localizations (*n* = 12 centrioles) were found in large clusters containing at least 200 localizations (Supplementary Fig. 1e, f). The volume of these clustered localizations was proportional to the number of localizations detected (Supplementary Fig. 1f). Analysis of the localizations filtered with a relative density-based 3-nm cutoff yielded results similar to those with a 5-localization cutoff, except for one centriole out of 12 centrioles analyzed (Supplementary Fig. 1f). The substantial heterogeneity in Cep57 cluster size may suggest the complexity of its dynamic behavior in the PCM (see Fig. 3 below), although variations in labeling efficiency could also have contributed. Cep57 localizations were detected within a 5 *a*L volume of the confined proximal pericentriolar space (Fig. 1d). Given Cep57’s liquid*–*liquid phase-separating capacity^33^, weakly interacting, large-molecular-weight (MW) Cep57 complexes are also expected to form in a densely packed pericentriolar space.

### Cep57, but not its paralog Cep57L1, stably binds to tubulins and induces MT bundling in vitro

Earlier studies show that Cep57 and its paralogue Cep57L1 function redundantly to promote centriole duplication by binding to Cep63^14^ and to prevent precocious centriole disengagement^34^. Interestingly, several mutations in Cep57 are linked to MVA syndrome, whereas no MVA-causing Cep57L1 mutations have been reported to date, suggesting functional differences between the two proteins. In line with this view, overexpressed Cep57, but not Cep57L1, induces cytoplasmic MT bundling in cultured cells, and the Cep57 C-terminal domain (i.e., Cep57-C; residues 272–500) is necessary and sufficient for this event^11^ (Supplementary Fig. 2a, b). Interestingly, both Cep57 N-terminal domain (i.e., Cep57-N) and Cep57-C localize to centrosomes (Supplementary Fig. 2b), suggesting the bivalency of Cep57’s centrosomal localization.

**Fig. 2:**
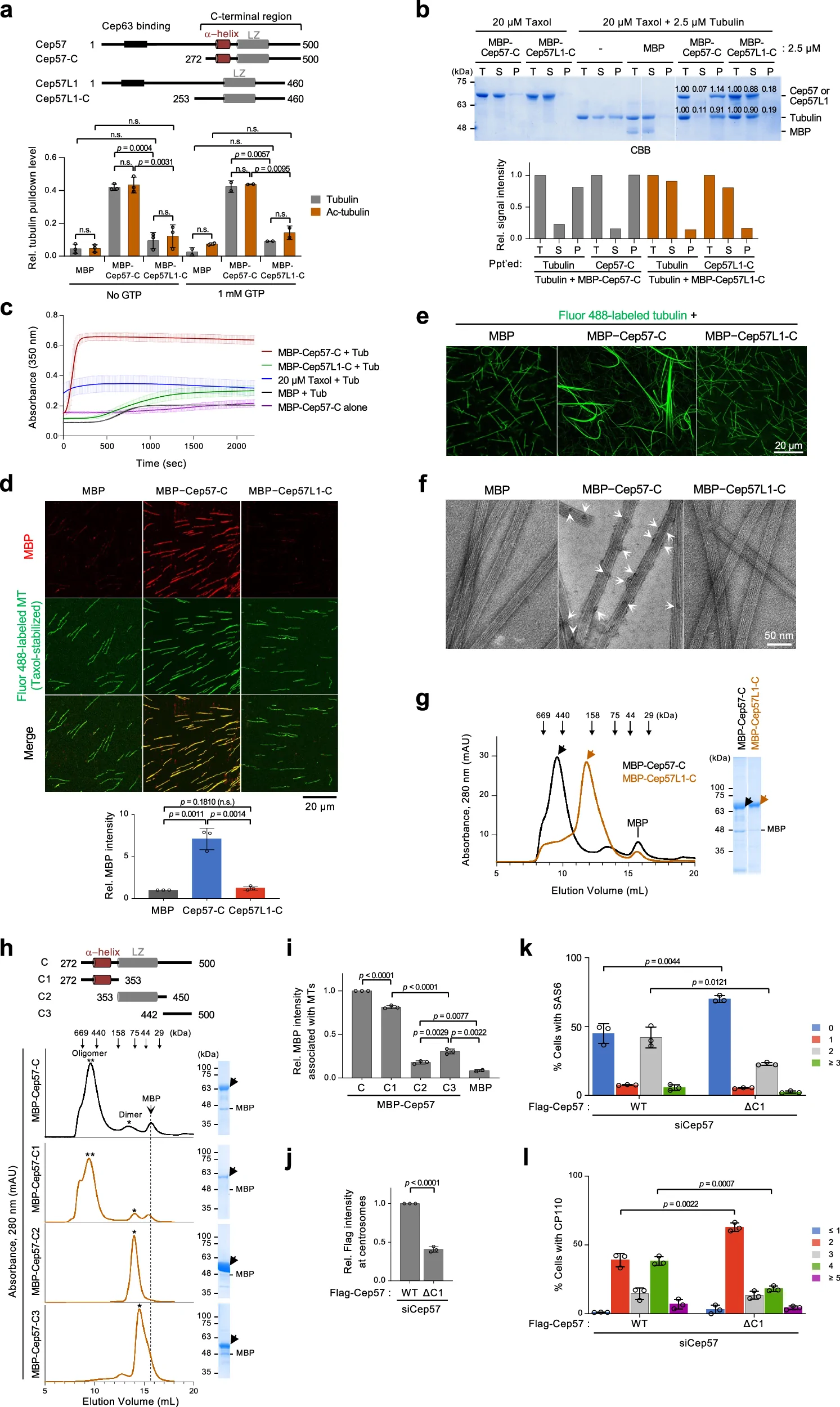
Oligomeric Cep57, but not dimeric Cep57L1, proficiently binds to tubulins/MTs, induces MT bundling, and promotes centriole duplication. **a** top, Schematic diagram showing the Cep63-binding region (thick line) in Cep57 and Cep57L1 and the distinct α-helix motif found in the C-terminal region of Cep57. Numbers, residue numbers in each protein. **a** bottom, Quantification of tubulins or acetylated tubulins co-precipitated with the indicated MBP or MBP-fused ligands from three (no GTP) or two (1 mM GTP) independent MBP pulldowns performed as described in Methods. Representative results are shown in Supplementary Fig. 2f. Bars, means ± s.d.; *P* values, unpaired two-tailed *t*-tests; ns, not significant. **b** MT co-sedimentation performed by incubating 2.5 μM of tubulin or acetylated tubulin with 2.5 μM of the indicated proteins in the presence of taxol was separated into supernatant (S) and pellet (P) fractions and analyzed by 8% sodium dodecyl sulfate–polyacrylamide gel electrophoresis (SDS-PAGE) followed by CBB staining. Numbers, relative signal intensities. The graph below shows quantified data on the fractions from the last two samples obtained from two independent experiments. Bars, means ± s.d.; *P* values, unpaired two-tailed *t*-tests. Note the MBP “S” and “P” fractions are from the same gel, cut out and flipped to place them in the correct order. **c** Tubulin polymerization assay carried out with 100 μM of tubulin mixed with 100 μM of the indicated protein or 20 μM of Taxol in a tubulin buffer (80 mM PIPES, pH 6.9, 2 mM MgCl_2,_ and 0.5 mM EGTA) supplemented with 1 mM GTP and 5% glycerol. MBP-Cep57-C alone serves as a control for its self-generated turbidity. Turbidity was measured every 10 s at 350 nm, 37 °C. Results from more than two independent experiments are shown as mean ± s.d. **d** Confocal imaging and quantification of the indicated proteins associated with Hilyte Fluor 488-labeled MTs. The proteins interacting with the MTs on a slide glass were detected by anti-MBP immunostaining (see Methods for details). Signals were quantified from three independent experiments [per experiment, *n* = 30 for MBP (total *n* = 90); *n* = 40 for MBP-Cep57-C (total *n* = 120); *n* = 40 for MBP-Cep57L1-C (total *n* = 120)]. Bars, means of three experiments ± s.d.; *P* values, unpaired two-tailed *t*tests. **e** Confocal imaging of Hilyte Fluor 488-labeled MTs polymerized in the presence of the indicated proteins, and then stabilized by the addition of 20 μM Taxol (see Methods for details). **f** Negative staining TEM images for MTs polymerized in the presence of the indicated proteins. Samples loaded onto a positively charged carbon-film-supported copper grid were stained with 1% uranyl acetate and imaged. Arrows, oligomeric Cep57-C bound to MTs. **g** SEC elution profiles of the indicated proteins analyzed with the Superdex 200 Increase 10/300 GL column. Arrows on the gel, MBP-Cep57 and MBP-Cep57L1 injected into the column. A small amount of MBP present in the sample serves as an internal control. **h** top, Schematic diagram depicting the Cep57-C (272–500) and its truncated forms (C1 to C3). The α-helix (301–316) and leucine zipper (LZ) (353–428) motifs (see Supplementary Fig. 2c–e) are marked. **h**, bottom, Elution profiles of the indicated proteins from the SEC column. The SEC profile of Cep57-C (black) is from Fig. 2g. Asterisks, putative dimeric (*) and oligomeric (**) forms eluted from the column. The position of contaminating MBP serves as an internal MW marker. Arrows on the gels, the respective proteins injected into the column. **i**, Quantification of the level of MBP-fused proteins bound to Hilyte Fluor^TM^ 488-labeled MTs performed similarly to that in Fig. 2d. Representative confocal images of anti-MBP immunostains are shown in Supplementary Fig. 2o. Relative fluorescence intensities for the indicated proteins were quantified from three independent experiments [per experiment, *n* = 50 for each Cep57-C, -C1, -C2, and -C3 (total *n* = 150)]. The control MBP signals were quantified from two independent experiments (*n* ≥ 33; total *n* = 77). Bars, means of experiments ± s.d.; *P* values, unpaired two-tailed *t*tests. **j** Quantification of centrosome-localized confocal signals using immunostained U2OS cells stably expressing the indicated constructs under Cep57 RNAi conditions (Supplementary Fig. 2p, q). Relative fluorescence intensities of Flag signals were quantified from three independent experiments [per experiment, *n* ≥ 82 for WT (total *n* = 255); *n* ≥ 85 for ∆C1 (total n = 261)]. Bars, means of three experiments ± s.d.; *P* values, unpaired two-tailed *t*-tests. **k**,**l** Quantification of centrosome-localized SAS6 (**k**) or CP110 (**l**) signals using images obtained as in (**j**). Representative images are shown in Supplementary Fig. 2r. Signals were quantified from three independent experiments [per experiment, n ≥ 83 for WT (total *n* = 279); *n* ≥ 90 for ∆C1 (total *n* = 308)]. Bars, means of three experiments ± s.d.; *P* values, unpaired two-tailed *t*tests.

Comparative sequence analyses revealed that, while Cep57 and Cep57L1 exhibit a significant level of primary sequence homology (Supplementary Fig. 2c), Cep57 bears a conserved α-helix (residues 301–316) motif (AlphaFold2-predicted) with unknown function (Fig. 2a and Supplementary Fig. 2c–e). Notably, a homozygous truncating frameshift p.L309Pfs*9 (i.e., c.915_925dup11) mutation found in patients with the MVA2 syndrome^6^ disrupts the α-helix motif due to the translational termination occurring at the Pro+9 position.

To investigate whether the α-helix (301–316) motif is critical for Cep57 function, we carried out a series of in vitro assays to compare the capacity of Cep57-C or Cep57L1-C to interact with tubulins and MTs. In maltose-binding protein (MBP) pull-down assays, Cep57-C, but not Cep57L1-C, effectively interacted with both unmodified and acetylated tubulins (Fig. 2a and Supplementary Fig. 2f) without clear binding preference between them (Supplementary Fig. 2g). In an MT cosedimentation assay performed with 2.5 μM of the protein, a concentration comparable to that of endogenous Cep57^35^, Cep57-C, but not Cep57L1-C, coprecipitated with Taxol-stabilized MTs (Fig. 2b). Cep57-C also cosedimented with acetylated MTs (Supplementary Fig. 2h), albeit at a reduced level likely because of a slower nucleation and faster shrinkage kinetics of acetylated tubulin^36^. Under these conditions, Cep57L1-C failed to detectably coprecipitate with either unmodified or acetylated MTs (Fig. 2b and Supplementary Fig. 2h). In line with the ability of Cep57-C to bind to tubulins and MTs, Cep57-C drastically enhanced MT polymerization in vitro, whereas Cep57L1-C enhanced it only weakly (Fig. 2c). Moreover, Cep57-C, but not Cep57L1-C, directly bound to in vitro-generated MTs (Fig. 2d) and stably bundled them (Fig. 2e and Supplementary Fig. 2i, j). Negative-stain transmission electron microscopy (TEM) revealed apparent protein aggregates detected along the length of polymerized MTs when incubated with Cep57-C but not Cep57L1-C (Fig. 2f and Supplementary Fig. 2k).

### The α-helix (301–316) spanning region is critical for Cep57-C’s oligomerization, MT binding, pericentriolar localization, and centriole biogenesis

To investigate the physicochemical properties of Cep57-C that confer MT bundling activity, we performed size-exclusion chromatography (SEC). Results showed that Cep57-C eluted mainly as a high-MW oligomeric form and a minor fraction as an apparent dimeric form (Fig. 2g), which were distinguishable by TEM (Supplementary Fig. 2l). In contrast, Cep57L1-C eluted mainly as a dimeric or low MW complex (Fig. 2g). In sedimentation velocity analytical ultracentrifugation analysis, Cep57-C exhibited a polydisperse distribution, with the majority sedimenting between 10 and 20 S, indicating a propensity to form higher-MW oligomers (Supplementary Fig. 2m, left). Cep57L1-C exhibited only a weak self-association activity, showing a monomer at 3.73 S (43%) and a low level of faster-sedimenting (i.e., 4.8 S–5.9 S) materials (Supplementary Fig. 2m, right). Remarkably, Cep57 (277–334), containing the conserved α-helix motif (Supplementary Fig. 2d,e), appeared sufficient to form higher-MW oligomers (Supplementary Fig. 2n). These findings suggest that Cep57-C forms an oligomeric assembly beyond the previously reported dimeric form^33,37^ and that the 277–334 region may play an important role in this event. This finding is consistent with the previous observation that Cep57 forms a condensate via liquid-liquid phase separation (LLPS) under physiological conditions^33^. Oligomerization of Cep57 could be essential for achieving high local concentrations and for promoting concentration-dependent Cep57 function in the pericentriolar space.

Unlike Cep57, no mutations in Cep57L1 are reported to cause an MVA2 phenotype. Thus, to determine whether the distinct α-helix (301–316) region found in Cep57 is required for the distinctive oligomerization capacity of Cep57 (Fig. 2g and Supplementary Fig. 2m), various MBP-fused C-terminal Cep57 fragments were generated for SEC analyses. To our surprise, the α-helix motif-containing C1 (272–353) fragment alone was able to assume a self-oligomerized form (marked **) at a level similar to that of the entire C-terminal (272–500) region. On the other hand, two other fragments (i.e., C2 and C3) eluted as a presumptive dimer (marked *) (Fig. 2h), consistent with a previous report^33^. In a related anti-MBP immunostaining analysis, C1 (272–353) efficiently bound to Taxol-stabilized MTs, although C2 and C3 also showed a low degree of MT binding (Fig. 2i and Supplementary Fig. 2o). As expected if the C1 (272–353) region is vital for Cep57 function, the Cep57 mutant lacking the C1 region (i.e., Cep57∆C1) (Supplementary Fig. 2p) failed to properly localize to centrosomes (Fig. 2j and Supplementary Fig. 2q) and recruit SAS6 and CP110 (Fig. 2k,l and Supplementary Fig. 2r), two downstream components critical for centriole duplication^24,26^.

### The oligomerization-defective V307P M314P mutant fails to properly localize to centrosomes and promote Cep57 function

**Fig. 3:**
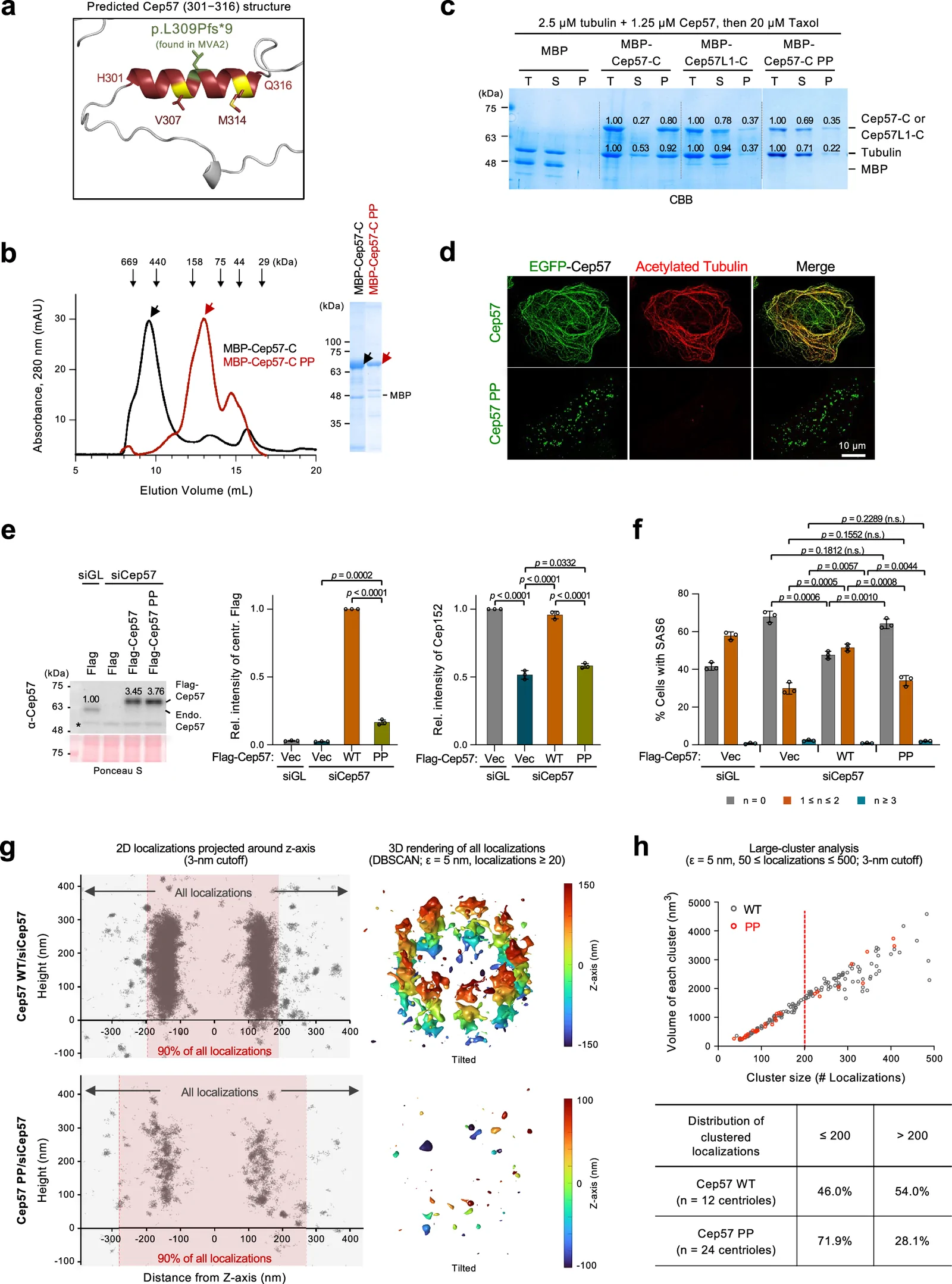
The V307P M314P mutations crippling the α-helix (301–316) motif disrupt centriole duplication by disrupting Cep57 oligomerization and pericentriolar localization. **a** A structural model predicted by AlphaFold3 showing the α-helix (301–316)-containing region. The two conserved residues (V307 and M314) mutated to Pro are denoted. **b** SEC elution profiles for MBP-Cep57-C and its respective V307P M314P (PP) mutant analyzed as in Fig. 2h. The SEC profile for Cep57-C is from Fig. 2g. **c** Microtubule co-sedimentation assay performed as in Fig. 2b. PP, the V307P M314P mutant; CBB, Coomassie Brilliant Blue-stained SDS-PAGE gel. **d** Confocal images showing U2OS cells transfected with EGFP-fused full-length Cep57 WT or its respective V307P M314P (PP) mutant. **e**,**f**, Immunoblotting (**e**, left) and immunolocalization analyses of U2OS cells stably expressing Flag-Cep57 or its respective V307P M314P (PP) mutant. Asterisk in (**e**), cross-reacting protein; Endo. Cep57, endogenous Cep57; Numbers, signal intensities. Quantification was performed with confocal images (Supplementary Fig. 3d, e) from three independent experiments [per experiment: for Flag (**e**, middle) and Cep152 (**e**, right) intensities, under siGL; *n* ≥ 50 (total *n* = 155) for vector, and under siCep57; *n* ≥ 50 (total *n* = 162) for vector, *n* ≥ 61 (total *n* = 198) for WT, *n* ≥ 65 (total *n* = 197) for PP; for SAS6 counts (**f**), under siGL; *n* ≥ 286 (total *n* = 866) for vector, and under siCep57; *n* ≥ 327 (total *n* = 1075) for vector, *n* ≥ 254 (total *n* = 809) for WT, *n* ≥ 295 (total *n* = 917) for PP]. Bars, means of three experiments ± s.d.; *P* values, unpaired two-tailed *t*tests. **g** left, The 2D views of the 3D-MINFLUX localizations for Cep57 WT or its oligomerization-defective Cep57 PP mutant were generated similarly to those shown in Fig. 1c, bottom, using 12 representative images for each obtained from mother centrioles. The areas comprising 90% of the total localizations were shaded to show how widely the PP mutant is located compared to WT. **g** right, Surface-rendered images of the clustered localizations of Cep57 WT and the PP mutant were generated using the same procedure as in Fig. 1b, bottom. **h** Analyses of the distribution pattern of large clusters (ε = 5 nm, 50 ≤ localizations ≤ 500) in (**g**) show that, unlike WT, the PP mutant is impaired in generating high-density clusters ( > 200 localizations/cluster; red-dotted line). Note that, due to the low number of PP clusters, localizations from 24 centrioles (rather than 12 for Cep57 WT) were used for the analysis.

Heptad-repeat-containing α-helices are known to form various oligomeric structures^38^. Therefore, we introduced two heptad-repeat breaking Pro (i.e., V307P M314P) (PP) mutations to the conserved α-helix (301–316) motif (Fig. 3a and Supplementary Fig. 2d,e) to examine the consequence of crippling the motif. As expected, Cep57 (277–334) exhibited alpha helicity, whereas its PP mutant did not (Supplementary Fig. 3a). Unlike the oligomeric WT form, the PP mutant appeared to be largely monomeric (Supplementary Fig. 3b,c). Consistent with these findings, the Cep57-C PP mutant, containing the entire C-terminal domain, primarily formed a monomer/dimer or low-MW complex (Fig. 3b), which co-sedimented poorly with MTs in vitro (Fig. 3c). Likewise, it also failed to bundle cytoplasmic MTs when overexpressed (Fig. 3d).

To examine the consequence of impairing the ability of Cep57 to form an oligomeric form in vivo, U2OS cells stably expressing either Cep57 or its PP mutant were analyzed after silencing endogenous Cep57 (Fig. 3e, left). Immunostaining analyses showed that the PP mutations significantly diminished Cep57’s capacity to localize to centrosomes (Fig. 3e, center, and Supplementary Fig. 3d), thereby failing to properly recruit Cep152 and downstream components, SAS6 and CP110, to centrosomes (Fig. 3e, right, 3f, and Supplementary Fig. 3e). Notably, the fraction of cells with over-duplicated centrioles (i.e., greater than three SAS6 or five CP110 signals) was not significantly increased (Fig. 3f and Supplementary Fig. 3e). This suggests that the low level of centrosome-localized Cep57 PP is sufficient to prevent precocious centriole disengagement.

To determine whether the disrupted localization of the Cep57 PP mutant to centrosomes is attributable to its inability to assume an oligomeric, high-MW form in the PCM (Fig. 3b, e), 3D-MINFLUX imaging was performed using U2OS cells stably expressing either Cep57 WT or its PP mutant under the conditions depleted of endogenous Cep57. As expected, Cep57 WT exhibited prominent, cylindrically localized signals similar to those of endogenous Cep57 in Fig. 1b (Supplementary Fig. 3f, left, and Supplementary Movie 3). In contrast, the PP mutant failed to yield a readily discernible localization pattern (Supplementary Fig. 3f, right, and Supplementary Movie 3). Comparative analyses performed with the 3D localization maps for WT (*n* = 12 centrioles) and PP (*n* = 24 centrioles) showed that, unlike Cep57 WT, which showed a 9-fold symmetrically organized pattern, the PP mutant exhibited sparse localizations scattered over a substantially wider PCM region (Fig. 3g and Supplementary Fig. 3g, h) (Note that to minimize the influence of sparsely distributed or potentially spurious localization events, the central 90% of localization signals were considered). When analyzed with densely localized clusters (ε = 5 nm, 50 ≤ localizations ≤ 500) of Cep57 WT, 53–54% of them were found in clusters of > 200 localizations (Fig. 3h and Supplementary Fig. 3i, j), a fraction similar to that of endogenous Cep57 in Supplementary Fig. 1f. Under these conditions, however, only 28–29% of the clusters generated by the oligomerization-defective Cep57 PP mutant (Fig. 3b) were > 200 localizations. Analysis of the localizations filtered with a 5-localization cutoff yielded similar results (Supplementary Fig. 3j). Thus, Cep57’s ability to form oligomeric Cep57 aggregates is a key step for localizing to the pericentriolar region and promoting centriole duplication.

### Cep57(63–120) cooperatively generates a concentration-dependent high-MW complex with Cep63(1–87)

**Fig. 4:**
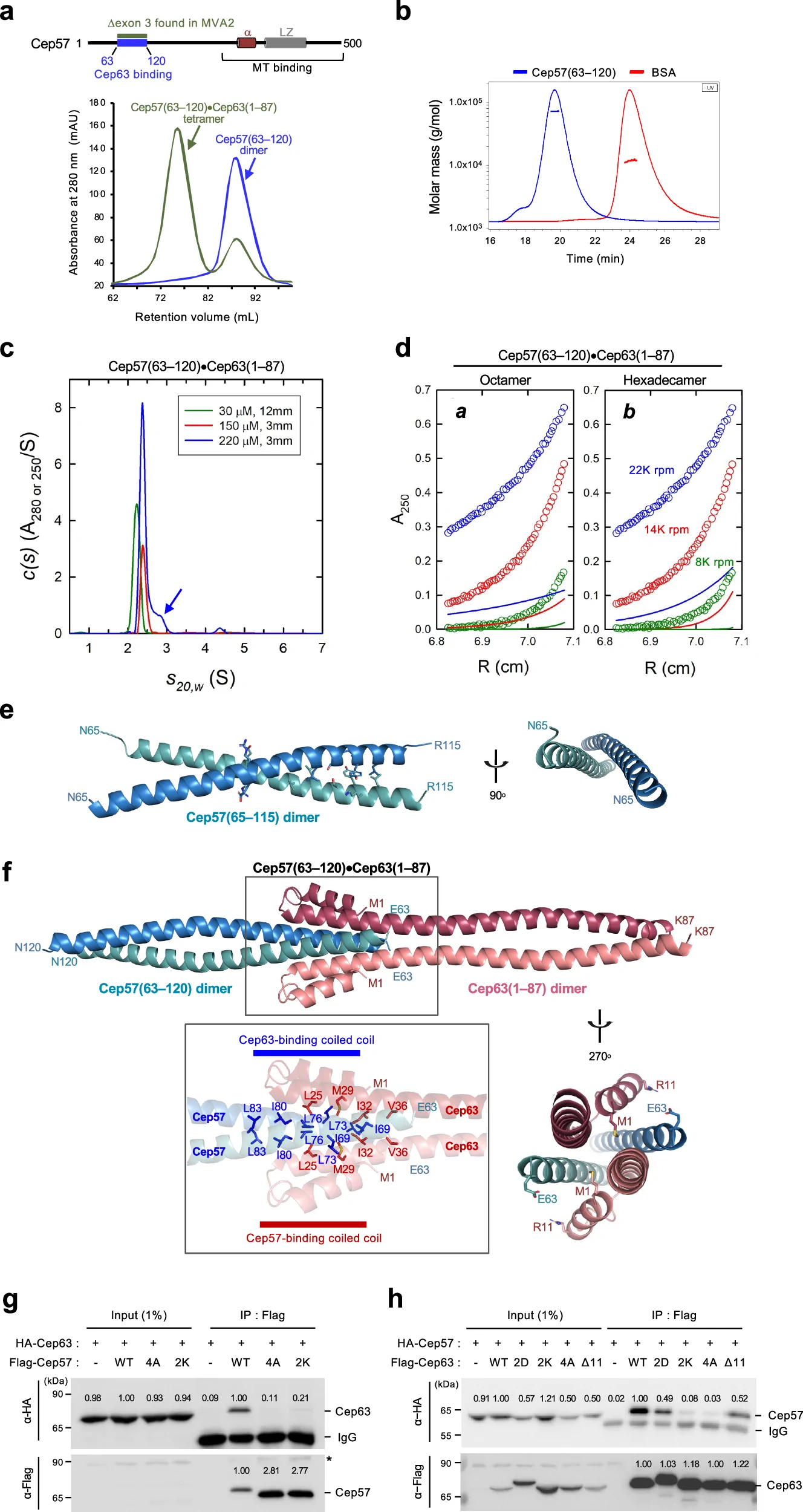
Molecular nature of the Cep57(65–115) dimeric and the heterotetrameric Cep57(63–120)•Cep63(1–87) complexes. **a** SEC profile showing the heterotetrameric Cep57(63–120)•Cep63(1–87) and dimeric Cep57(63–120) complexes eluted from the HiLoad 16/600 Superdex 200 pg column. **b** SEC-MALS analysis confirming the presence of the dimeric Cep57(63–120) complex with an estimated MW of 13.9 kDa (calculated monomer MW of 6.7 kDa). **c** Absorbance sedimentation c(*s*) profiles for the complex as a function of concentration showing evidence for the self-association of the heterotetrameric Cep57•Cep63 complex (arrow). **d** Absorbance sedimentation equilibrium data were collected with 275 µM of Cep57(63–120)•Cep63(1–87) in a 3 mm cell at 250 nm. Solid lines show the contributions of the octameric and the hexadecameric Cep57•Cep63 complexes (*a* and *b*, respectively) for each rotor speed based on the best fit shown in Supplementary Fig. 4b*(a)*. For clarity, only every third data point is displayed. Note the cooperativity in forming a hexadecameric Cep57•Cep63 complex (see details in text). **e** X-ray crystal structure of the Cep57(65–115) homodimer. The key residues for homodimerization are shown as sticks. **f** X-ray crystal structure of the Cep57(63–120)•Cep63(1–87) complex with 2:2 binding stoichiometry. The binding interface and the residues critical for forming the heterotetrameric bundle are shown (below). **g**,**h** Immunoprecipitation and immunoblotting analyses carried out with HEK293T cells co-transfected with the indicated constructs. Numbers, relative signal intensities. Asterisks, cross-reacting protein.

The homozygous Cep57 exon 3 (68–128 residues) deletion (i.e., *CEP57*^*∆exon 3/∆exon 3*^) mutation recently identified to cause the MVA2 syndrome^12^ lacks most of the Cep57 N-terminal residues 64–95 motif required for interacting with the Cep63 N-terminal region^13,14^. To investigate the nature of this interaction, SEC was performed with bacterial lysates expressing Cep57(63–120) and Cep63(1–87). Unlike a previous report^34^, Cep57 eluted as two distinct peaks consistent with the presence of a homodimer and a heterotetrameric complex with Cep63 (Fig. 4a and Supplementary Fig. 4a). SEC-multi-angle light scattering (SEC-MALS) confirmed that Cep57(63–120) forms a homodimer (Fig. 4b), in good agreement with the previous finding^33^.

In sedimentation velocity analyses performed at 30 µM, the Cep57(63–120)•Cep63(1–87) complex sedimented largely as a 2.22 S species with an estimated MW of 31 kDa (Fig. 4c), hinting that the complex is a heterotetramer with a 2:2 stoichiometry (calculated MW of 34.2 kDa). As the concentration increased, a slight shift to higher sedimentation coefficients was observed, accompanied by the appearance of a faster-migrating species (arrow), indicative of self-association.

To further characterize the physicochemical properties of the Cep57(63–120)•Cep63(1–87) complex, a series of sedimentation equilibrium analyses were performed with different concentrations of the complex and sedimentation data were obtained at 8000 (green), 14,000 (red), and 22,000 (blue) revolutions per minute (rpm) (Fig. 4d and Supplementary Fig. 4b). The weight-average molar mass increased with the loading concentration, suggesting that the complex undergoes a reversible self-association. Data analyzed globally using a tetramer–octamer–hexadecamer self-association model revealed that the binding affinity for the tetramer–tetramer interaction is much weaker (an estimated K_d_ of 1.0 mM) than the octamer–octamer interaction (an estimated K_d_ of 35 μM). This observation suggests a cooperativity in the concentration-dependent formation of a hexadecameric Cep57•Cep63 complex.

### The Cep57(63–120)•Cep63(1–87) complex forms a stable, antiparallelly arranged, 2:2 heterotetrameric structure

Inspection of the primary sequence of Cep57(63–120) and Cep63(1–87) regions revealed that each contains a predicted α-helix with heptad repeats (Supplementary Fig. 4c,d). Determination of X-ray crystal structures revealed that, while the second half of Cep57(63–120) (i.e., from E86 to L108) functions as a homodimeric domain by engaging in pairwise interactions for several residues found along the length of the α-helical structure (Fig. 4e and Supplementary Fig. 4e, f; Data collection and refinement statistics in Supplementary Table 1), the two heptad repeat motifs from each protein (Supplementary Fig. 4c, d) form an antiparallelly arranged dense hydrophobic core through bilaterally interlinked interactions (Fig. 4f and Supplementary Fig. 4g; Data collection and refinement statistics in Supplementary Table 2). The N-terminal 11 residues from Cep63 did not appear to interact significantly with the 2:2 tetrahelical Cep57(63–120)•Cep63(1–87) bundle. Results obtained with the SEC-MALS analyses performed with decreasing concentrations of the Cep57(63–120)•Cep63(1–87) complex showed that the complex remained stable even at 1 μM (Supplementary Fig. 4h), a value consistent with the previous report of the K_d_ of 0.6 μM obtained from an isothermal titration calorimetry^33^.

To corroborate the Cep57(63–120)•Cep63(1–87) structural model in Fig. 4f, we performed coimmunoprecipitation analysis with transfected lysates using mutants designed to cripple the interactions between Cep57 and Cep63. To this end, the four residues from each protein involved in forming the hydrophobic cores (I69, L73, L76, and I80 for Cep57 and L25, L28, M29, and I32 for Cep63) (Fig. 4f, bottom, and Supplementary Fig. 4g, bottom) were mutated to Ala, generating their respective 4 A mutants. Similarly, the L73 and L76 residues for Cep57 and the L28 and I32 residues for Cep63 were mutated to Lys, yielding their respective 2 K mutants. To determine whether the Cep63’s N-terminal 1–11 residues contribute to the Cep57–Cep63 interaction, a 2D mutant replacing the M1 and L5 residues to Asp and an ∆11 mutant lacking the 1–11 residues were also generated. Results showed that, while 4 A and 2 K mutations in the hydrophobic core of the Cep57•Cep63 bundle greatly impaired the Cep57–Cep63 interaction, either the 2D or the ∆11 designed to disrupt the interaction between the N-terminal Cep63 motif and the Cep57•Cep63 heterotetramer only weakly influenced the interaction (Fig. 4g, h). Interestingly, while the Cep57(63–120)•Cep63(12–87) complex lacking the 1–11 residues sedimented largely as a 2.30 S species, it also formed concentration-dependent, undefinable, large aggregates of 4 S–6 S (Note that the octamer is expected to sediment at 3.6 S) (Supplementary Fig. 4i). This suggests that the Cep57•Cep63 complex functions primarily as a heterotetrameric bundle, and the N-terminal 1–11 region of Cep63 may play an auxiliary role in properly modulating the thermodynamic equilibrium of the interaction and, therefore, the stepwise formation of higher MW complexes.

### The Cep57–Cep63 interaction mutants fail to enhance each other’s centrosomal localization and to promote centriole duplication

**Fig. 5:**
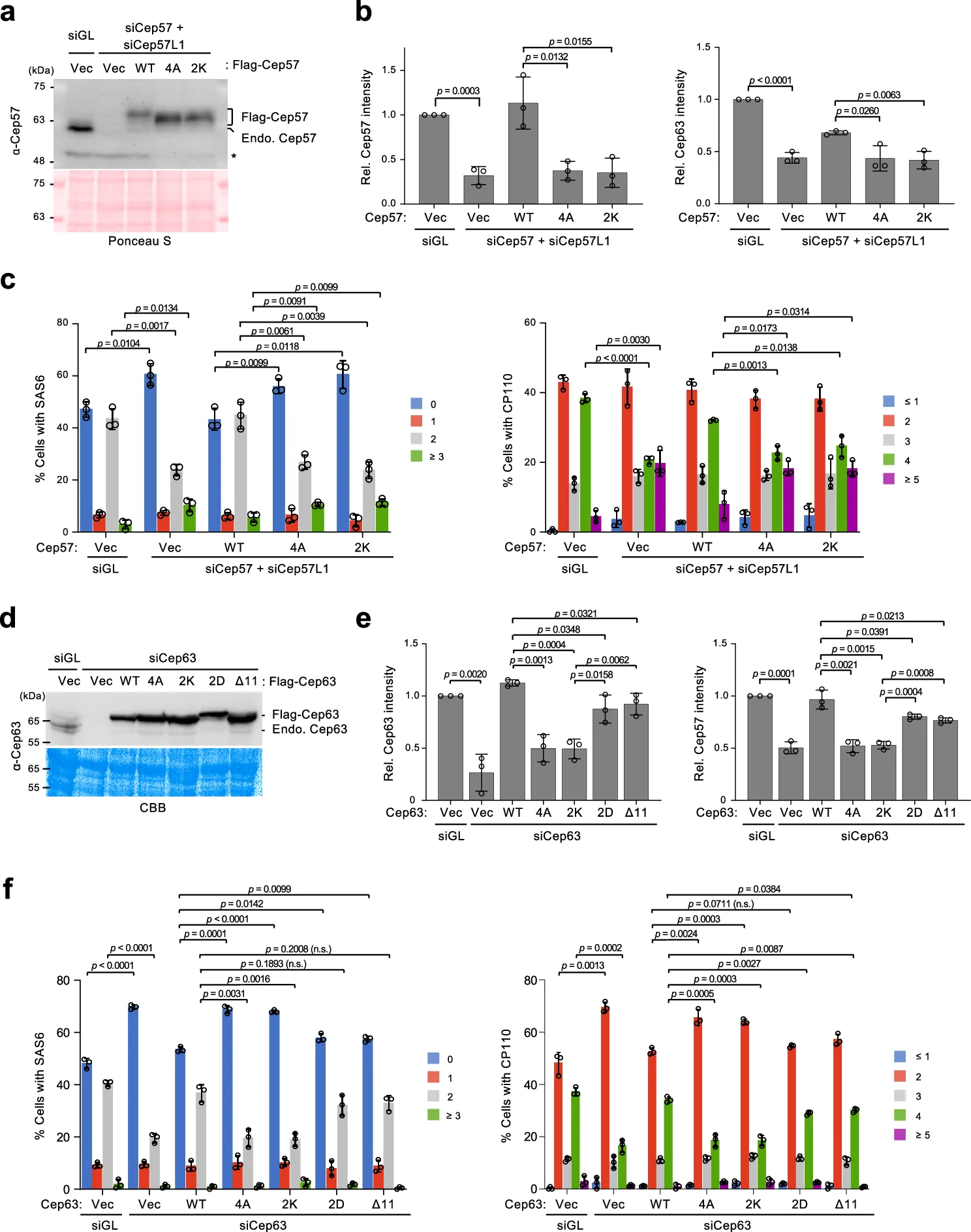
Mutations impairing the Cep57–Cep63 interaction cause defects in proper Cep57 and Cep63 localization and centriole duplication. **a**–**c** Immunoblotting (**a**) and immunolocalization (**b**,**c**) analyses of U2OS cells stably expressing the indicated Flag-Cep57 constructs under the Cep57 and Cep57L1 RNAi conditions. Asterisk in (**a**): cross-reacting protein, Endo. Cep57, endogenous Cep57. Indicated centrosome-associated signals were quantified from three independent experiments [per experiment: for Cep57 intensities (**b**, left), under siGL; *n* ≥ 154 (total *n* = 486) for vector, and under siCep57+Cep57L1; *n* ≥ 153 (total *n* = 548) for vector, *n* ≥ 150 (total *n* = 487) for WT, *n* ≥ 154 (total *n* = 534) for 4 A, *n* ≥ 149 (total *n* = 534) for 2 K; for Cep63 intensities (**b**, right), under siGL; *n* ≥ 143 (total *n* = 504) for vector, and under siCep57+Cep57L1; n ≥ 161 (total *n* = 497) for vector, *n* ≥ 145 (total *n* = 498) for WT, *n* ≥ 138 (total *n* = 472) for 4 A, n ≥ 138 (total *n* = 479) for 2 K; for SAS6 and CP110 signals (**c**), under siGL; n ≥ 102 (total *n* = 333) for vector, and under siCep57+Cep57L1; *n* ≥ 108 (total *n* = 330) for vector, *n* ≥ 105 (total *n* = 321) for WT, *n* ≥ 105 (total *n* = 324) for 4 A, n ≥ 112 (total n = 341) for 2 K]. Bars, means of three experiments ± s.d.; *P* values, unpaired two-tailed *t*-tests. Representative images used for quantification are provided in Supplementary Fig. 6a–c. **d**–**f** Immunoblotting (**d**) and immunolocalization (**e**,**f**) analyses of U2OS cells stably expressing the indicated Flag-Cep63 constructs under the Cep63 RNAi conditions. Endo. Cep63, endogenous Cep63. CBB, Coomassie-Brilliant Blue staining. Indicated centrosome-associated signals were quantified as in (**a**–**c**) from three independent [per experiment: for Cep63 intensities (**e**, left), under siGL; *n* ≥ 133 (total *n* = 433) for vector, and under siCep57+Cep57L1; *n* ≥ 146 (total *n* = 464) for vector, *n* ≥ 134 (total *n* = 427) for WT, *n* ≥ 138 (total *n* = 438) for 4 A, *n* ≥ 138 (total *n* = 457) for 2 K, *n* ≥ 138 (total *n* = 437) for 2D, *n* ≥ 134 (total *n* = 448) for ∆11; for Cep57 intensities (**e**, right), under siGL; *n* ≥ 55 (total *n* = 237) for vector, and under siCep57+Cep57L1; *n* ≥ 55 (total *n* = 240) for vector, *n* ≥ 71 (total *n* = 246) for WT, *n* ≥ 65 (total *n* = 245) for 4 A, *n* ≥ 66 (total *n* = 234) for 2 K, *n* ≥ 58 (total *n* = 227) for 2D, *n* ≥ 73 (total *n* = 261) for ∆11; for SAS6 and CP110 signals (**f**), under siGL; *n* ≥ 201 (total *n* = 625) for vector, and under siCep57+Cep57L1; *n* ≥ 159 (total *n* = 530) for vector, n ≥ 181 (total *n* = 608) for WT, *n* ≥ 168 (total *n* = 564) for 4 A, *n* ≥ 152 (total *n* = 530) for 2 K, *n* ≥ 174 (total *n* = 589) for 2D, *n* ≥ 188 (total *n* = 603) for ∆11]. Bars, means of three experiments ± s.d.; *P* values, unpaired two-tailed *t*-tests. Representative images used for quantification are provided in Supplementary Fig. 6d–f.

We then examined whether the Cep57–Cep63 interaction is vital for Cep57 function in U2OS cells by stably expressing either WT or its mutants and silencing endogenous Cep57 by RNAi. To eliminate any potential compensatory activity from Cep57L1, the endogenous Cep57L1 was also silenced (Fig. 5a–c). Under the conditions where Cep57 WT, 4 A, and 2 K were comparably expressed (Fig. 5a), both 4 A and 2 K mutants disrupting the Cep57–Cep63 interaction (Fig. 4g) exhibited a greatly diminished level of centrosome-localized signals (Fig. 5b, left, and Supplementary Fig. 5a) along with significantly compromised Cep63 intensities (Fig. 5b, right, and Supplementary Fig. 5a). Consistent with these findings, both Cep57 4 A and 2 K mutations greatly impaired normal duplication of centrosome-localized SAS6 and CP110 signals (Fig. 5c and Supplementary Fig. 5b). In addition, these cells showed a significant increase in the population with ≥ 3 SAS6 and ≥ 5 CP110 signals (Fig. 5c and Supplementary Fig. 5c), which is reported to be due to precocious centriole disengagement under Cep57 and Cep57L1 RNAi conditions^34^. Thus, disruption of the Cep57–Cep63 interaction not only impairs normal centriole duplication but also  paradoxically leads to centriole overduplication.

Next, as above, we examined the capacity of Cep63 WT or its Cep57-binding-defective mutants to promote centriole duplication under Cep63 RNAi conditions (Fig. 5d). The results showed that Cep63 4 A and 2 K mutants attenuated in the Cep57–Cep63 interaction (Fig. 4h) were defective not only in their own but also in Cep57 localization to centrosomes (Fig. 5e and Supplementary Fig. 5d, e), and failed to properly recruit SAS6 and CP110 to these sites (Fig. 5f and Supplementary Fig. 5f). The 2D and ∆11 mutants, which showed a moderately weakened Cep57 interaction, exhibited only a low level of defect in centrosome localization and in SAS6 and CP110 recruitment (Fig. 5e, f, and Supplementary Fig. 5d–f). These data, along with the results in Fig. 5a–c, suggest that the Cep57–Cep63 interaction is reciprocally required for their centrosomal localization and for promoting centriole duplication. Remarkably, unlike under the Cep57 and Cep57L1 RNAi conditions in Fig. 5c, precocious centriole disengagement was not apparent under the Cep63 RNAi conditions (Fig. 5f).

### The MVA2 syndrome-linked *CEP57*^*∆exon 3 (68–128)*^ mutation destabilizes Cep63, Cep152, and various components required for Plk4-dependent centriole duplication

Mouse embryonic fibroblast (MEF) cells bearing a homozygous p.L309Pfs*9 mutation on Cep57 cause precocious centriole disengagement and centriole overduplication with only a low level of centriole duplication defect^3^. Similarly, lymphoblastoid cell line (LCL) cells derived from Epstein-Barr virus-infected B-lymphocytes carrying compound heterozygous Cep57 p.E174Tfs*4 and p.L309Pfs*9 show centriole overduplication with precociously disengaged centrioles^15^. On the other hand, the disruption of the Cep57–Cep63 interaction, which requires a large portion of the Cep57 exon 3 region (residues 68–128) (Fig. 4f and Supplementary Fig. 4g), induced a predominant centriole duplication defect (Fig. 5). Hence, to investigate in depth the consequence of the *CEP57*^*∆exon 3*^ mutation in its natural cellular environment, we purified primary T cells from peripheral blood mononuclear cells prepared from the first-degree relatives (i.e., son, daughter, and father) carrying the *CEP57*^*∆exon 3*^ mutation in a homozygous or heterozygous state. Remarkably, immunoblotting analysis with total lysates revealed that the *∆exon 3* mutation greatly diminished the levels of not only Cep57 but also its interacting Cep63 and Cep152, and other scaffold proteins, such as PCNT and Cep192 (Supplementary Fig. 6a). Since the exon 3 region is critical for the Cep57–Cep63 interaction (Fig. 4f and Supplementary Fig. 4g), the loss of this interaction could have destabilized Cep57 and Cep63, as well as other structurally associated components critical for the Plk4-dependent centriole duplication pathway.

To verify the importance of the Cep57–Cep63–Cep152 scaffold^13,14^ in stabilizing components critical for centriole duplication, detailed immunoblotting analyses were performed after depleting Cep57, Cep63, or Cep152 in U2OS cells by RNAi. Strikingly, depletion of any one of the three proteins greatly reduced the levels of the other two and several other components (e.g., Cep192, STIL, SAS6, CPAP, and PCNT) central to the Plk4-dependent centriole duplication pathway (Supplementary Fig. 6b). CP110 and α- and γ-tubulins were less affected. These findings suggest that Cep57–Cep63–Cep152 functions as an interlinked structural platform required not only to recruit Plk4 and its downstream components but also to safeguard them from degradation, thereby enabling effective centriole biogenesis. In sharp contrast to this finding, the LCL cells expressing the p.E174Tfs*4 and p.L309Pfs*9 truncations (i.e., son and daughter) did not detectably alter the levels of Cep63, Cep152, and other associating proteins (Supplementary Fig. 6c, left). A significant level of p.L309Pfs*9 was found at centrosomes, even though detection of this truncated form could have been undermined by the use of an antibody against the full-length form (Supplementary Fig. 6c, right). This suggests that the N-terminal p.L309Pfs*9 fragment, which is reported to partially retain Cep57 function^3^, may bind to Cep63 and help stabilize their associated proteins. In contrast, the ∆exon3 mutant, deprived of the Cep57–Cep63 interaction, renders not only the Cep57–Cep63–Cep152 platform itself but also its downstream centriole assembly components vulnerable to degradation in cells.

### The MVA2 syndrome-linked Cep57^∆exon 3 (68–128)^ mutant fails to promote proper centriole duplication and disengagement

**Fig. 6:**
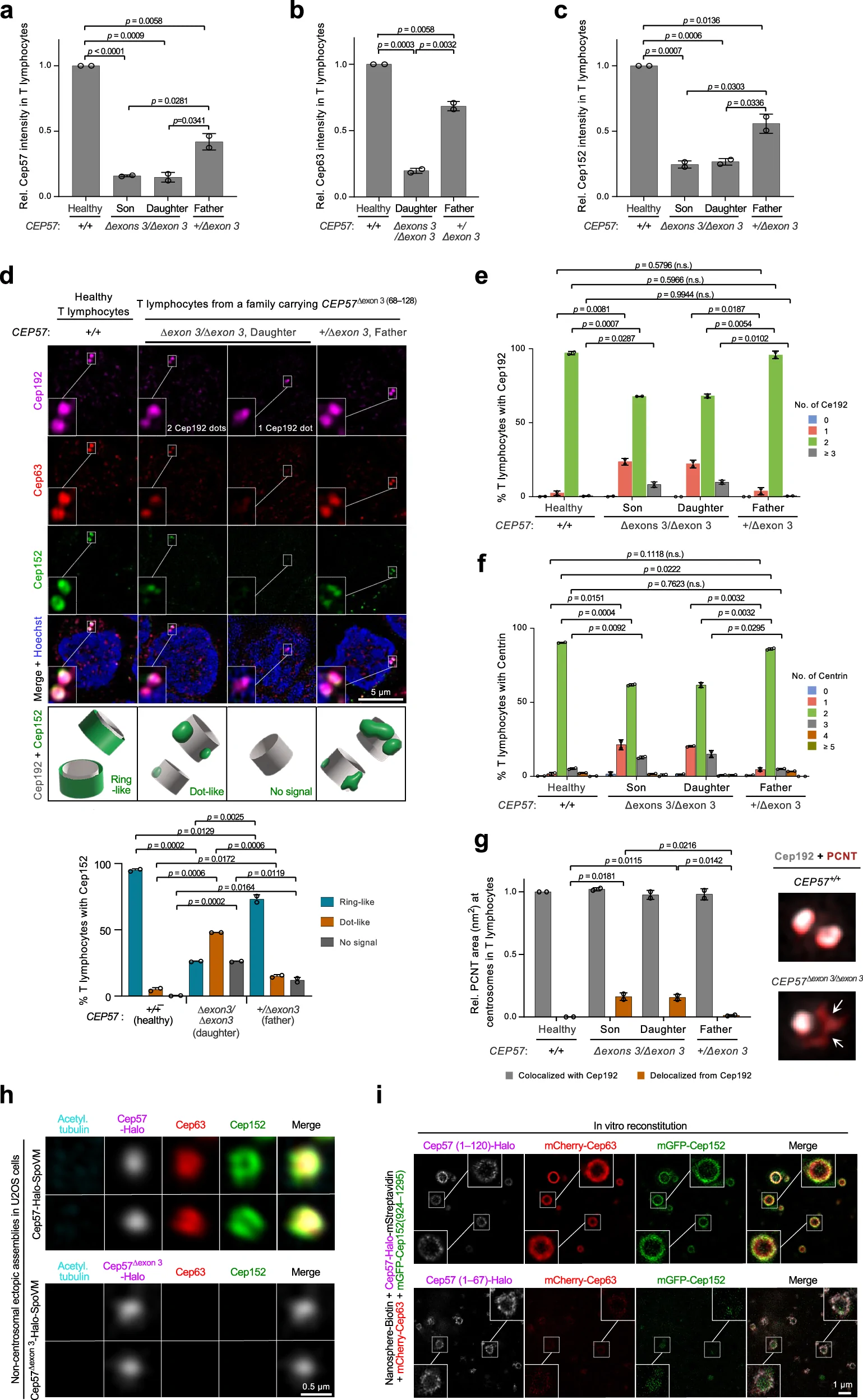
The *CEP57*^*∆exon 3*^ T cells fail to establish the pericentriolar Cep63-Cep152 platform and promote centriole duplication. **a**–**c** Quantification of recruited Cep57, Cep63, and Cep152 signal intensities using primary T cells purified from the indicated persons. Quantification was performed with confocal images of immunostained cells from two independent experiments [per experiment: for Cep57 (**a**), *n* ≥ 84 (total *n* = 177) for healthy; *n* ≥ 82 (total *n* = 166) for son; *n* ≥ 86 (total *n* = 189) for daughter; *n* ≥ 33 (total *n* = 82) for father; for Cep63 (**b**), *n* = 50 (total *n* = 100) for healthy; *n* ≥ 24 (total *n* = 55) for daughter; *n* ≥ 46 (total *n* = 94) for father; for Cep152 (**c**), *n* ≥ 81 (total *n* = 167) for healthy; *n* ≥ 68 (total *n* = 146) for son; *n* ≥ 59 (total *n* = 137) for daughter; *n* ≥ 51 (total *n* = 110) for father]. Bars, means of two experiments ± s.d.; *P* values, unpaired two-tailed *t*tests. **d** Representative 3D-SIM images and quantification of Cep152 localization in immunostained primary T cells purified from the indicated individuals. The data were obtained from two independent experiments [per experiment: *n* ≥ 202 (total *n* = 461) for healthy control; *n* ≥ 152 (total *n* = 319) for daughter; *n* ≥ 163 (total *n* = 334) for father]. Bars, means of two experiments ± s.d.; *P* values, unpaired two-tailed *t*-tests. Boxes, areas of enlargement. **e**,**f** Quantification of Cep192 (**e**) and centrin (**f**) signals was performed from two independent experiments using 3D-SIM images as in Supplementary Fig. 6f [per experiment: for (**e**,**f**), *n* ≥ 294 (total *n* = 700) for healthy control; *n* ≥ 231 (total *n* = 492) for son; *n* ≥ 255 (total *n* = 563) for daughter; *n* ≥ 165 (total *n* = 375) for father]. Bars, means of two experiments ± s.d.; *P* values, unpaired two-tailed *t*tests. **g** Quantification of PCNT signals showing the indicated localized patterns was performed from two independent experiments using 3D-SIM images as in Supplementary Fig. 6h [per experiment: *n* ≥ 114 (total *n* = 251) for healthy control; n ≥ 84 (total *n* = 198) for son; *n* ≥ 86 (total *n* = 185) for daughter; *n* ≥ 83 (total *n* = 173) for father]. Bars, means of two experiments ± s.d.; *P* values, unpaired two-tailed *t*-tests. Arrows denote PCNT signals not colocalized with the Cep192. **h** Representative 3D-SIM images showing the ability of Cep57, but not the Cep57^∆exon 3^ mutant, to recruit endogenous Cep63 and Cep152, and form ectopic (i.e., acetylated tubulin-absent) assemblies in U2OS cells. Ectopically expressed Cep57-Halo-SpoVM was detected using a HaloTag JF646 probe. The uncropped images showing multiple ectopic assemblies are provided in Supplementary Fig. 6j. **i** Representative 3D-SIM images demonstrating the ability of a nanosphere-supported Cep57(1–120), but not the exon 3 region-lacking Cep57(1–67), to promote the formation of the Cep63•Cep152(924–1295) assemblies in vitro. Cep57-Halo was detected as in (**h**). Boxes, areas of enlargement.

Immunostaining analyses revealed that, although expressed at a low steady-state level (Supplementary Fig. 6a), the ∆exon 3 mutant in the *CEP57*^*∆exon 3/∆exon 3*^ T cells (i.e., son and daughter T cells) exhibited a low but significant level of centrosome-localized signals (Fig. 6a and Supplementary Fig. 6d) when compared to those from the *CEP57*^*-/-*^ cells (Supplementary Fig. 6d, bottom). Consistent with this view, Cep63 and Cep152 also showed greatly diminished centrosome-associated signals in these cells (Fig. 6b, c). Cep152 signals were frequently uneven, dot-like, or undetectable (Fig. 6d), likely due to the reduced level of this protein localized around the centriole. In line with the critical role of the Cep57–Cep63–Cep152 platform in modulating Plk4-dependent centriole duplication^13,14,20,21,39^, the ∆exon 3 cells exhibited significantly underduplicated acetylated tubulin (Supplementary Fig. 6d, e), Cep192 (Fig. 6e and Supplementary Fig. 6f), and centrin (Fig. 6f and Supplementary Fig. 6f) signals. They also showed a low level of overduplicated centrioles (i.e., ≥ 3 Cep192 and ≥ 5 centrin signals in Fig. 6e, f), unlike the T cells from healthy control lymphocytes, which exhibited no more than 2 acetylated tubulin or Cep192 signals (i.e., unduplicated centrioles) (Supplementary Fig. 6g). Since various Cep57 or Cep63 mutants attenuated in the Cep57–Cep63 interaction did not show any detectable level of centriole overduplication (Fig. 5), the complete loss of the Cep57–Cep63 interaction by the *∆exon 3* mutation may be necessary to cause premature centriole disengagement and centriole overduplication. Expectedly, the heterozygous *CEP57*^*+/∆exon 3*^ T cells behaved largely like control WT cells from a healthy individual, although a low level of disrupted Cep152 localization was apparent (Fig. 6d).

PCNT, which is shown to interact with Cep57^15^, appears to play a critical role in mediating centriole disengagement^40^. Therefore, we closely examined whether the *CEP57*^*∆exon 3*^ mutation alters PCNT localization around the centriole in the primary T cells used above. Surprisingly, however, overall PCNT intensities were only mildly diminished in the homozygous *∆exon 3* mutant cells (Supplementary Fig. 6h), although a low but significant level of these signals was found to be localized outside of the normal Cep192 region (Fig. 6g). Moreover, the MVA-linked Cep192^4^ also showed modestly reduced signals at centrosomes (Supplementary Fig. 6i). These findings, along with the data shown in Fig. 6a–f, suggest that, unlike the p.L309Pfs*9 mutation, which primarily causes precocious centriole disengagement^3,15^, the *CEP57*^∆exon 3/∆exon 3^ cells are grossly defective in Cep57–Cep63–Cep152-dependent centriole duplication, although a degree of defect in PCNT and Cep192 localization was apparent.

### The exon 3 region of Cep57 is critical for establishing Cep57-Cep63•Cep152 assemblies in vivo and in vitro

To investigate whether the Cep57 exon 3 region is necessary and sufficient to promote the formation of the Cep63•Cep152 assemblies in cultured cells, we expressed *CEP57* WT or its respective *∆exon 3* mutant C-terminally fused with HaloTag^41^ and SpoVM (Supplementary Fig. 6j), a 26-residue-long amphipathic stage V sporulation protein M from *Bacillus subtilis*^42^. SpoVM was used to promote the formation of the Cep63•Cep152 self-assemblies at an ectopic site in cultured cells^20^. Consistent with the data shown in Figs. 4–6, Cep57 WT, but not the ∆exon 3 mutant, recruited endogenous Cep63 and Cep152 and concentrically organized them around Halo-labeled Cep57 at ectopic sites (Fig. 6h and Supplementary Fig. 6j). Under the same conditions, PCNT was not detectably recruited at the Cep57-organized assemblies (Supplementary Fig. 6k), reflecting the low or undetectable level of the Cep57–PCNT interaction^13,15^. In addition, α-tubulin signals were not detectable in these assemblies (Supplementary Fig. 6l), indicating that Cep57–Cep63–Cep152 can form assemblies independently of tubulins or MT structure.

In a related experiment, we examined whether the Cep57–Cep63 interaction can organize assemblies with Cep63 and Cep152 in vitro using purified proteins from bacterial cells. To this end, Cep57 (1–120)-Halo-mStreptavidin, containing most of the exon 3 region (residues 68–128), or its respective control Cep57(1–67) was immobilized onto biotinylated nanospheres and then incubated with mCherry-Cep63 and mGFP-Cep152(924–1295). Under the submicromolar concentrations where the formation of Cep63•Cep152 self-assemblies did not occur^20^, Cep57(1–120)-Halo-mStreptavidin, but not its respective Cep57(1–67) lacking the exon 3 region, recruited mCherry-Cep63 and mGFP-Cep152(924–1295) (Fig. 6i), displaying the localization pattern similar to that observed in Fig. 6h. This observation demonstrates that Cep57, Cep63, and Cep152 can organize into a three-protein-layered platform without the need for another protein, and that Cep57 can drive this event through an exon 3-dependent interaction with Cep63.

## Discussion

### The dual structural Cep57 links inner PCM assemblies to the centriolar MTs

**Fig. 7:**
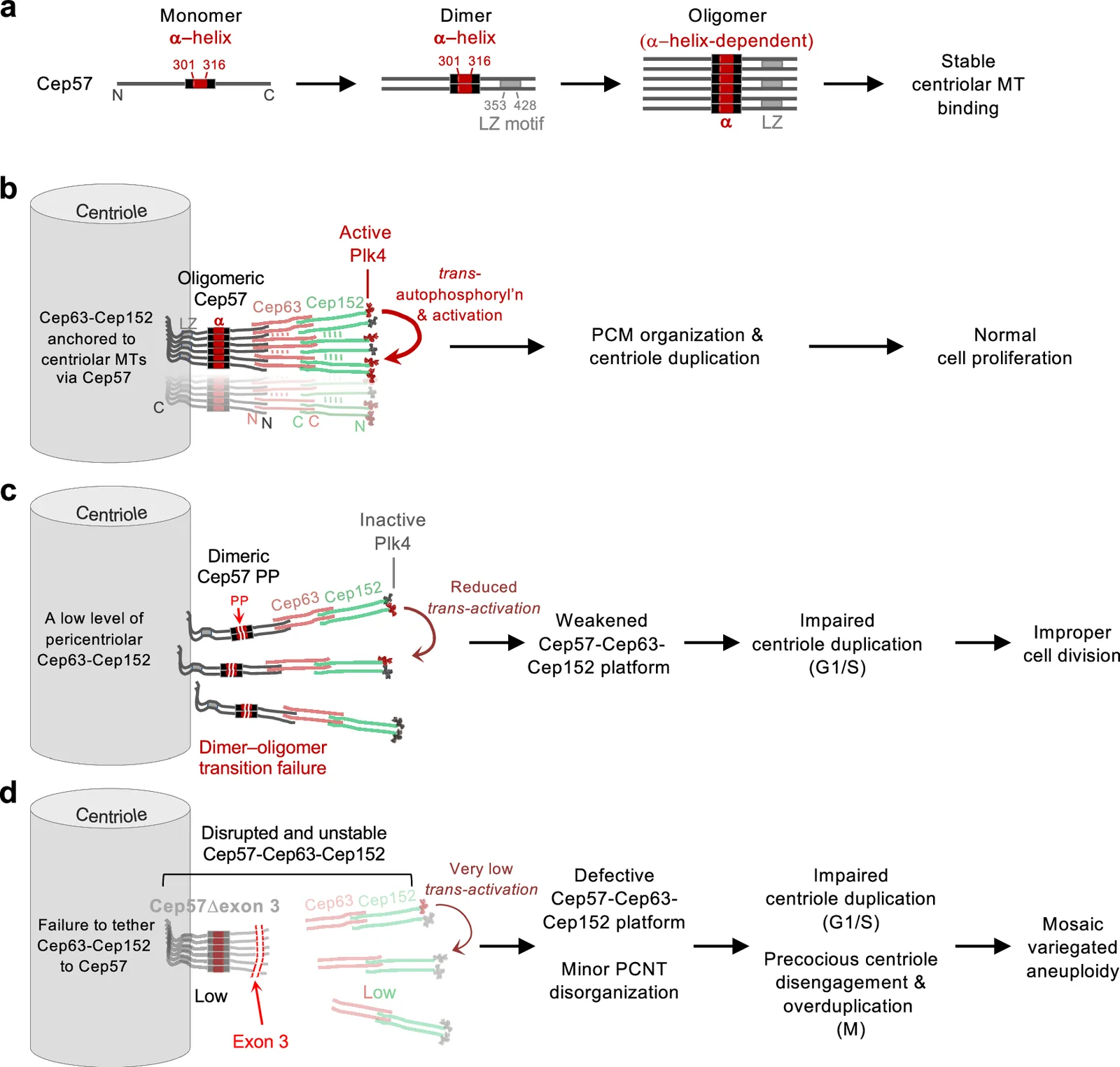
Models showing how the dual architectural Cep57 acts as a lynchpin of proper PCM organization, centriole duplication, and maintenance of chromosomal stability. **a** A schematic diagram showing how Cep57 undergoes the α-helix motif-mediated monomer-dimer-oligomer transition leading to oligomeric Cep57 formation and stable MT binding. The LZ motif, the dimeric interface of Cep57^37^. The reported LLPS of Cep57^33^, which occurs via low intermolecular affinity, could promote not only the formation of a higher-MW complex but also its spatial organization around the centriole. **b** Oligomeric Cep57 links the Cep63•Cep152 complex (and therefore its self-assembly) to the inner PCM region through its N-terminal domain’s capacity to form a stable 2:2 heterotetrameric complex with Cep63 and the C-terminal domain’s ability to bind to centriolar MTs. This ensures normal Plk4 positioning and activation, centriole duplication, and cell proliferation. Dotted lines (pink and green) between the dimeric Cep63 and dimeric Cep152 indicate the homomeric interactions^20^ that could lead to the formation of larger Cep63•Cep152 complexes. **c** The PP mutations in the α-helical (301–316) motif weaken the MT-binding activity of Cep57 and impair centriole duplication and cell division. **d** The *∆exon 3* mutation causes a failure in establishing the inner PCM Cep63•Cep152 platform due to the complete loss of its Cep63-binding activity. This results in a defect in centriole duplication and, to a lesser extent, premature centriole disengagement, thereby causing mosaic variegated aneuploidy in MVA2 patients.

Multiple deletion mutations in *CEP57*, including the N-terminal *∆exon 3*^12^ and the C-terminal c.915_925dup11 (i.e., p.L309Pfs*9)^6,7^, cause MVA2 syndrome. This suggests that both domains are required for proper Cep57 function, and that loss of either domain can critically impair the maintenance of chromosome integrity. A MINFLUX nanoscopy-based 3D localization mapping of Cep57, combined with in vitro physicochemical analyses, revealed that oligomeric Cep57 localizes at the proximal end periphery of acetylated centriolar MTs (Fig. 1). Subsequent detailed analyses revealed that while its oligomeric C-terminal domain is necessary and sufficient for binding to centriolar MTs, its N-terminal exon 3-encoded coiled-coil region functions to form a distinct 2:2 heterotetrameric α-helical bundle with the N-terminal region of Cep63 (Fig. 7). Loss of Cep57’s oligomerization capacity by disrupting the α-helix (301–316) motif crippled its centriolar MT binding (Fig. 3), whereas loss of the Cep57-Cep63 interaction hampered proper centriole duplication (Fig. 5). These findings suggest that Cep57 functions as a dual architectural scaffold with two disparate structural and functional entities critical for Cep57 to stably bind to centriolar MTs, while anchoring the Cep63•Cep152 platform to the centriolar structure. Disruption of either of these two events dysregulates Plk4-dependent centriole duplication, ultimately causing MVA2 syndrome with severe aneuploidy^3,12,15^. Hence, Cep57 functions as a lynchpin of the PCM architecture, securely linking the Cep63•Cep152 platform and its associated proteins to centriolar MTs, thereby ensuring normal centriole duplication and chromosome segregation, both of which are central to preventing aneuploidy.

### Oligomerization-defective Cep57 PP mutations impair centriole duplication but not precocious centriole disengagement

Unlike the dimeric Cep57L1-C, the α-helix (301–316)-containing Cep57 functions as an oligomer capable of offering a stable MT-binding and bundling activity (Fig. 2 and Supplementary Fig. 2). Biochemical and functional analyses performed with the oligomerization-defective Cep57 PP mutant showed that the α-helix (301–316) motif is crucial for proper centrosome localization and for recruiting downstream components required for centriole duplication (Fig. 3 and Supplementary Fig. 3). Thus, forming an oligomeric Cep57 form constitutes an essential step for binding to centriolar MTs and promoting Plk4-dependent centriole duplication (Fig. 7).

Cells bearing the compound heterozygous Cep57 truncation (i.e., p.E174Tfs*4/p.L309Pfs*9) or depleted of Cep57 by RNAi exhibit a significant level of cells undergoing precocious centriole disengagement, subsequently inducing centriole overduplication^15^. Notably, the PP mutant, which displayed a defect in centriole duplication, did not yield a significant number of cells with overduplicated centrioles (Fig. 3f and Supplementary Fig. 3e). This suggests that although compromised in Plk4-dependent centriole duplication, the dimeric PP mutant is still functional, and that depletion of Cep57 or loss of the C-terminal MT-binding activity is necessary to trigger precocious centriole disengagement and centriole overduplication.

### Two tandemly arranged Cep57•Cep63 and Cep63•Cep152 heterotetrameric bundles organize the inner PCM space

The crystal structure of the 2:2 Cep57(63–120)•Cep63(1–87) complex (Fig. 5) shows a great degree of resemblance to that of the 2:2 Cep63(502–541)•Cep152(1205–1257) complex reported previously^20^, both forming the antiparallel heterotetrameric bundles through a stretch of pairwise interactions involving hydrophobic residues from their respective backbone homodimers. These findings raise an intriguing possibility that the inner PCM is architected through the action of two tandemly placed heterotetrameric complexes—the Cep63•Cep152 complex driving the self-assembly as reported previously^20^ and the Cep57•Cep63 complex anchoring the self-assembled structure to the centriolar MTs (Fig. 7). Remarkably, both Cep57•Cep63 and Cep63•Cep152 complexes can undergo reversible self-association through tetramer–octamer–hexadecamer transition^21^ (Fig. 4d and Supplementary Fig. 4b), suggesting that they can assemble into a larger higher-order architecture through dynamic Cep57–Cep63 and Cep63–Cep152 interactions (Fig. 7). Thus, we postulate that the orchestrated action of two expansible Cep57•Cep63 and Cep63•Cep152 complexes and subsequent formation of larger MW complexes may lead to constructing a higher-order cylindrical self-assembly anchored around centriolar MTs.

A recent report showed that NuSAP1 is critical for the initial recruitment of the Cep57–Cep63–Cep152 complex to procentrioles by binding to the N-terminal domain of Cep57^43^, suggesting that it could alter the Cep57–Cep63 interaction. However, in a coimmunoprecipitation assay, NuSAP1 interacted with Cep57 only weakly when compared to Cep63 (Supplementary Fig. 6m). Further, it neither detectably inhibited nor promoted the Cep57-Cep63 interaction. These findings suggest that the capacity of Cep57–Cep63–Cep152 to form a PCM platform remains unaltered even in the presence of NuSAP1.

### Destabilizing Cep63•Cep152 and various inner PCM components by the exon 3 deletion underlie the development of the MVA2 syndrome

Studies with MVA2 patient-derived LCL cells bearing compound heterozygous p.E174Tfs*4 and p.L309Pfs*9 mutations^15^ and MEF cells containing a homozygous p.L309Pfs*9 mutation^3^ suggested that loss of the C-terminal domain of Cep57 causes gross PCM disorganization that leads to precocious centriole disengagement and centriole overduplication. In contrast to these truncation mutants, however, we found that primary T cells bearing the homozygous *∆exon 3* (residues 68–128) mutation, which precludes the formation of a heterotetrameric complex with Cep63 (Fig. 4), exhibited predominantly centriole duplication defect, although a modest level of centriole overduplication was also apparent (Fig. 6).

Immunoblotting analyses showed that T cells carrying the homozygous *∆exon 3* mutation (both son and daughter cells) exhibited only a low level (5–10% of total) of Cep63 and Cep152 and grossly destabilized inner PCM scaffolds, such as Cep192 and PCNT (Supplementary Fig. 6a). By contrast, these proteins were largely stable in the LCL cells carrying compound heterozygous p.E174Tfs*4 and p.L309Pfs*9 mutations (Supplementary Fig. 6c), suggesting that they are stabilized by the Cep57 N-terminal fragments expressed (Supplementary Fig. 6c, left) and detectably localized at centrosomes (Supplementary Fig. 6c, right). Indeed, the p.L309Pfs*9 mutant is reported to partially retain Cep57 function in mice^3^. These findings suggest that the loss of the Cep57–Cep63 interaction by the *∆exon 3* mutation, but not the loss of the centriolar MT binding by p.L309Pfs*9, renders the Cep63•Cep152 complex and its associated proteins vulnerable to degradation, resulting in a collective failure in constructing the inner PCM architecture central for Plk4-dependent centriole duplication (Fig. 7d). In support of this notion, depletion of one of Cep57, Cep63, and Cep152 in U2OS cells destabilized the other two proteins, likely as a consequence of losing the interaction between the protein depleted and its neighbor (Supplementary Fig. 6b), suggestive of the existence of a larger, interlinked Cep57–Cep63–Cep152 scaffold platform. Moreover, each depletion substantially reduced levels of various downstream components critical for centriole duplication (Supplementary Fig. 6b). This highlights the central role of the Cep57–Cep63–Cep152 platform in stabilizing various scaffolds associated with it and establishing an environment conducive to normal centriole duplication.

### Two distinct pathways lead to the development of the MVA2 syndrome

Karyotype analyses revealed that the homozygous *∆exon 3* mutant displays mosaic aneuploidies with random gains and losses of chromosomes in 31–34% of the population^12^, a level similar to that observed with the homozygous p.L309Pfs*9 mutant ( ~ 20–50% of cells)^6,44,45^. These observations suggest that the MVA2 syndrome can arise equally from depriving Cep57 of either Cep63-binding (by the *∆exon 3* mutation) (Fig. 4) or centriolar MT-binding (by the c.915_925dup11 mutation) (Fig. 2), thereby manifesting the dual structural and functional features of Cep57 (Supplementary Fig. 7). Notably, unlike precocious centriole disengagement, which can lead to multipolar spindles and chromosomal missegregation, a defect in centriole duplication caused by the *∆exon 3* mutation often triggers mitotic catastrophe. A compromised spindle checkpoint, as observed in Cep57-depleted cells^46^, and a partial destabilization and delocalization of the MVA-causing Cep192^4^ (Supplementary Fig. 6a, i) may help fuel the generation of aneuploidy in the *∆exon 3* cells by bypassing the mitotic failure and allowing continuous cell proliferation.

The results provided here highlight the key role of Cep57 in organizing PCM and regulating the tightly controlled centriole duplication process, whose abnormalities lead to improper Plk4-dependent centriole duplication, precocious centriole disengagement, and aneuploidy as observed in MVA2 patients. Cep57’s dual architectural capacity—binding to centriolar MTs and forming a heterotetrameric complex with Cep63—underpins the distinctive character of this scaffold protein in constructing a stable Cep63•Cep152 platform in the dynamic expanse of the pericentriolar space, where various mechanochemical events occur constantly.

## Methods

### Ethics statement

This study complies with all relevant ethical regulations. The study protocol was reviewed by the National Institutes of Health (NIH) Institutional Review Board (IRB) (ID: IRB002487), which determined that the research did not constitute human subjects research under DHHS and FDA regulations; therefore, IRB approval was not required. For Primary T lymphocytes: All participants provided written informed consent. Work was approved by the AIIMS institute ethics committee (Approval No. IEC-342/06.07.2018 dated 30/07/2018)

### Plasmid constructs

Construction of pCI-neo-Flag-Cep57 (pKM2797)^13^ was reported previously. To generate Flag-tagged Cep57 4 A (I69A, L73A, L76A, I80A) (pKM7917), Cep57 2 K (L73K, L76K) (pKM7918), Cep57L1 (pKM7948), Cep63-*sil* 4 A (L25A, L28A, M29A, I32A) (pKM7919), Cep63-*sil* 2 K (L28K, I32K) (pKM7920), Cep63-*sil* 2D (M1D, L5D) (pKM7921), and Cep63-*sil* Δ11 (∆1–11 residues) (pKM7922), the respective PmeI–NotI fragment was inserted into pCI-neo-Flag vector (pKM2795) digested by the corresponding enzymes. The pEGFP-Cep57 (pKM8077), pEGFP-Cep57-N(1–269) (pKM7399), and pEGFP-Cep57-C(265–500) (pKM7400) constructs were generated by cloning the BglII–SalI (for pKM8077) or BamHI–SalI (for pKM7399 and pKM7400) fragment of Cep57 into the pEGFP-C1 vector. The pEGFP-Cep57 PP (V307P, M314P) construct (pKM8080) was generated using a synthetic method (GenScript). The pEGFP-Cep57L1 construct^14^ was described previously. The pCI-neo-HA-Cep57 (pKM1234) and pCI-neo-Flag-Cep57 (pKM7147) constructs were reported in the Wei, Z. et al. paper^13^. pCI-neo-HA-Cep63 (pKM1235), pCI-neo-Flag-Cep63 (pKM2798), and pCI-neo-GFP-Cep63 (pKM4858) were described in the Kim, TS, et al. paper^20^.

Generation of a lentivirus-based pHR′.J-CMV-Flag-Cep57 WT (pKM7147) construct^13^ was reported previously. Lentiviral Flag-Cep57 4 A (I69A, L73A, L76A, I80A) (pKM7927) and 2 K (L73K, L76K) (pKM7928) mutants were generated by inserting an AscI–NotI fragment (end filled with Klenow fragment) into the pHR′.J-CMV-SV-puro vector (pKM2994) digested by AscI-PmeI. Lentiviral pHR′.J-CMV-Flag-Cep57 PP (V307P, M314P) (pKM8049), and ΔC1 (272–353) (pKM8008), were synthetically generated (GenScript). The pHR′.J-CMV-Cep63-*sil* 4 A (L25A, L28A, M29A, I32A) (pKM7923), 2 K (L28K, I32K) (pKM7924), 2D (M1D, L5D) (pKM7925), or Δ11 (∆1–11 residues) (pKM7926) constructs were generated by inserting the respective AscI–SpeI fragment into the pHR′.J-CMV-Cep63*-sil* (pKM5659)^20^ digested by the corresponding enzyme. The pHR′.J-CMV-SV-puro-NuSAP1 (pKM8312) was constructed by inserting an AscI-BamHI NuSAP1 fragment.

For ectopic Cep57-driven self-assemblies shown in Fig. 6h and Supplementary Fig. 6j, the pHR′.J-CMV-Puro-Cep57-HaloTag-SpoVM (pKM8289) and its respective Cep57∆exon 3 mutant (pKM8292) constructs were generated by inserting an AscI-NotI (e.f.) or AscI-XhoI, respectively, fragment containing the HaloTag-SpoVM tag at its C-terminal end. The HaloTag was fused to Cep57 to label with the JF646 red fluorogenic dye. The SpoVM tag from *Bacillus subtilis* containing a 26-residue-long amphipathic stage V sporulation protein M^42^ was additionally fused to promote the self-assembly property of Cep57 on the plasma membrane surface.

To generate bacterial expression constructs, pET28a (Addgene)-(His)_6_-linked maltose-binding protein (MBP)-tobacco etch virus (TEV) protease-based Cep57-C (272–500) (pKM7953), Cep57-C1 (272–353) (pKM7963), Cep57-C2 (353–450) (pKM7965), Cep57-C3 (442–500) (pKM7967), and Cep57L1-C (253–460) (pKM7954) were generated by inserting the respective AscI–XhoI fragments into a pET28a-His_6_-MBP-TEV vector (pKM7152) digested by the corresponding enzymes. The Cep57-C (272–500) PP-expressing construct (pKM8021) was generated synthetically by GenScript. The Cep57-C (277–334) (pKM8072) and Cep57-C (277–334) PP mutant (pKM8333) were constructed by inserting a respective NdeI-NotI fragment into pKM7152. The His-MBP-TEV-Cep57 (65–115) construct (pKM7332) and His-MBP-TEV-Cep57 (63–120) (pKM7224) were generated by inserting an NdeI–XhoI fragment into the pET28a vector digested with the same enzymes. The pETDuet-1-based His-Cep57 (63–120) + Cep63 (1–87) coexpression construct (pKM7287) was described previously^13^. The His-Cep57 (63–120) + Cep63 (12–87) construct (pKM7916) was generated by replacing the Cep63 (1–87) fragment in pKM7287 with Cep63 (12–87) using the NdeI and XhoI sites. The construct expressing His-Cep57 (1–120)-(GGGGS)_3_-HaloTag-GGGGS-mSA (pKM8209) or its exon 3 region-deleting Cep57 (1–67) form (pKM8208) was generated by cloning the respective BamHI–SacI fragment into the pETDuet-1 vector digested with the same enzymes. The construct coexpressing mCherry-Cep63 and mGFP-Cep152(924–1295) (pKM7545) was generated by inserting a BamHI-NotI and an NdeI–XhoI fragment, respectively, into the pETDuet-1 vector digested with the same enzymes.

All the constructs used for this study are summarized in Supplementary Table 3.

### Cell culture and transfection

Human HEK293T and U2OS cells were purchased from the American Type Culture Collection (ATCC) and cultured as instructed by the ATCC.

Primary MEF cells obtained from Cep57^+/+^ and Cep57^–/–^ mice^3^ were cultured in Dulbecco’s modified Eagle’s medium (Thermo Fisher Scientific) plus 100 mM HEPES (Thermo Fisher Scientific) after supplementing them with 10% fetal bovine serum and 1× antibiotic–antimycotic (Thermo Fisher Scientific).

Lymphoblastoid cell line (LCL) cells derived from B-lymphocytes were purchased from Coriell Institute [https://catalog.coriell.org]. The LCL cells containing MVA mutations on Cep57 (i.e., MVA2) are: GM21654 [c.520_521del (p.Glu174Thrfs*4)/c.915_925dup (p.Leu309Profs*9), [https://catalog.coriell.org/0/Sections/Search/Sample_Detail.aspx?Ref=GM21654&Product=CC]; 4-year-old son], GM21655 [c.520_521del (p.Glu174Thrfs*4)/c.915_925dup (p.Leu309Profs*9), [https://catalog.coriell.org/0/Sections/Search/Sample_Detail.aspx?Ref=GM21655&Product=CC]; 11-month-old daughter], and GM21656 [+/c.915_925dup (p.Leu309Profs*9), [https://catalog.coriell.org/0/Sections/Search/Sample_Detail.aspx?Ref=GM21656&Product=CC]; the heterozygote mother of the two MVA patients]. Control LCL cells originating from a healthy individual’s B-lymphocytes (GM24604) were included for comparative analyses. These cells were cultured as instructed on the Coriell Institute website.

Transfection was performed using either the calcium phosphate coprecipitation method for lentivirus production, PEI MAX (Polysciences) for protein expression, or Lipofectamine RNAiMAX (Thermo Fisher Scientific) for siRNA-based knockdown of gene expression. Endogenous Cep57, Cep57L1, Cep63, or Cep152 were depleted by transfecting cells with a siRNA targeting the respective gene.

All the siRNAs used for this study are listed in Supplementary Table 4.

### Primary T lymphocyte purification from the blood sample of the participants

Primary T lymphocytes from a healthy control subject and MVA2 patients used in Fig. 6 and Supplementary Fig. 6 were purified from peripheral blood mononuclear cells (PBMCs) prepared from the respective peripheral blood samples. The samples were collected from the affected siblings (son and daughter, both diagnosed with MVA2 syndrome), their father (heterozygous carrier), and an unrelated healthy control. PBMCs were isolated by Ficoll density-gradient centrifugation. T lymphocytes were subsequently purified using the Pan T-cell Isolation Kit (Miltenyi Biotec, 130-096-535) following the manufacturer’s instructions. A total of 0.3 × 10^6 ^T cells suspended in 100 µl were cytocentrifuged onto 12-mm slides at 1500 rpm for 15 min using a Cytospin™ 4 cytocentrifuge. The cells were washed with 1× PBS and fixed in 4% paraformaldehyde. The fixed cytospun cells were then processed for immunostaining and immunoblotting analyses.

### Lentivirus production and generation of stable cell lines

To generate lentiviruses expressing the gene of interest, HEK293T cells were cotransfected with pHR′-CMVΔR8.2Δvpr, pHR′-CMV-VSV-G (protein G of vesicular stomatitis virus), and a pHR′.J-CMV-SV-puro-based construct containing a respective gene. To generate stable U2OS cells expressing the gene of interest, cells were infected with lentiviruses for 24 h, selected with 3 μg/mL of puromycin (Sigma-Aldrich) for 24 h, and then transfected with the indicated siRNA for 60–72 h to deplete the respective endogenous protein before further analysis. Detailed information about lentivirus production and cell line generation is described previously^47^.

### Immunostaining

For confocal and 3D-SIM imaging, cells cultured on poly-L-lysine (Sigma-Aldrich)-coated No. 1.5 coverslips were fixed with 4% paraformaldehyde for 10 min at room temperature (RT) [Figs. 1a, 2d, 3d, 6h, and Supplementary Figs. 1a, 2a, o, q, r, 3d, e, 5b–d, f, 6c, d, g, j, k] or cold methanol for 2 min at −20 °C (Fig. 6d and Supplementary Fig. 5a,e, and 6d–f,h) and permeabilized with 0.1% Triton X-100 for 5 min at RT. Cells were blocked in PBS + 5% bovine serum albumin and then subjected to immunostaining as described previously^48^. The resulting samples were mounted with the ProLong Gold antifade (Thermo Fisher Scientific) for imaging.

For MINFLUX nanoscopy (Figs. 1b–d, 3g and Supplementary Figs. 1b–f, 3f–j), U2OS cells split onto #1.5 glass coverslip 18-mm round (Warner instruments, 64-0714) were fixed by cold methanol for 2 min, permeabilized with 0.1% Triton X-100 for 5 min at RT, and then placed in the blocking buffer for 1 h. After washing, fixed cells were reacted with a rabbit polyclonal Cep57 antibody (GeneTex) in the blocking buffer for 2 h, washed three times with PBS + 0.1% Tween-20 [Sigma-Aldrich], and reacted with an Alexa Fluor 647-conjugated secondary antibody in the blocking buffer. After washing three times, the samples were mounted with a STORM blinking buffer [50 mM Tris–HCl, pH 8.0, 10 mM NaCl, 10% (w/v) glucose, 64 µg/ml catalase (Sigma–Aldrich), 0.4 mg/ml glucose oxidase (Sigma–Aldrich), and 18.8 mM β-mercaptoethylamine (Sigma–Aldrich)]^31^.

### Confocal microscopy, 3D-SIM imaging, and analysis

Confocal and 3D-SIM images were acquired using the Zeiss ELYRA S1 super-resolution microscope equipped with an Alpha Plan-Apo 63x/1.46 oil objective, 405 nm/488 nm/561 nm/640 nm laser illumination, and standard excitation and emission filter sets. To quantify fluorescence signal intensities of immunostained proteins, confocal images acquired under the same laser intensities were converted to the maximum-intensity projection of multiple z-stacks and then analyzed using the ZEN v2.1 software (Zeiss). 3D-SIM images were processed using the ZEN black software (Zeiss).

### MINFLUX data acquisition

For 3D-MINFLUX nanoscopy analysis, images were acquired using the Abberior 3D-MINFLUX system (Abberior, Göttingen, Germany)^49^. Briefly, U2OS cells asynchronously grown on #1.5 glass coverslip 18-mm round (Warner instruments, 64-0714) were fixed with cold methanol, decorated with an anti-Cep57 (GeneTex) or anti-acetylated tubulin (Sigma-Aldrich) antibody, and then detected with an anti-rabbit or anti-mouse IgG, respectively, conjugated with two to eight molecules of Alexa Fluor 647 per IgG molecule (Invitrogen). Costained Cep152, decorated with an Alexa488-conjugated secondary antibody, was used to identify centrosomes. To utilize the built-in sample stabilization on the MINFLUX system, coverslips were incubated with gold nanoparticles (BBI Solution, SKU EM.GC150) for 1 hour, then washed extensively with PBS to remove any floating gold nanoparticles. The STORM blinking buffer described above was then added to the coverslip, and it was sealed to a concave slide with 2-part silicone glue.

Centrosomes of interest were first identified by scanning for Cep152 signals with a 488-nm laser. The region of interest was then selected for MINFLUX data acquisition. Alexa Fluor 647-decorated signals (Cep57 or acetylated tubulin) were activated using a 405-nm laser with the power initially set at 0.5% and gradually increased to 6% as required, and blinking signals in the STORM blinking buffer were detected using a 640-nm MINFLUX channel with the power set at 5%. Unless otherwise indicated, signal acquisition was performed for no more than 2 h to minimize background noise, which accumulates over time and degrades image quality. All 3D-MINFLUX images were acquired from centrioles that showed similar Alexa Fluor 647 fluorescence levels, suggesting comparable antibody labeling efficiency among centrioles.

### 3D-MINFLUX image analysis

The raw data acquired with the MINFLUX Imspector software (Abberior, Göttingen, Germany) were imported into MATLAB (version R2022b) for data visualization, filtering, and post-processing. To account for spherical aberrations caused by the different refractive indices between the sample and the immersion medium, the *z-*axis data were corrected by the scaling factor of 0.7, as recommended by the manufacturer of the MINFLUX microscope^31^. In MINFLUX, a fluorophore molecule can be repeatedly localized until it photobleaches or returns to the dark state. The same protein molecule could be recorded multiple times, as a single fluorophore can emit more than once, and 2–8 fluorophores (Alexa647) were conjugated per anti-rabbit (for Cep57) or anti-mouse (for acetylated tubulin) secondary antibody. While the centroid position of localizations in the trace, accepted as an individual molecule position, is commonly used for data analysis, here, to effectively determine the relative density map of our targets, rather than counting single molecules, we mainly used the raw localizations at the post-processing stage for analysis. This is because the localization-precision-based analysis was computationally more practical in the presented application and required fewer steps in the computational pipeline. To this end, each localization dataset corresponding to an individual centriole was filtered to remove potential noise signals by accepting only localizations with at least one neighbor within 3 nm (i.e., the 3-nm cutoff; discarding all localizations beyond the first-neighbor distance of 3 nm)^50,51^. Notably, comparative analyses between localization-precision-based (i.e., a 3-nm cutoff) and trace-based (i.e., a 5-localization cutoff; i.e., requiring at least 5 localizations in a trace) filterings yielded similar results, although the former appeared marginally more rigorous in filtering out potential noise signals (see Supplementary Fig. 1f).

A density-based clustering algorithm (DBSCAN) was used to detect densely clustered areas and separate them from the surrounding loosely clustered localizations. To take into account putative multiple localizations from the same protein, DBSCAN was performed at the epsilon (i.e., distance threshold) of 5 nm and a minimum localization density threshold (minimum localization) of 20 (ε = 5 nm, localizations ≥ 20), which selects the clusters of localizations that are bigger than 20 localizations (Figs. 1b, 3g, and Supplementary Figs. 1c, d, 3g, h,). In parallel, regions with large clusters were identified using a minimum localization of 50 (ε = 5 nm, 50 ≤ localizations ≤ 500) (Fig. 3h and Supplementary Fig. 1e, f and 3i,j). These clustered localizations were interpreted as arising from an oligomeric or multimeric form of the protein (see Fig. 3 and the text for details).

After filtering out unwanted localizations, each localization dataset was aligned with a 3D template of 9-fold circularly symmetric rods by employing a Monte Carlo (MC) randomized search^32^ followed by a self-developed 3D Iterative Closest Point (ICP) algorithm (Data 1; MINFLUX analysis codes) (see Fig. 1b). The 3D template used for fitting Cep57 localizations comprised nine 200-nm-high rods arranged to form a 300-nm-diameter circle (A 200-nm height was chosen because Cep57 localizes exclusively to the proximal end of the centriole). For a 3D template for acetylated tubulin localizations, nine 500-nm-high rods were placed on a 220-nm-diameter circle (Note that the centriole is approximately 500 nm in length). The resulting six aligned localization datasets were overlayed and visualized using the MATLAB plot3 function to yield a comprehensive composite 3D map of Cep57 or acetylated tubulin localizations around the centriole (Supplementary Fig. 1c, d).

To overlay 3D-MINFLUX maps for Cep57 and acetylated tubulin localizations (Fig. 1c and Supplementary Movie 2), the combined 3-nm cutoff localization datasets from six centrioles each (shown in Supplementary Fig. 1c, d) were concentrically aligned with the nine axes for each positioned in the same radial direction, as previously reported by the expansion STED microscopy^30^. The proximal ends of the cylindrically distributed Cep57 and acetylated tubulin localizations were visually leveled with each other (as observed in Fig. 1a and Supplementary Figs. 1a, 6j). Although the localizations of Cep57 and acetylated tubulin were acquired independently, potentially causing some displacement of signals when merging them, the resulting image in Fig. 1c, middle, provides sufficient evidence that a significant fraction of Cep57 localizations coexist (i.e., overlapped) with acetylated tubulin localizations within the same PCM space.

### Calculation of the volume of Cep57 localizations

To estimate the volume occupied by Cep57, its localizations (red dots in Fig. 1d) were enclosed within a 400 × 400 × 200 nm³ (3.2 × 10⁻¹⁷ L) bounding box populated with 100,000 uniformly distributed simulated localizations (blue dots in Fig. 1d). The volume of the Cep57 localizations is therefore a fraction of the volume of the bounding box. To determine this fraction, the nearest-neighbor distance from each blue localization to the Cep57 structure was computed, and the subset of blue localizations located within 6 nm of any red localization (95% of total Cep57 signals, excluding 5% outliers) provided the required fraction. Multiplying this fraction by the total box volume yielded an approximate volume in the PCM space within which Cep57 localizations were identified.

All the algorithms used for the MINFLUX analyses were provided in Data 1.

### Immunoprecipitation and immunoblotting analyses

Immunoprecipitation was carried out after lysing transfected cells in TBSN buffer [20 mM Tris-HCl (pH 8.0), 150 mM NaCl, 0.5% Nonidet P-40, 5 mM EGTA, 1.5 mM EDTA] as described previously^52^. Briefly, supernatants obtained from centrifugation at 15,000 × g for 15 min were incubated with an anti-Flag antibody conjugated with agarose beads (Sigma-Aldrich) for 2 h before precipitation.

Immunoblotting analysis was performed as described previously^52^ using enhanced chemiluminescence substrate (Thermo Fisher Scientific). Signals were captured using a ChemiDoc™ Imaging System (Bio-Rad Laboratories) and quantified, where indicated, using the ImageJ or Fiji software.

All the primary and secondary antibodies used in this work are listed in Supplementary Table 5.

### Protein expression and purification

Purification of His_6_-MBP-TEV-Cep57-C (272–500) (pKM7953), His_6_-MBP-TEV-Cep57L1-C (253–460) (pKM7954), and all other Cep57-C-derived mutant proteins, including His_6_-MBP-TEV-Cep57 (277–334) (pKM8072) and its V307P M314P (PP) mutant (pKM8333), was performed using E. coli Rosetta strains (Novagen) cultured and induced with 1 mM Isopropyl β-D-1-thiogalactopyranoside (IPTG) overnight at 18 °C. Cell lysates prepared in an ice-cold buffer [20 mM HEPES (pH 7.5), 500 mM NaCl, 0.5 mM TCEP] were subjected to HisTrap HP 5 mL column (GE Healthcare) and Superdex 200 10/300 GL (GE Healthcare) size exclusion chromatography (SEC). For purification of His-MBP-untagged Cep57 (277–334) and its PP mutant for circular dichroism analysis, the respective proteins above were digested with His-TEV, reversed-phase His- and MBP-trapped, and then subjected to SEC. Purified proteins were stored in the final buffer [20 mM HEPES (pH 7.5), 150 mM NaCl, 0.5 mM TCEP] at −80 °C until use. The Cep57 (63–120) (pKM7224), Cep57(63–120)•Cep63(1–87) complex (M7287), and Cep57(63–120)•Cep63(12–87) complex (M7916) were purified by HisTrap, followed by TEV digestion and SEC, and stored in the final buffer [20 mM HEPES pH 7.5, 50 mM NaCl, 5% glycerol, 2 mM 2-mercaptoethanol] at −80°C.

Cep57(65–115) (pKM7332) was expressed in an *E. coli* BL21(DE3) RIL strain (Merck) by treating the cells with 0.3 mM IPTG in an ice-cold buffer [20 mM Tris-HCl (pH 7.5), 200 mM NaCl, and 5% (v/v) glycerol]. The resulting lysates were then subjected to a Ni-nitrilotriacetic acid affinity column (QIAGEN), HiTrap^TM^ SP cation exchange chromatography column (GE Healthcare), and HiLoad 26/600 Superdex 75 pg (GE Healthcare) SEC. The proteins were stored in the final buffer [20 mM Tris-HCl (pH 7.5), 200 mM NaCl, and 5% (v/v) glycerol] at −80 °C.

The mCherry-Cep63•mGFP-Cep152(924–1295) complex (pKM7545) was lysed in the buffer containing 20 mM Tris-HCl (pH 7.5), 200 mM NaCl, and 10% (v/v) glycerol, loaded onto Ni-NTA resin(Qiagen) using a gravity-flow column, and purified on Superdex 200 increase 10/300 (GE Healthcare) column in the SEC buffer [20 mM Tris-HCl (pH 7.5), 200 mM NaCl, and 5% (v/v) glycerol]. The purified proteins were stored at −80 °C until use.

### Tubulin pulldown assay

Tubulin pulldowns performed in Fig. 2a and Supplementary Fig. 2f, g were performed by incubating tubulin or acetylated tubulin from the porcine brain with control MBP, MBP-Cep57-C, or MBP-Cep57L1-C in 0.5 mL of the tubulin buffer (80 mM PIPES, pH 6.9, 2 mM MgCl_2_, 0.5 mM EGTA) at 4 °C for 1 h. After incubating with amylose-conjugated beads for an additional 30 min, the beads were precipitated, washed four times with the tubulin buffer, and then analyzed by 8% SDS-PAGE. The resulting gel was stained with Coomassie Brilliant Blue (CBB), and the proteins of interest were quantified using the ImageJ software.

### MT co-sedimentation assay

The MT cosedimentations shown in Figs. 2b, 3c and Supplementary Fig. 2h) were performed by incubating 2.5 μM of tubulin or acetylated tubulin with 2.5 μM of the indicated protein in the tubulin buffer supplemented with 1 mM GTP, 5% glycerol, and 20 μM Taxol at 37 °C for 1 h. The samples were centrifuged at 45,000 × g at 37 °C for 45 min. The resulting supernatant and pellet fractions were dissolved in the Laemmli sample buffer and analyzed by SDS-PAGE. The amount of the protein of interest was quantified using the ImageJ software.

### Tubulin polymerization assay

Tubulin polymerization assays in Fig. 2c were carried out by incubating 100 μM of tubulin with 100 μM of MBP-Cep57-C, MBP-Cep57L1-C, or control MBP in a buffer (80 mM PIPES, pH 6.9, 2 mM MgCl_2_, 0.5 mM EGTA) supplemented with 1 mM GTP, and 5% glycerol. For positive control, a sample containing 20 μM of Taxol (instead of the proteins) was assayed under the same buffer conditions. Sample mixtures (10 µl) prepared at 4 °C were placed in a prechilled microtiter plate (Corning Cat. # 3635). Tubulin polymerization was assessed by taking readings every 10 s at 350 nm for 1 h in a plate reader pre-warmed to 37 °C.

### In vitro MT assembly and immunostaining

To examine the ability of Cep57-C (and its C1–C3 fragments) or Cep57L1-C to bind to MTs (Fig. 2d, i and Supplementary Fig. 2o), MTs were first generated by incubating 100 μM of Hilyte Fluor^TM^ 488-labeled tubulin in the tubulin buffer supplemented with 1 mM GTP and 10% glycerol at 37 °C for 20 min. After the addition of 20 μM Taxol, the samples were further incubated at 37 °C for 5 min, diluted 50 folds with a 37 °C-prewarmed tubulin buffer plus 30% glycerol and 20 μM Taxol, mounted on 8% glutaraldehyde-activated amine functionalized slide glass for 30 min at RT. After washing out unattached MTs, the slide glass was treated with a MT-blocking solution [tubulin buffer supplemented with 20 μM Taxol and 1% bovine serum albumin (BSA)] for 30 min at RT. The samples were then incubated with 30 μg of the indicated proteins for 1 h at RT and subjected to anti-MBP immunostaining using an anti-MBP antibody and an Alexa 594-conjugated secondary antibody in the MT blocking buffer. The resulting samples were mounted with the SlowFade and subjected to confocal imaging.

To examine the ability of Cep57-C to induce MT stabilization and bundling (Fig. 2e and Supplementary Fig. 2i, j), 100 μM of Hilyte Fluor^TM^ 488-labeled tubulins were mixed with the indicated proteins in the tubulin buffer supplemented with 1 mM GTP and 10% glycerol and incubated at 37 °C for 30 min. After 30 min, 20 μM of Taxol was added to the reaction mixture and incubated for an additional 5 min at 37 °C. The resulting samples were diluted 50 folds, mounted with the SlowFade, and subjected to confocal imaging.

### Negative staining TEM

To visualize the overall morphology of the indicated proteins associated with MTs (Fig. 2f and Supplementary Fig. 2k) by negative-staining TEM, MTs were assembled with 100 μM of tubulin as above in the presence of 5 μM of the indicated proteins in the tubulin buffer supplemented with 1 mM GTP and 5% glycerol. The resulting samples were diluted 10 times with the prewarmed (37 °C) tubulin buffer plus 30% glycerol, loaded onto a positively charged carbon film on a copper grid for 1 min, washed with the tubulin buffer three times, stained with 1% freshly made uranyl acetate solution for 1 min, dried at RT, and then imaged using a transmission electron microscope (Tecnai 12 120 kV Electron Microscope with K2 summit).

To negatively stain Cep57-C (Supplementary Fig. 2l), the protein (150 nM) was loaded onto a plasma-clean carbon film on a copper grid for 1 min, washed with water three times, stained with 1% uranyl acetate for 1 min, dried after whisking away excess solution, and then imaged.

### Sedimentation velocity analytical ultracentrifugation

Sedimentation velocity experiments with purified Cep57(63–120)•Cep63(1–87) (pKM7287) were conducted at 50,000 rpm and 4 °C using an An-50 Ti Rotor (200,000 x *g* at 7.156 cm) on a Beckman Coulter ProteomeLab XL-I analytical ultracentrifuge as described previously^21^. A stock solution of the complex in 50 mM NaCl, 20 mM MES pH 6.4, 2 mM TCEP, and 5% (v/v) glycerol, along with a matching buffer, was used to prepare a series of solutions at concentrations ranging from 30 to 220 μM. Time-corrected sedimentation data were analyzed in SEDFIT^53^ in terms of a continuous c(*s*) distribution of sedimenting species. The partial specific volume of the Cep57•Cep63 complex was determined based on the amino acid composition using SEDNTERP^54^. The solvent density and viscosity were determined experimentally at 20°C using an Anton Paar DMA 5000 density meter and an Anton Paar AMVn rolling-ball viscometer, respectively, and corrected to values at 4 °C. Sedimentation coefficients were corrected to standard conditions in water at 20 °C, s20,w. Sedimentation velocity analysis of the Cep57(63–120)•Cep63(12–87) complex (pKM7916) was performed under the same conditions as its parental form (pKM7287) above.

Sedimentation velocity analyses for MBP-Cep57-C (M7953), MBP-Cep57L1-C (M7954), and MBP-Cep57 (277–334) (pK8072) were performed at 50,000 rpm, 4 °C in 20 mM Hepes, pH 7.5, 150 mM NaCl, 10% glycerol, along with a matching buffer.

### Sedimentation equilibrium analytical ultracentrifugation

Sedimentation equilibrium experiments were carried out to study the self-association of Cep57(63–120)•Cep63(1–87) (pKM7287) at pH 6.4 and 4 °C. A stock solution of the protein in 50 mM NaCl, 20 mM MES pH 6.4, 2 mM TCEP, and 5% (v/v) glycerol was used to prepare samples at 35 µM, 70 µM, 145 µM, and 275 µM. Samples were loaded in pre-chilled, two-channel, sector-shaped cells and placed into a pre-chilled An50-Ti rotor. Depending on the loading concentration, 12 or 3 mm pathlength centerpieces were used. Sedimentation equilibrium experiments were conducted at 4 °C on a Beckman Optima XL-A analytical ultracentrifuge as described previously^21^, and data were collected at 8,000, 14,000, and 22,000 rpm. Sedimentation equilibrium data collected at different speeds and absorbance wavelengths (280 nm and 250 nm) were analyzed globally in terms of various self-association models in SEDPHAT^55^ with the implementation of mass conservation. The model that described the data best was a reversible monomer–dimer–tetramer self-association where the monomer unit represents the heterotetrameric 2:2 Cep57(63–120)•Cep63(1–87) complex. Data were also collected for a sample at 840 µM in a 1.5-mm pathlength cell. Here, sample losses were observed during the experiment, indicative of the cooperative nature of the self-association. The extinction coefficient value at 280 for the Cep57(63–120)•Cep63(1–87) complex was calculated based on the amino acid composition in SEDNTERP. The extinction coefficient at 250 nm was refined in SEDPHAT.

### Circular dichroism

The circular dichroism (CD) spectra for the indicated protein complexes were measured in a Jasco J-715 spectropolarimeter (Jasco International Co.) following standard protocol. For each sample, triplicate CD spectra were measured from 195 to 260 nm using a 1-mm path-length cuvette at a 1-nm spectral bandwidth and a 1-second response time. After subtracting the measured buffer spectra, the resulting spectra were converted into molar ellipticity (deg•cm^2^•dmol^−1^) units and plotted. Absorbance profiles obtained from three independent measurements are also provided (Supplementary Fig. 3a, left).

### Interferometric scattering mass spectrometry (iSCAMS)

The iSCAMS measurement was performed using the Refeyn TwoMP mass photometer (Refeyn Ltd.), as described previously^21^. Briefly, the buffer (20 mM HEPES, 150 mM NaCl, 5% glycerol, 0.5 mM TCEP, pH 7.5) filtered through a syringe filter with 0.02 μm pore size (Anotop 10, Whatman) was used for all analyses. All samples diluted to the indicated concentrations were measured immediately after dilution.

### SEC-MALS

SEC-MALS for the indicated proteins in Fig. 4b was performed at 4°C using an in-line high-performance liquid chromatography (Agilent Technologies 1260 Infinity), Superdex 75 10/300 GL (Cytiva), and MALS system (Wyatt DAWN HELEOS II and OPTILAB T-rEX) in the final buffer [20 mM HEPES, 50 mM NaCl, and 2 mM 2-mecaptoethanol]. Data were analyzed using Astra software (Wyatt, version 7.3.1).

### Crystallization and data collection

The crystals of the Cep57(65–115) dimer were obtained via the sitting-drop vapor diffusion method at 18 °C after mixing and equilibrating 1 μL of the protein solution (10 mg/mL) with 1 μL of a precipitant solution [500 mM magnesium formate dihydrate and 100 mM sodium acetate trihydrate (pH 4.5)]. Platinum-derivative crystals were obtained by soaking native crystals in the mother liquor containing 0.5 mM platinum potassium thiocyanate for 3 days at 18 °C. Diffraction data were collected using beamline 7 A at the Pohang Accelerator Laboratory, Republic of Korea, and processed with the *HKL*2000^56^ program.

The crystals of the Cep57(63–120)•Cep63(1–87) complex were obtained via the sitting-drop vapor diffusion method at 20 °C after mixing and equilibrating 1 μL of the protein solution at 15 mg/mL in 20 mM Tris (pH 7.5), 50 mM NaCl, 5% glycerol, 0.5 mM TCEP, and 30 mM NDSB-195 with 1 μL of a precipitant solution [12% PEG4000, 5% PEG1000, 75 mM sodium citrate (pH 5.8), 75 mM ammonium sulfate, 25 mM lithium sulfate monohydrate, 25 mM sodium citrate tribasic dihydrate (pH 5.5), and 15% glycerol]. Diffraction datasets were collected at 100 K with the 22-ID beamline at the Advanced Photon Source, Argonne National Laboratory, Lemont, IL. The datasets were indexed and integrated with the XDS software package^57^, and scaled with Aimless from the CCP4 software suite^58^.

### Structure determination

The complex structure was determined using the single-wavelength anomalous diffraction method in AutoSol. The Coot^59^ and PHENIX^60^ programs were used to build and refine models, respectively. The coordinates and structure factors of the Cep57(65–115) dimer have been deposited in the Protein Data Bank (PDB code: 21JQ). The crystallographic data are summarized in Supplementary Table 1.

The structure of the Cep57(63–120)•Cep63(1–87) complex was solved by molecular replacement using the Cep57(65-115) dimer as a search model in the Phaser-MR package. The Cep 63 residues were built using Rosetta^61,62^. The structure was further refined using PHENIX^60^ and Coot^59^. The crystallographic data (PDB ID:9I7Q) are summarized in Supplementary Table 2.

### In vitro reconstitution of the Cep57–Cep63–Cep152 assemblies

Bacterially expressed His-Cep57(1–120)-(GGGGS)_3_-HaloTag-GGGGS-monomeric streptavidin (mSA) fusion and its respective exon 3-region-lacking Cep57(1–67) control were partially purified by precipitating them with biotinylated spherical gold nanoparticles (Nanopartz Inc., CO). The GGGS linkers were provided to minimize potential structural constraints among Cep57, HaloTag, and mSA. After incubating the nanoparticle-bound Cep57 with mCherry-Cep63 and mGFP-Cep152(924–1295) at 10 μM each for 2 h, the resulting particles were washed with PBS twice and then incubated with 200 nM of JF646 (magenta) to label Cep57-HaloTag-mSA. The resulting samples were washed, covered with SlowFade (Thermo Fisher Scientific), and imaged using a Zeiss ELYRA S1, as described above.

### Statistical analysis and reproducibility

All the experiments were performed at least twice independently. All values are given as a mean of n ± s.d. The statistical details, including the definition and value of n (e.g., number of experimental replicates, target molecule signal intensity, etc.) and standard deviations, are provided in figures, corresponding figure legends, and results. Statistical significance for all the graphs was assessed using a two-tailed *t*-test in GraphPad Prism. The p-values are displayed on the graph to save space.

For MINFLUX image analyses, all raw localizations obtained after 3-nm cutoff filtering, as well as the additional filtered localizations obtained after density-based clustering (i.e., DBSCAN) analyses for Cep57 and acetylated tubulin signals, are provided in the Source Data file. Outliers identified by box plot analyses were excluded.

### Reporting summary

Further information on research design is available in the Nature Portfolio Reporting Summary linked to this article.

## Supplementary information

Supplementary Information

Transparent Peer Review file

Reporting summary

Description of Additional Supplementary Files

Supplementary Movie 1

Supplementary Movie 2

Supplementary Movie 3

## Source data

Source Data

## Data Availability

All the raw data used for quantification and statistical analyses of 3D-SIM, confocal, and immunoblots are provided in the Source Data file. They are deposited in Mendeley Data and are available without restriction through the repository DOI [10.17632/zds6wjb2yc.1]. The atomic coordinates and structure factors have been deposited in the Protein Data Bank (PDB) under accession codes 21JQ [10.2210/pdb_000021jq/pdb] (Structure of Cep57 residues 65–115) and 9I7Q [10.2210/pdb_00009i7q/pdb] (Structure of the Cep57-N•Cep63-N complex). Source data are provided with this paper.

## Code availability

All algorithms used for analyzing MINFLUX images are provided in the Supplementary Information. The custom algorithms developed for MINFLUX data analysis have been deposited in the Mendeley Data public repository. All source immunoblots and source data underlying the quantitative analyses have also been deposited in the same repository. These materials are available without restriction through the repository DOI [10.17632/zds6wjb2yc.1].
