# Supplementary material for: Cep57 organizes pericentriolar material through dual architectural modules and prevents human mosaic variegated aneuploidy
Source: Nat Commun. 2026 Sep 30;17:11262. doi: 10.1038/s41467-026-77795-4 (PMC13720213; doi:10.1038/s41467-026-77795-4)

Supplemental information

Dual architectural Cep57: A lynchpin for organizing pericentriolar materials and preventing mosaic variegated aneuploidy in humans

Jong Il Ahn[1†] , Jung-Eun Park[1†], Hye-Yeoung Yun[1], Yan Zeng[1], Klara Kirsch[1], Vipin Kumar[1], Bonsu Ku[2], Arlene Bartolome[1], Harsha Ravishankar[1], Ruchi Bhardwaj[3], Rodolfo Ghirlando[4], Mahaiwon Shadang[3], Mohamadreza Fazel[5], David A. Ball[5], Jessica Matthias[6], Seung Jun Kim[7], Neerja Gupta[8], Jayanth Kumar Palanichamy[3], Tatiana S. Karpova[5], Kyung S. Lee[1]*

<u>One combined file:</u>

Supplementary Figures 1 – 7

Supplementary Tables 1 – 5

Supplementary Movies 1 – 3

Supplementary References

Source files for immunoblotting analyses

<u>Additional separate files:</u>

Source files for all quantifications (xlsx file)

All algorithms used for analyzing the 3D-MINFLUX localization data (csv file)

Ahn, J., et al. Supplementary Fig. 1

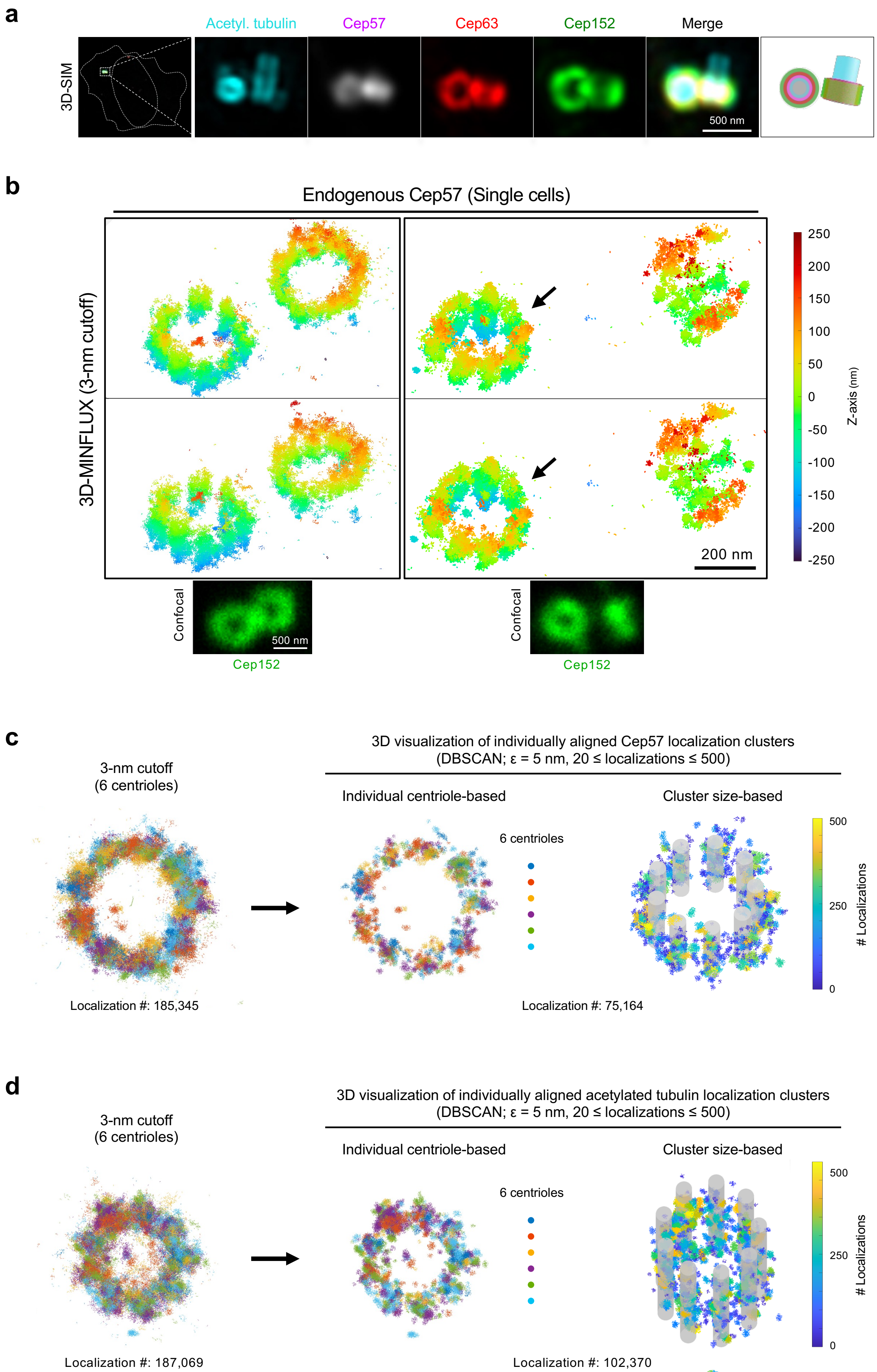

e

Large-cluster analysis

	**Endogenous Cep57** (2 sets; n = 6 centrioles/set)
Total localizations (3-nm cutoff)	308,917
Total large-cluster localizations (ε = 5 nm, 50 ≤ localizations ≤ 500)	33,646
Clustered localizations/centriole	2,803

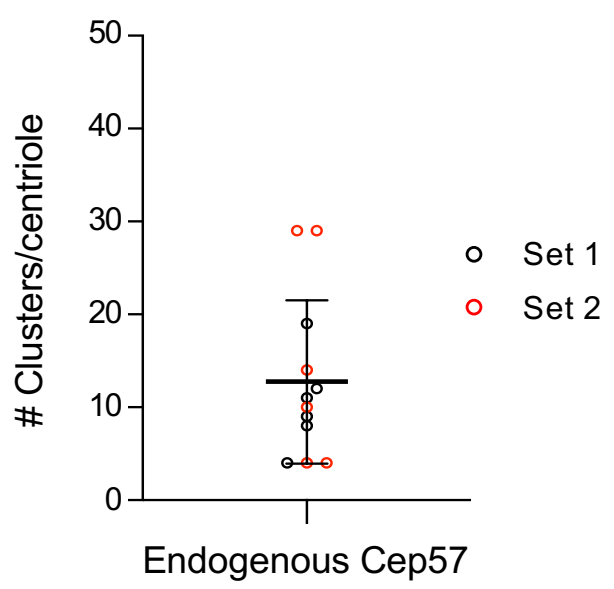

f

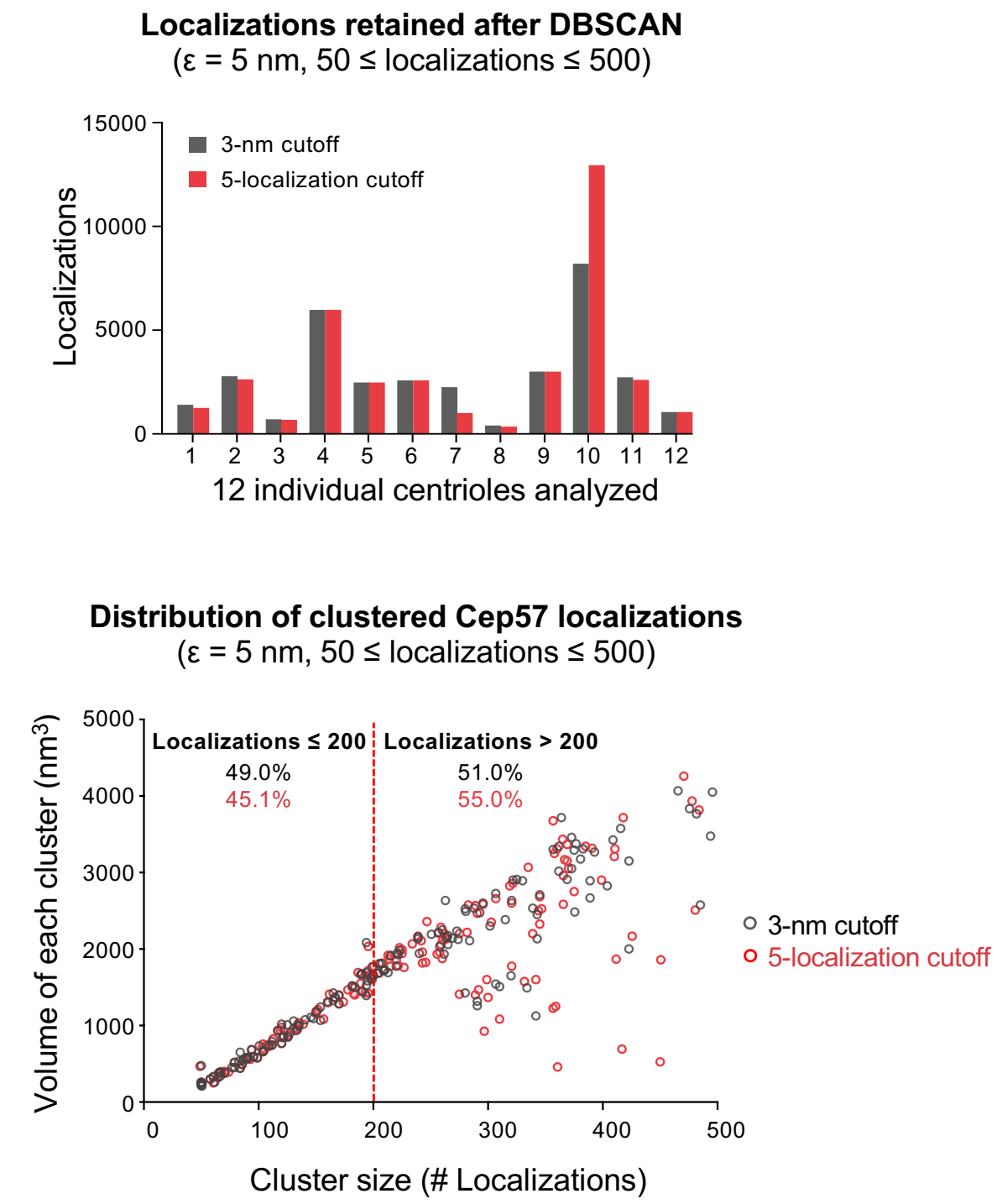

Supplementary Fig. 1. 3D-MINFLUX-based mappings and cluster analyses of Cep57 and acetylated tubulins in U2OS cells. **(a)** 3D-SIM images from an immunostained U2OS cell, showing Cep57, Cep63, and Cep152 signals concentrically localized at the proximal end of the acetylated tubulin-decorated centriole. A schematic diagram (right) shows the localization patterns of all four proteins. **(b)** Two representative 3D-MINFLUX images (shown at two angles) displaying the localizations of Cep57 in U2OS cells immunostained with an anti-Cep57 antibody and Alexa Fluor 647–conjugated anti-rabbit antibody. The arrows indicate the Cep57 localizations belonging to a single centriole used to generate the image shown in Fig. 1b, top, via MC simulation and ICP alignment. The confocal images (bottom) are anti-Cep152 signals used to locate the centrioles for the 3D-MINFLUX imaging shown above. **(c** and **d)** Step-by-step processes depicting how 3D-MINFLUX raw localizations acquired from six mother centrioles were filtered by applying a 3-nm cutoff and DBSCAN (left and middle). The 3D maps of clustered Cep57 (**c**, right) and acetylated tubulin (**d**, right) signals were generated by combining individually DBSCANed localization datasets and aligning them using MC and ICP algorithms. Clusters are color-coded based on the number of localizations per cluster (20-to-500 localizations). **(e)** Summarized data showing quantified clusters of the Cep57 signals. The graph showing Cep57 cluster numbers per centriole is based on two independent datasets comprising six mother centrioles (total n = 12). **(f)** Analyses of localizations (top) and large clusters (localizations > 200; bottom) of endogenous Cep57 used in (**e**; 12 individual centrioles) show that, except for #10 centriole, filtering with a 3-nm cutoff yielded similar values than those with a 5-localization cutoff.

Ahn, J., et al. Supplementary Fig. 2

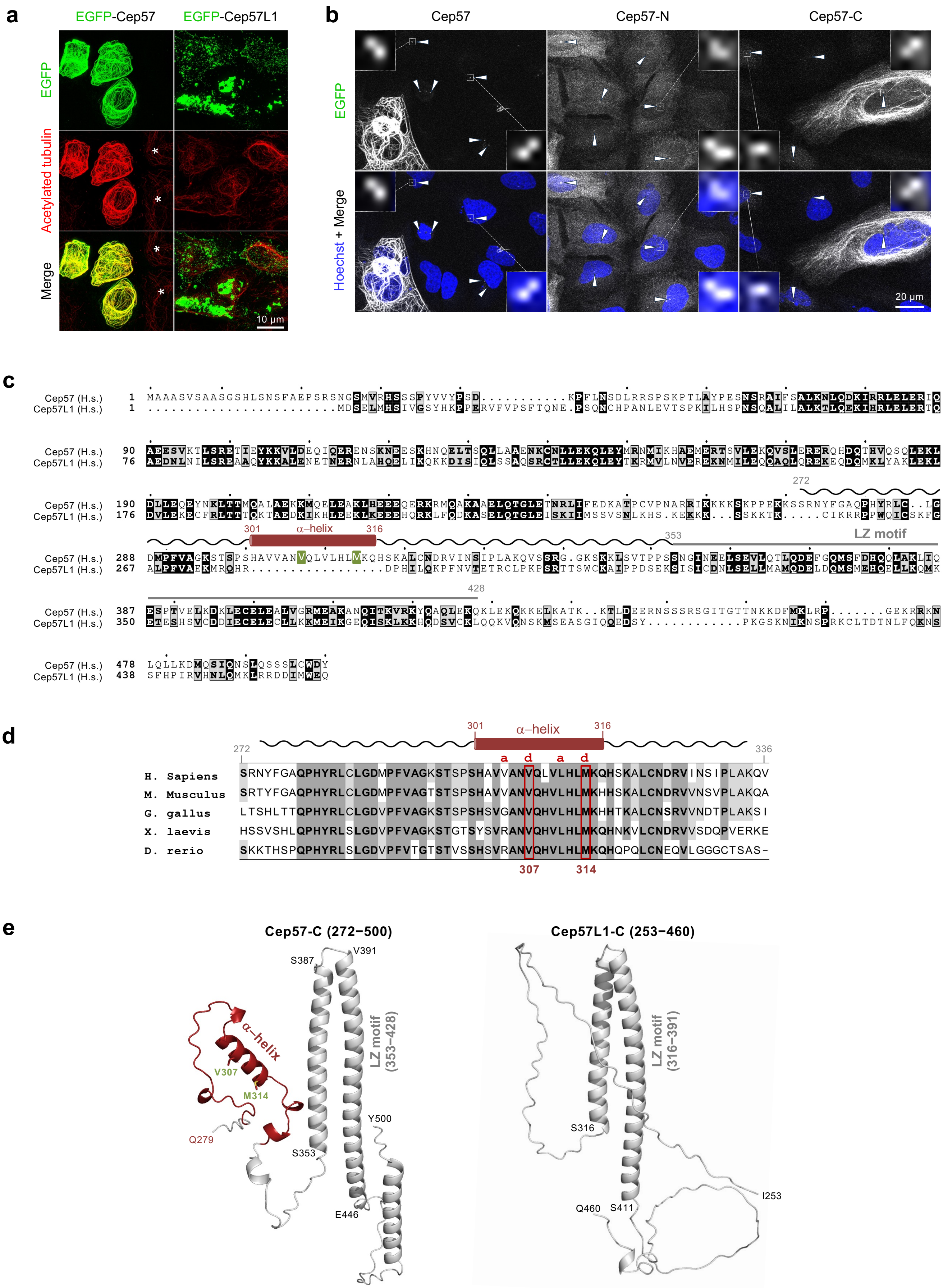

Ahn, J., et al. Supplementary Fig. 2

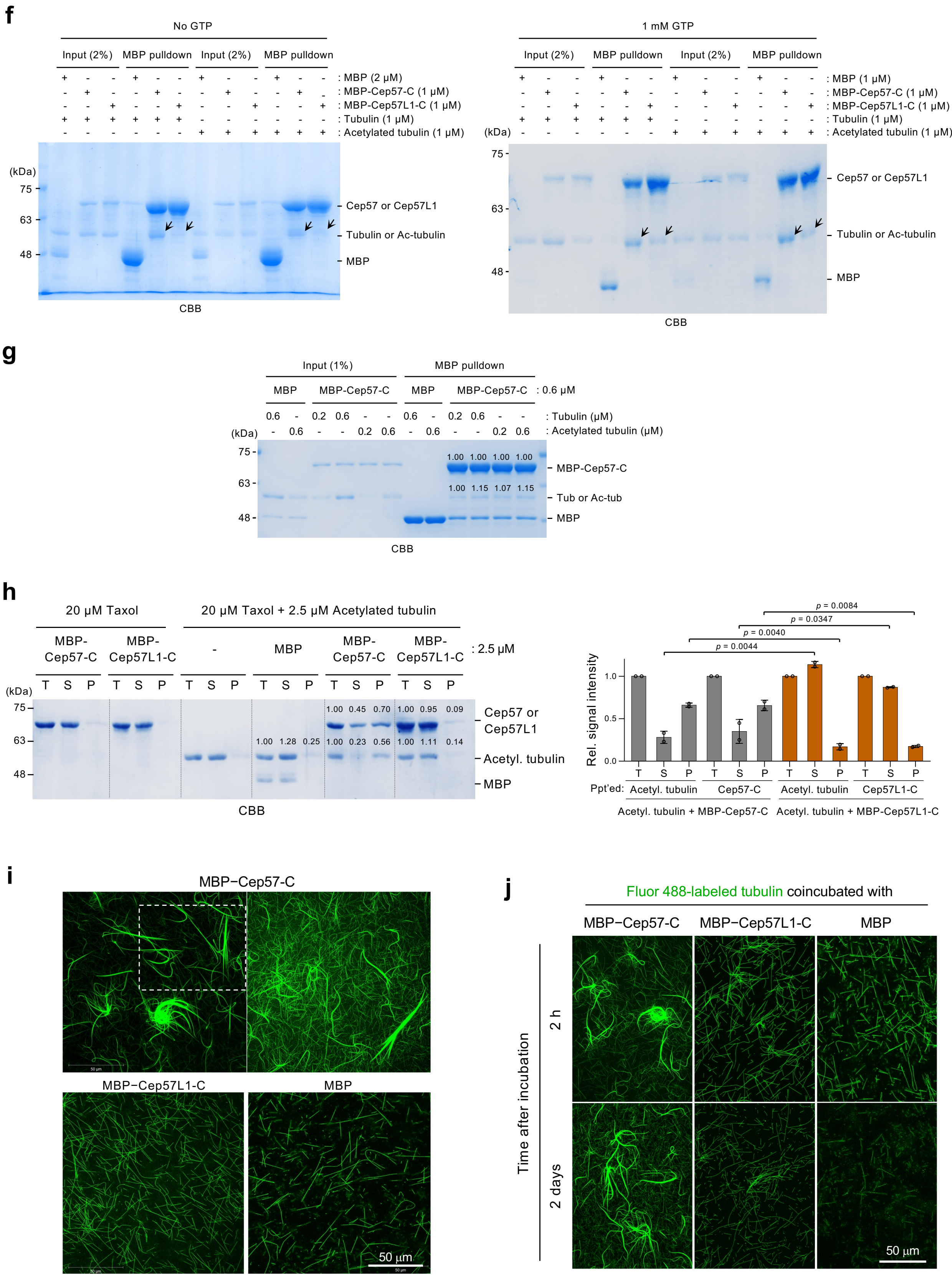

Ahn, J., et al. Supplementary Fig. 2

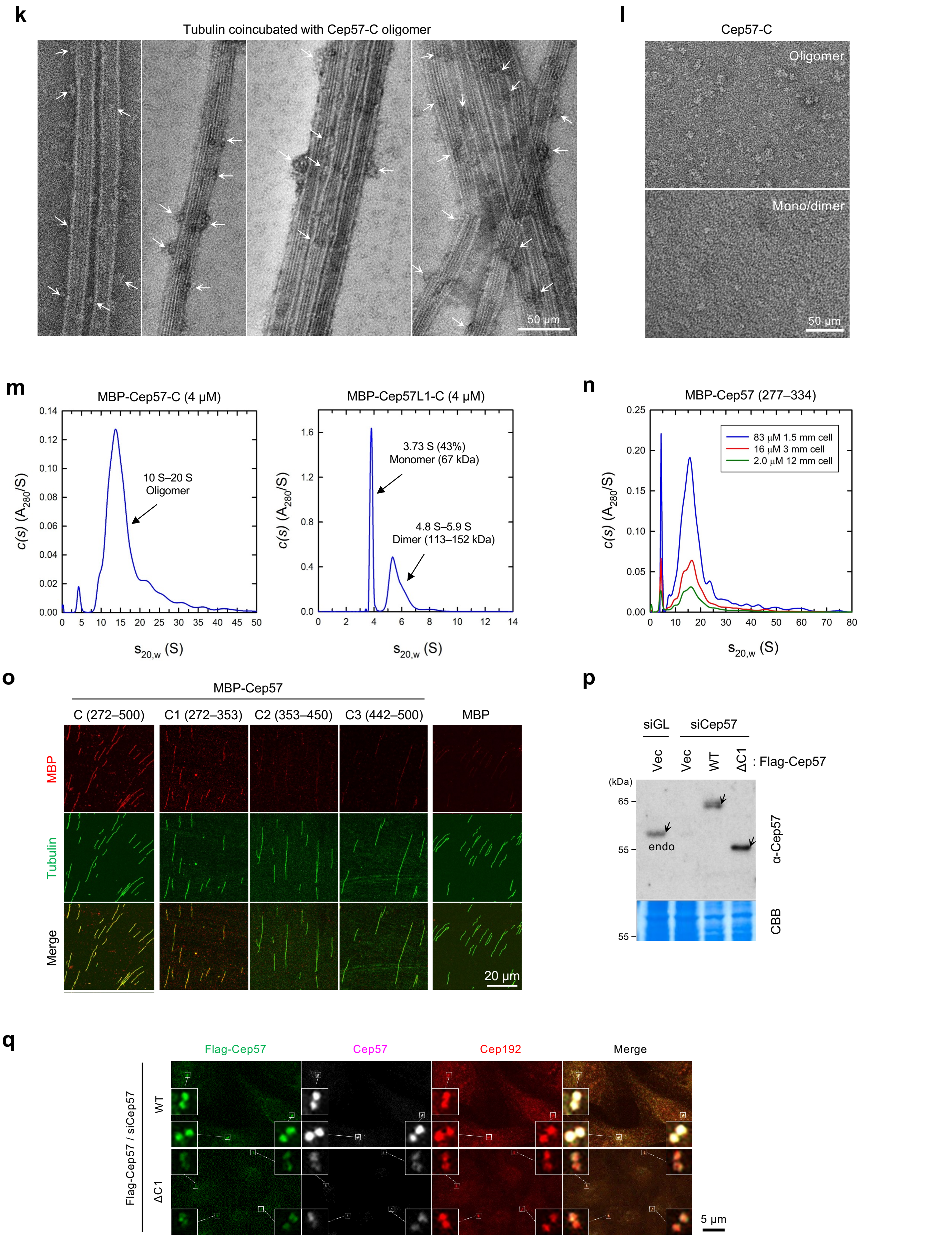

Ahn, J., et al. Supplementary Fig. 2

r

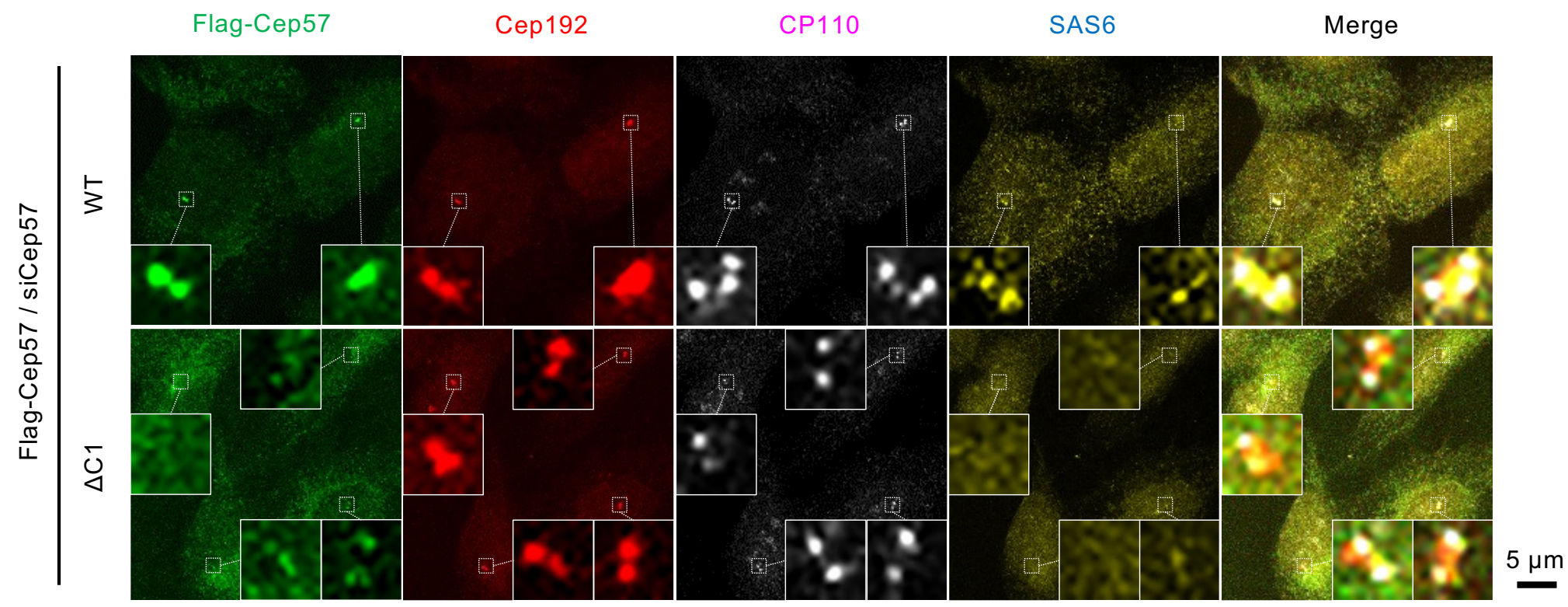

Supplementary Fig. 2. The α-helix motif is critical for Cep57's ability to bind to tubulins/MTs and localize to centrosomes. **(a)** Confocal analyses for U2OS cells transfected with EGFP-fused Cep57 or Cep57L1 and immunostained with an acetylated tubulin antibody. Asterisks, untransfected cells. **(b)** Confocal analyses for U2OS cells transfected with EGFP-fused Cep57 full-length, Cep57-N, or Cep57-C. Arrowheads, centrosome-localized signals. Boxes, areas of enlargement. **(c)** Comparative primary sequence analysis between human Cep57 (NP_055494.2) and its paralog Cep57L1 (NP_776191.1). Identical residues are boxed in black, and conserved residues are boxed in gray. The unique α-helix (residues 301–316) is marked (a dark-red bar). The two residues (V307 and M314), which are critical for the integrity of the α-helix, are marked in green. **(d)** Multiple sequence alignment for the α-helix region of human Cep57 and its orthologs performed using ClustalW software. The primary amino-acid sequences of Cep57 orthologs used for the analysis are: *H. Sapiens* (NP_055494.2), *M. Musculus* (NP_080941.3), *G. gallus* (XP_417188.2), *X. laevis* (XP_018105836.1), and *D. Rerio* (NP_001074152.1). The predicted **a** and **d** positions of the helical wheel for the residues 301–316 and the two critical V307 and M314 residues are indicated in red boxes. **(e)** Predicted structures of Cep57-C (272–500) and Cep57L1-C (253–460) generated by AlphaFold2 are shown. The α-helix (residues 301–316) region and an apparent leucine-zipper (LZ; residues 353–428) motif [1] are shown. The (353–387) region was proposed to mediate the formation of an anti-parallelly arranged Cep57 homodimer [2]. **(f)** Representative MBP pull-downs performed under the conditions described in Methods are shown. Amylose bead-associated proteins separated by sodium dodecyl sulfate–polyacrylamide gel electrophoresis (SDS-PAGE) were stained with Coomassie Brilliant Blue (CBB). Arrows, tubulins or acetylated tubulins coprecipitated by MBP-Cep57-C or MBP-Cep57L1-C under no GTP (left) or 1 mM GTP (right) conditions. The signal intensities of coprecipitated tubulins quantified from three independent (no GTP) or two independent (1 mM GTP) experiments were used to generate Fig. 2a. Other raw SDS-PAGE gels used for the quantifications are provided in the Source file. **(g)** Detailed MBP-Cep57-C pulldowns performed similarly as in **(f)** to closely compare its binding levels to tubulin and acetylated tubulin. Numbers, relative signal intensities. **(h)** MT co-sedimentation assays performed with acetylated tubulins are shown after SDS-PAGE and CBB staining. The assays were carried out under the same conditions as in Fig. 2b. Numbers, relative signal intensities. Quantified data for the fractions (i.e., T, S, and P) of the last two samples obtained from two independent experiments are shown (right). Bars, means ± s.d.; *P* values, unpaired two-tailed *t*-tests. **(i)** Additional images of Hilyte Fluor 488-labeled MTs polymerized in the presence of the indicated proteins, and then stabilized by the addition of 20 μM Taxol. White box: an area used to generate Fig. 2e. **(j)** Time-dependent confocal imaging of Hilyte Fluor 488-labeled MTs polymerized in the presence of MBP-Cep57-C, MBP-Cep57L1-C, or control MBP for 30 min, and then stabilized by the addition of 20 μM Taxol. The samples were prepared similarly to those in Fig. 2e. Note the Cep57-C-bundled MTs remained stable for 2 days, whereas the Cep57L1-C-associated and control MBP-treated MTs weakened over time (compare the images taken at 2 h and 2 days). **(k)** Negative staining TEM images showing the ability of oligomeric MBP-Cep57-C to associate with MTs in vitro. Tubulin was polymerized in the presence of the oligomeric MBP-Cep57-C and subjected to negative staining as in Fig. 2f (see Methods for details). Arrows, Cep57-C bound to MTs. **(l)** Negatively stained TEM images showing MBP-Cep57-C oligomeric and monomeric/dimeric forms purified in Fig. 2g. **(m)** Sedimentation velocity absorbance c(s) profile for MBP-Cep57-C shows a polydisperse distribution of material (10 S to 20 S). In contrast, MBP-Cep57L1-C shows the presence of a monomer (3.73 S; 43%) and reduced amounts of faster-sedimenting material (4 S to 8 S), suggesting a weak self-association in which the oligomers are in fast exchange with the monomer. **(n)** Sedimentation velocity absorbance c(s) profile for MBP-Cep57 (277–334) showing polydisperse distribution similar to that of MBP-Cep57-C. **(o)** Confocal imaging and quantification of anti-MBP signals associated with Hilyte Fluor 488-conjugated MTs. Experiments were carried out as described in Fig. 2d. Quantified data are provided in Fig. 2i. **(p–r)** Immunoblotting showing U2OS cells stably expressing Flag-Cep57 WT or its respective ΔC1 mutant (**p**) and subsequent confocal imaging displaying the localization patterns of Flag (**q**) and SAS6 and CP110 (**r**) signals. CBB in (**p**), Coomassie Brilliant Blue–stained membrane. The expressed Flag-Cep57 was detected with anti-Flag and anti-Cep57 antibodies (**q**), and the signal levels were similar. Anti-Cep192 signals mark the locations of endogenous centrosomes (**q**, **r**). Boxes, areas of enlargement.

Ahn, J., et al. Supplementary Fig. 3

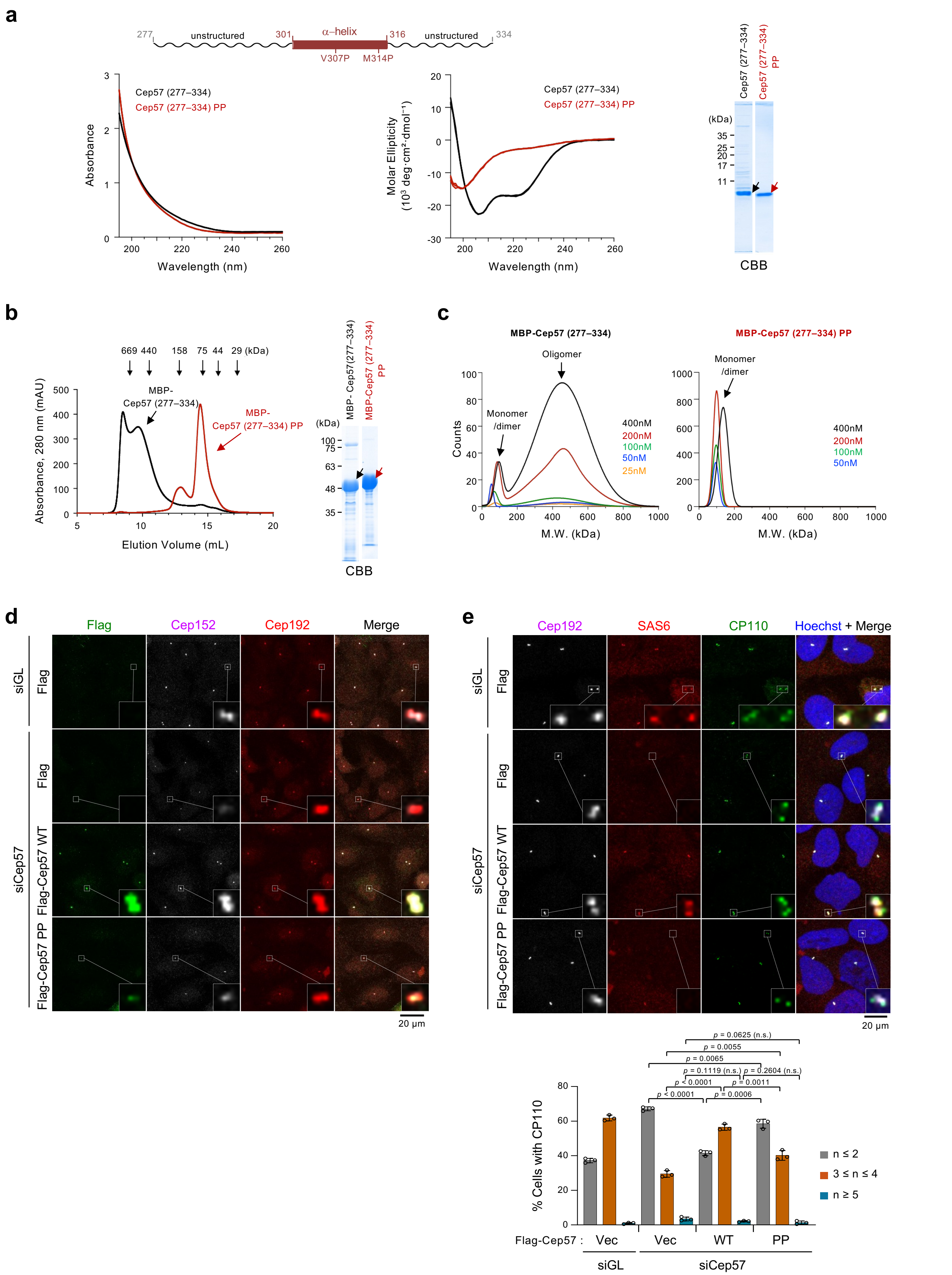

Ahn, J., et al. Supplementary Fig. 3

f

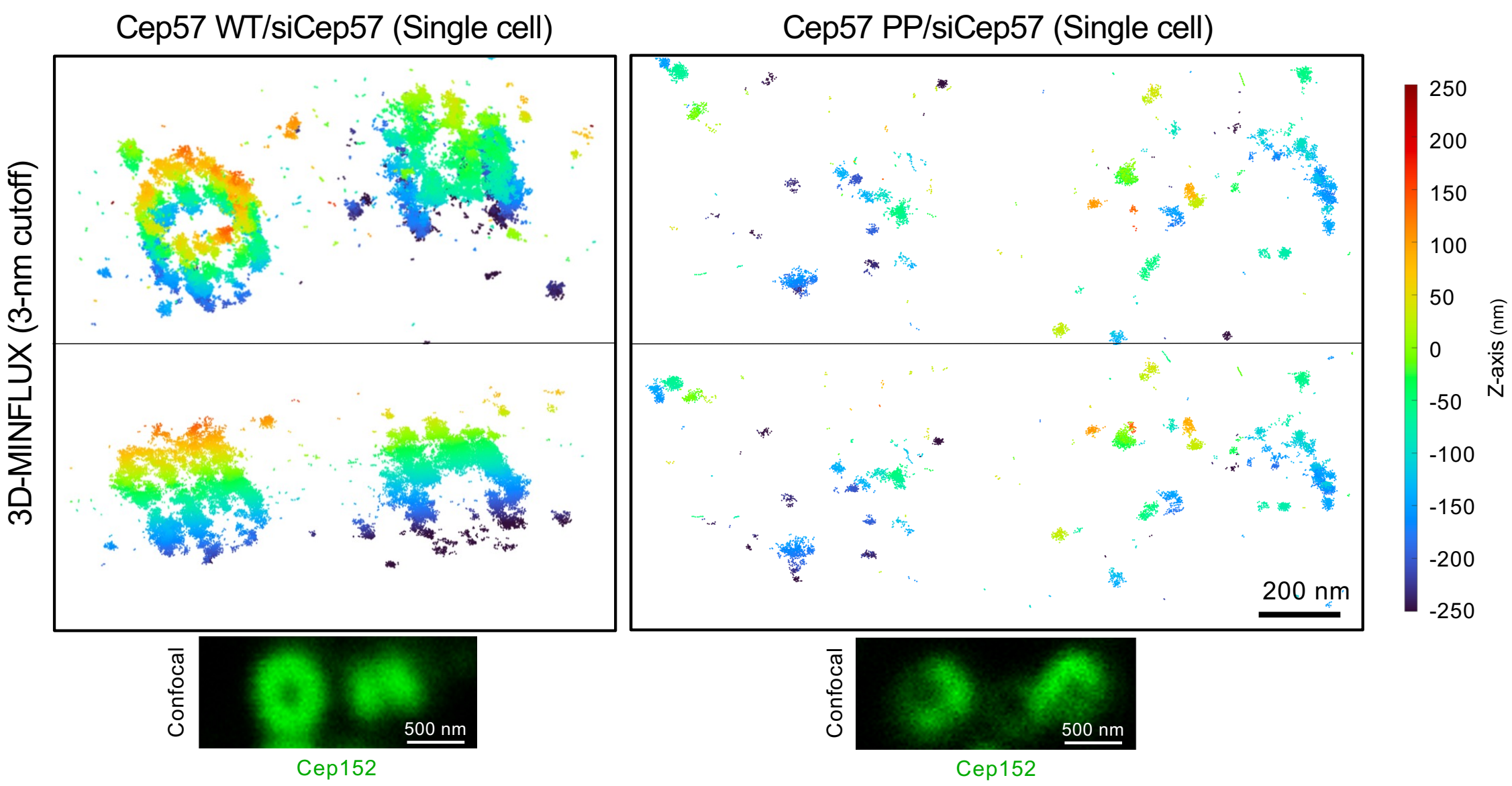

g

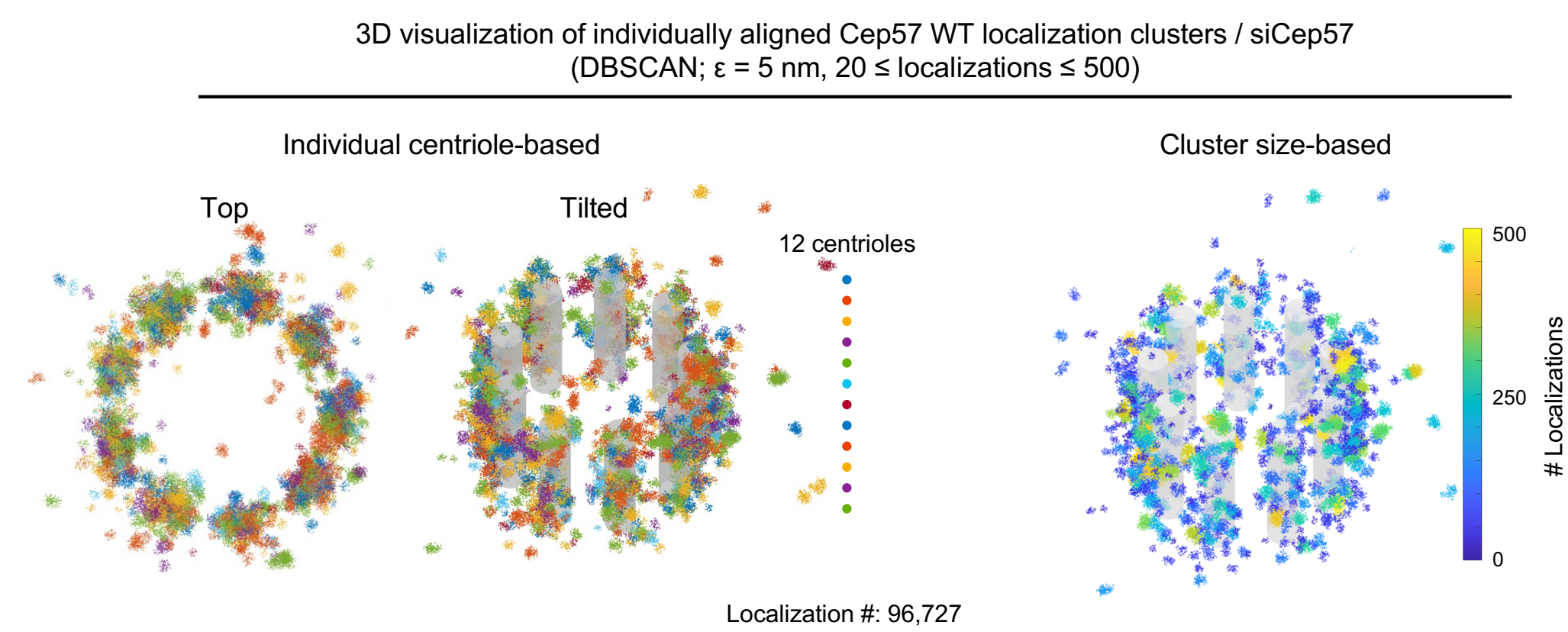

h

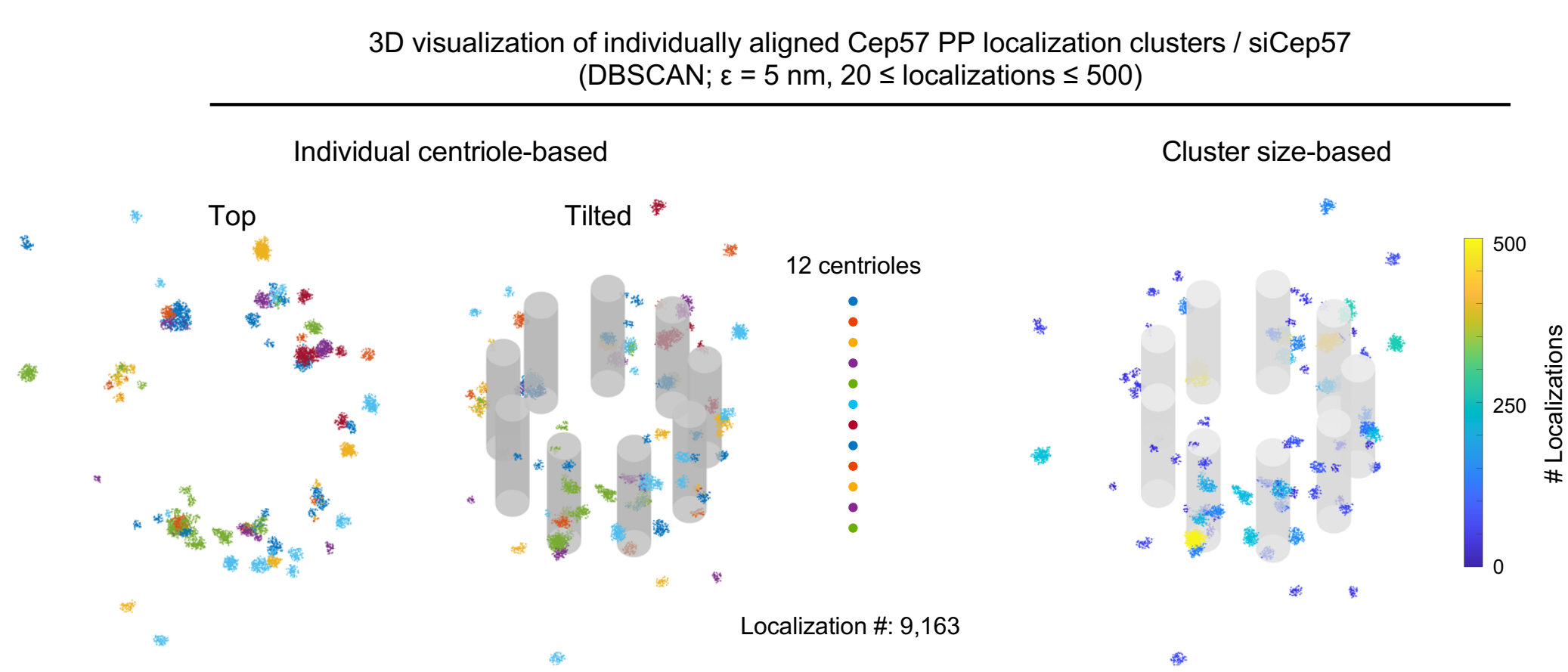

i

Large-cluster analysis

	Cep57 WT (n = 12 centrioles)	Cep57 PP (n = 24 centrioles)
Total localizations (3-nm cutoff)	223,038*	45,524*
Total large-cluster localizations (ε = 5 nm, 50 ≤ localizations ≤ 500)	30,200	4,798
Clustered localizations/centriole	2,517	200

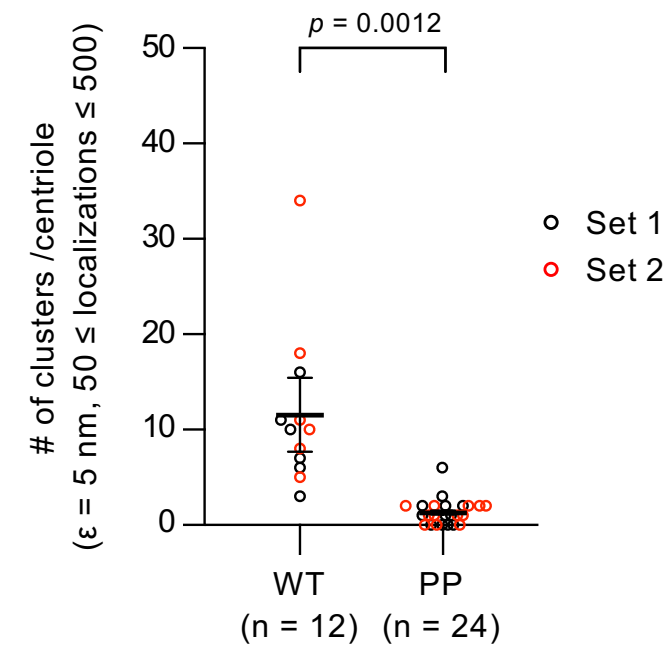

j

Large-cluster analysis

(ε = 5 nm, 50 ≤ localizations ≤ 500; **5-localization cutoff**)

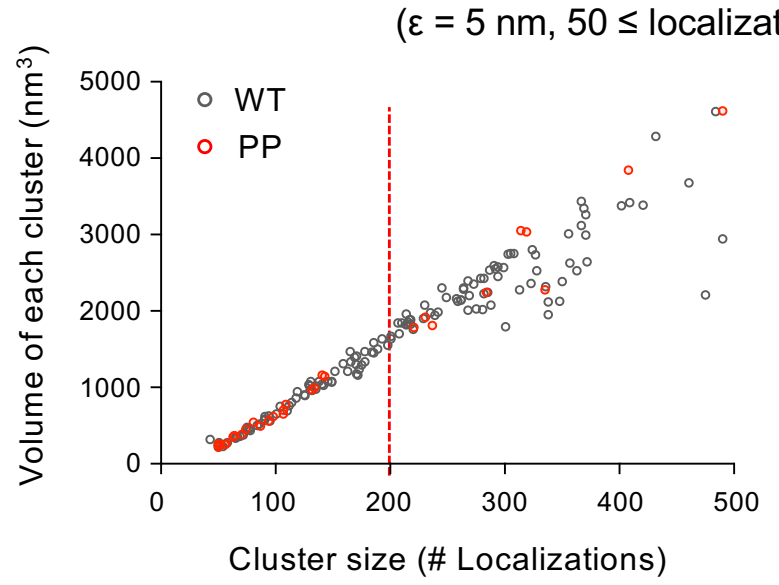

Distribution of clustered localizations	≤ 200	> 200
Cep57 WT (n = 12 centrioles)	46.8%	53.2%
Cep57 PP (n = 24 centrioles)	71.0%	29.0%

Supplementary Fig. 3. Characterization of the V307P M314P mutant by biochemical and cell-based analyses and MINFLUX nanoscopy. **(a)** Circular dichroism (CD) analyses showing the V307P M314P (PP) mutations largely crippled the alpha helicity observed in its parental WT sequence. Three replicate measurements of absorbance and ellipticity for WT and its PP mutant were plotted (**a**, left and middle). Proteins used for the CD measurement are shown after Coomassie Brilliant Blue staining (**a**, right). **(b)** SEC elution profiles of the indicated proteins analyzed with the Superdex 200 Increase 10/300 GL column. Arrows on the gel, MBP-Cep57 (277–334) and its PP mutant injected into the column. **(c)** iSCAMS analyses showing the concentration-dependent oligomerization of MBP-Cep57 (277–334) but not its respective PP mutant. Proteins shown in **(b)** were used. **(d** and **e)** Immunolocalization analyses for U2OS cells stably expressing Flag-Cep57 or its respective PP mutant using the cells in Fig. 3e (immunoblot). Representative confocal images are shown. Quantified data for Flag and Cep152 signals from (**d**) are provided in Fig. 3e, and those for SAS6 from (**e**) are provided in Fig. 3f. Quantified data for CP110 signals shown in (**e**, bottom) were obtained from three independent experiments [per experiment: under siGL; n ≥ 209 (total n = 719) for vector, and under siCep57; n ≥ 215 (total n = 746) for vector, n ≥ 205 (total n = 725) for WT, n ≥ 216 (total n = 701) for PP]. Bars, means of three experiments ± s.d.; *P* values, unpaired two-tailed *t*-tests. Boxes, areas of enlargement. **(f)** Two representative 3D-MINFLUX images (shown at two different angles) displaying the localizations of stably expressed Cep57 WT and its oligomerization-defective PP mutant in U2OS cells depleted of endogenous Cep57 (siCep57) and immunostained with an anti-Cep57 antibody and Alexa Fluor 647–conjugated anti-rabbit antibody. The confocal images (bottom) are anti-Cep152 signals marking the centrioles used for the Cep57 MINFLUX imaging above. **(g** and **h)** Twelve representative 3D-MINFLUX localization datasets (for each Cep57 WT and the PP mutant) acquired from the same samples used for (**f**) were analyzed by filtering out noise signals with a 3-nm cutoff and DBSCAN (ε = 5 nm, localizations ≥ 20) (left). The 3D localization maps of Cep57 WT and the PP mutant (right) were generated by combining individually DBSCANed localization datasets and aligning them using MC and ICP algorithms. Clusters are color-coded based on the number of localizations per cluster (20-to-500 localizations). **(i)** Summarized data showing the total number of localizations obtained after a 3-nm cutoff and a DBSCAN filtering (ε = 5 nm, 50 ≤ localizations ≤ 500) to quantify large clusters of Cep57 WT and the PP mutant in U2OS cells silenced for endogenous Cep57 by RNAi. Asterisks indicate the total localizations with a 3-nm cutoff obtained from the two independent datasets: 6 mother centrioles/set (total 12 for WT) or 12 mother centrioles/set (total 24 for PP). Because of the low number of localizations for the PP mutant, the number of centrioles analyzed was increased to 24. Signal clusters generated from these 3-nm cutoff localizations are provided in Fig. 3h. **(j)** The distribution pattern of large clusters (ε = 5 nm, 50 ≤ localizations ≤ 500) obtained after removal of traces shorter than 5-localizations (i.e., 5-localization cutoff) for the same raw localizations used for Fig. 3h and Supplementary Fig. 3i. The results are essentially the same as those obtained with the localization-precision-based (i.e., 3-nm cutoff) filtering in Fig. 3h.

Ahn, J., et al. Supplementary Fig. 4

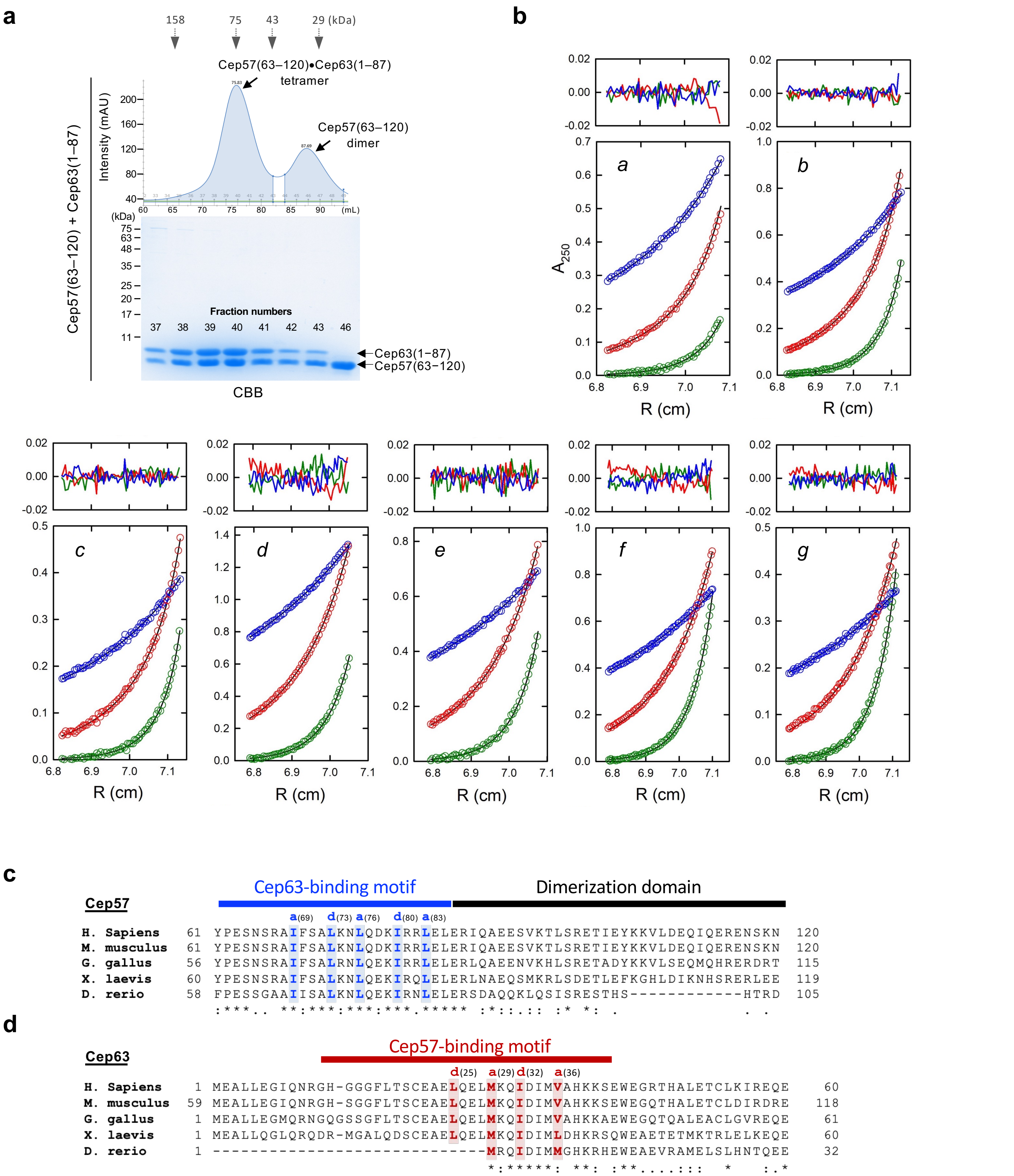

Ahn, J., et al. Supplementary Fig. 4

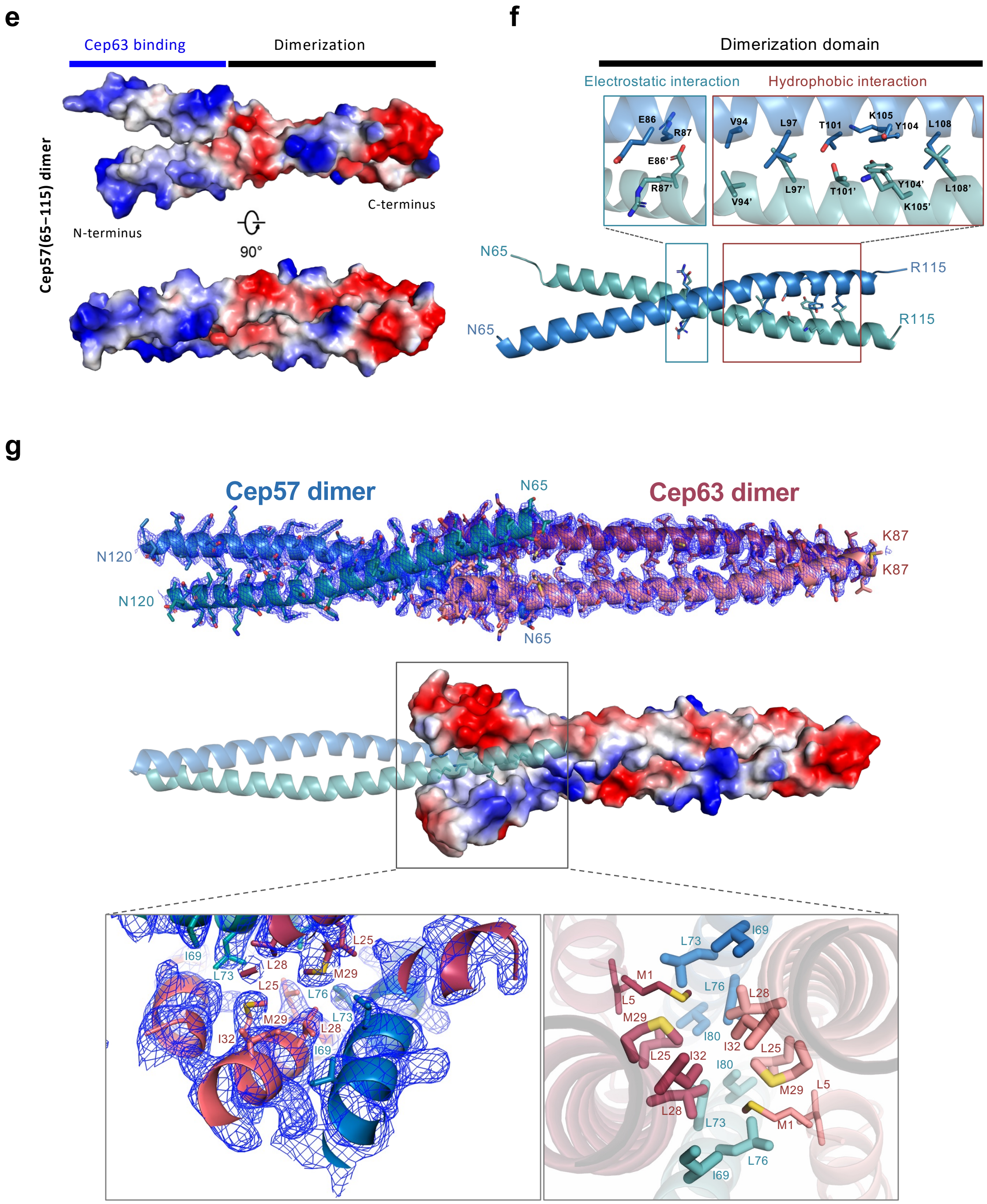

h

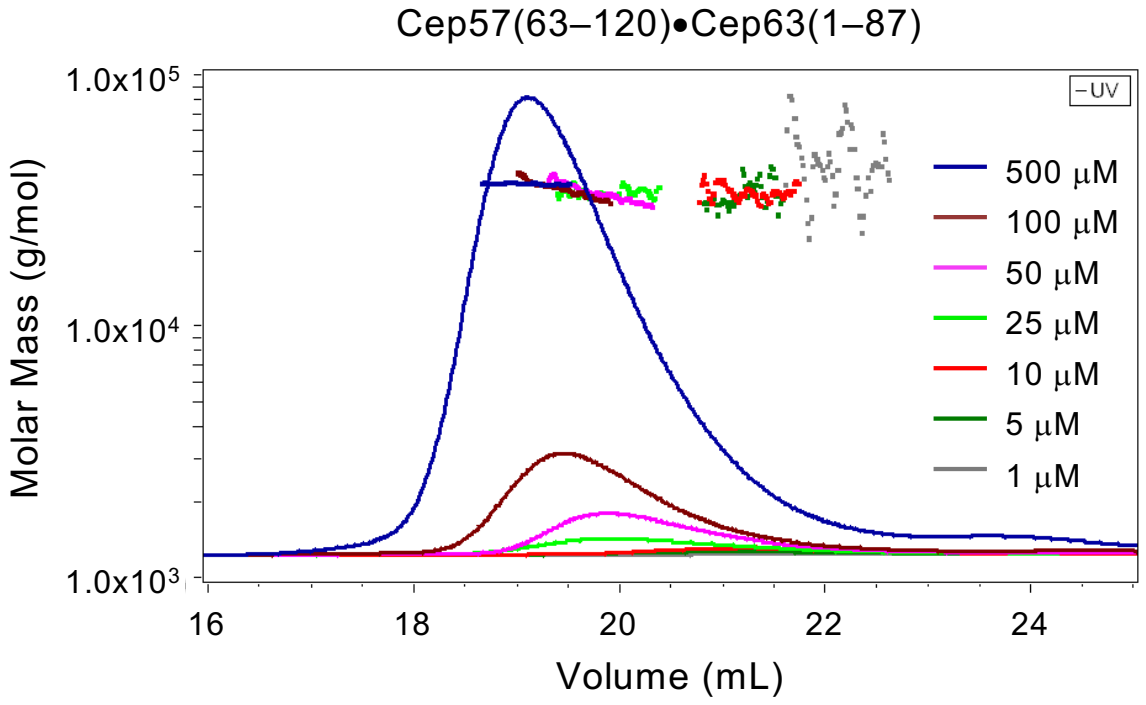

Conc. (μM)	MW (kDa)
500	36.9 (±1.5%)
100	34.9 (±2.0%)
50	33.9 (±2.0%)
25	34.1 (±3.2%)
10	34.1 (±15.2%)
5	33.4 (±11.0%)
1	47.1 (±24.0%)

i

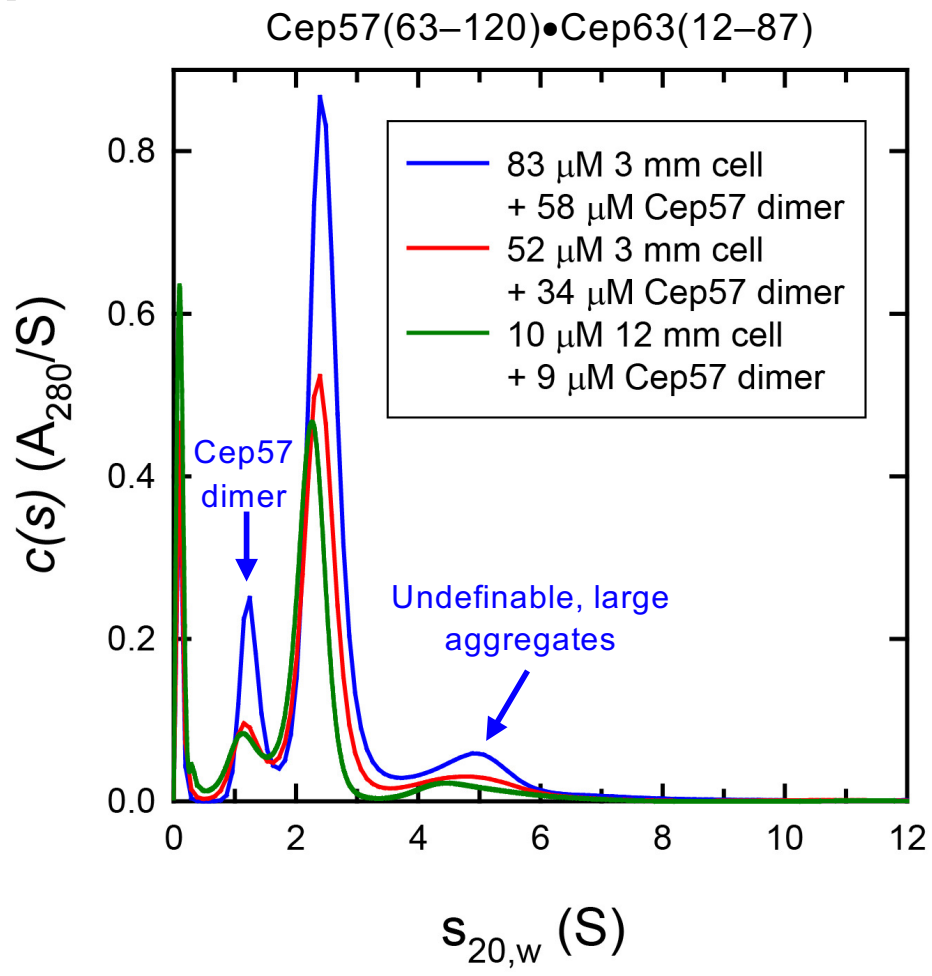

Supplementary Fig. 4. Biochemical, biophysical, and structural analyses of the Cep57(65–115) dimeric and the heterotetrameric Cep57(63–120)•Cep63(1–87) complexes. **(a)** A SEC elution profile and an SDS-PAGE gel showing the heterotetrameric Cep57(63–120)•Cep63(1–87) and dimeric Cep57(63–120) complexes. CBB, Coomassie Brilliant Blue-stained gel. **(b)** Sedimentation equilibrium analyses showing co-operativity in forming a hexadecameric Cep57-Cep63 complex. Absorbance sedimentation equilibrium data were collected at 8,000 (green), 14,000 (red), and 22,000 (blue) rpm and various concentrations as indicated. (*a*) 275 μM, 3 mm cell at 250 nm, (*b*) 145 μM, 3 mm cell at 280 nm, (*c*) 145 μM, 3 mm cell at 250 nm (*d*) 70 μM, 12 mm cell at 280 nm, (*e*) 70 μM, 12 mm cell at 250 nm, (*f*) 35 μM, 12 mm cell at 280 nm, and (*g*) 35 μM, 12 mm cell at 250 nm. Data were analyzed globally using mass conservation as a constraint using a monomer–dimer–tetramer self-association model in which the monomer represents the heterotetrameric Cep57-Cep63 complex, the dimer an octameric Cep57-Cep63, and the tetramer a hexadecameric Cep57-Cep63. Solid lines show the best fits, and residuals are displayed in the corresponding plots above the sedimentation equilibrium data. For clarity, only every third data point is displayed. **(c and d)** Sequence alignment for Cep57 and Cep63 orthologs assembled using the Clustal Omega software. The primary amino-acid sequences of Cep152 orthologs used for the analysis are: *H. Sapiens* (NP_055494.2), *M. Musculus* (NP_080941.3), *G. gallus* (XP_417188.2), *X. laevis* (XP_018105836.1), and *D. Rerio* (NP_001074152.1). The Cep63-binding motif and the dimerization domain on human Cep57 and the Cep57-binding motif on human Cep63 are noted. The predicted **a** and **d** positions of the helical wheel are marked on Cep57 and Cep63 sequences. Asterisk (*), fully conserved residues across all sequences in the alignment; colon (:), moderately conserved residues; dot (.), not conserved but similar residues. **(e–g)** Structural models showing the Cep57(65–115) dimeric and the Cep57(63–120)•Cep63(1–87) heterotetrameric complex. The electron density (2*Fo–Fc*) maps shown in (**g**) were contoured at 1.0 σ. **(h)** SEC-MALS profiles showing the stability of the Cep57(63–120)•Cep63(1–87) complex under various diluted conditions were obtained using the Superdex 75 10/300 GL column (injection volume, 200 uL; running buffer: 20 mM HEPES pH 7.5, 50 mM NaCl, 4 mM 2-mercaptoethanol). (**i**) Absorbance sedimentation c(*s*) profiles for the N-terminal residues (1–11)-deleted Cep57(63–120)•Cep63(12–87) complex as a function of concentration, showing evidence of forming concentration-dependent (modestly), undefinable, large aggregates (a broader peak of 4 S–6 S ; arrow), when compared to that of the parental Cep57(63–120)•Cep63(1–87) complex in Fig. 4c. The Cep57 dimer, which could not be removed during purification and therefore contributed to the analysis, is marked. The octameric form of the complex is expected to sediment at 3.6 S. These data suggest that loss of the Cep63 (1–11) motif disrupts thermodynamic equilibrium, likely by exposing cryptic interfaces and thereby preventing the stepwise assembly of higher MW complexes.

Ahn, J., et al. Supplementary Fig. 5

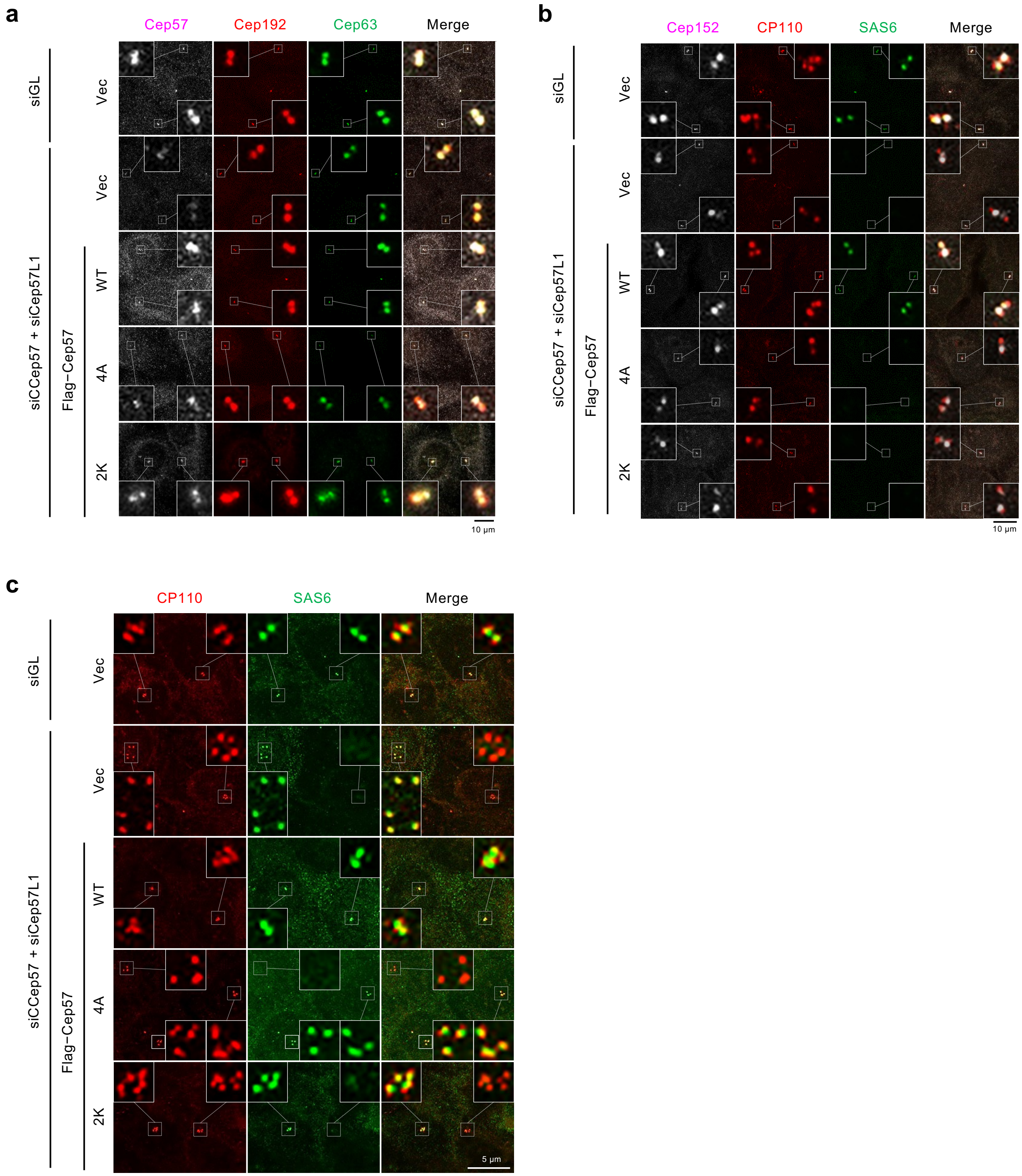

Ahn, J., et al. Supplementary Fig. 5

d

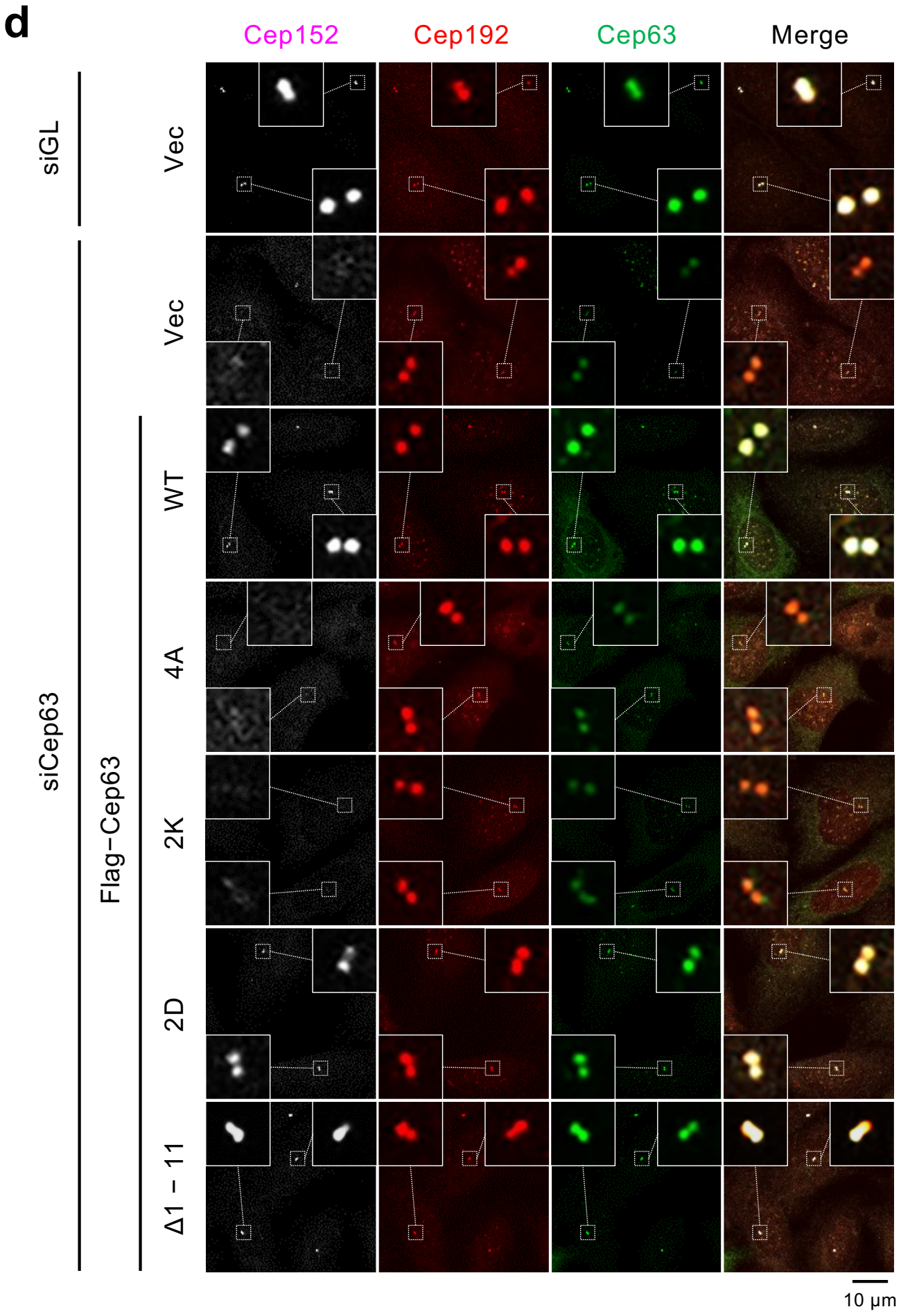

e

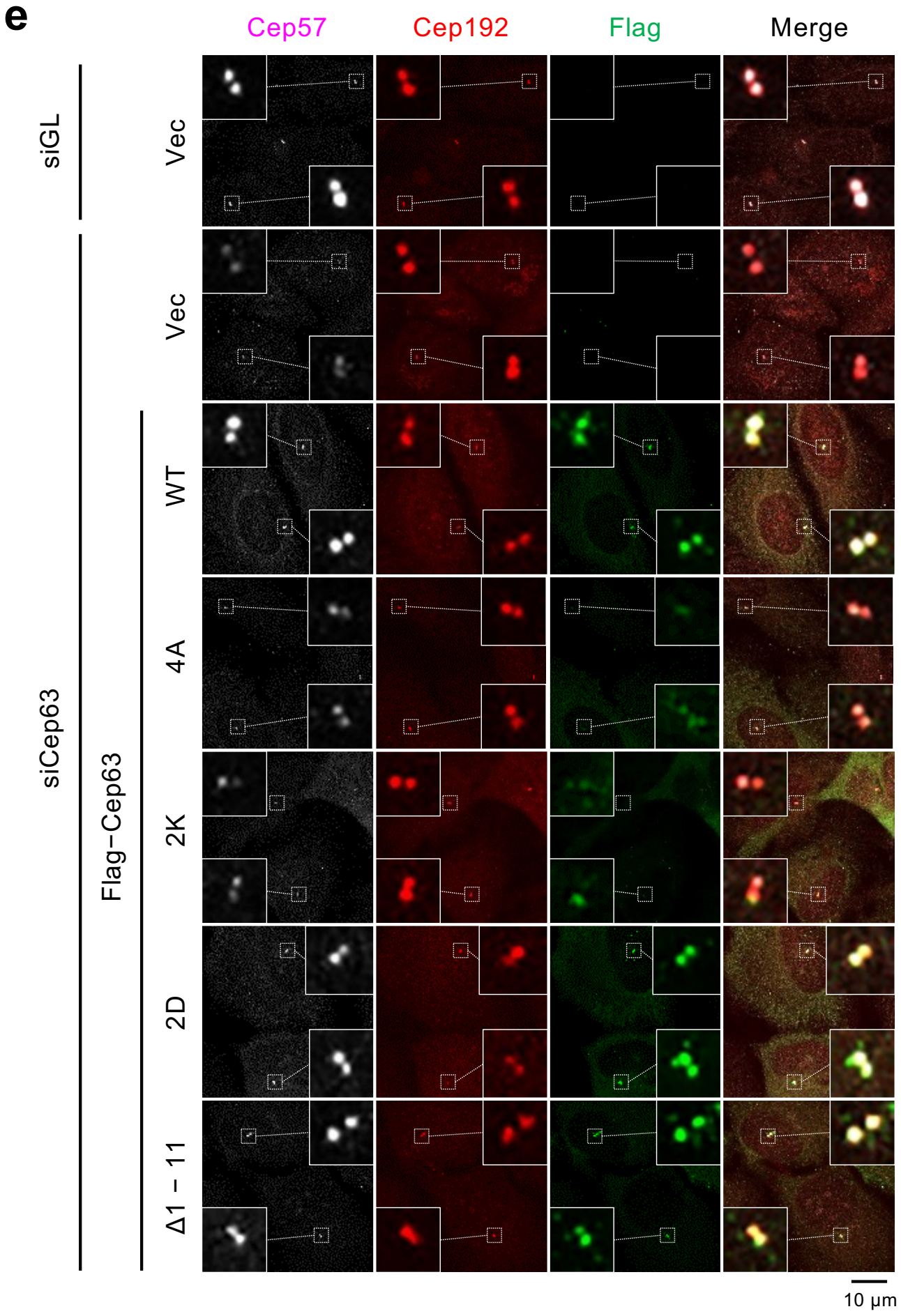

f

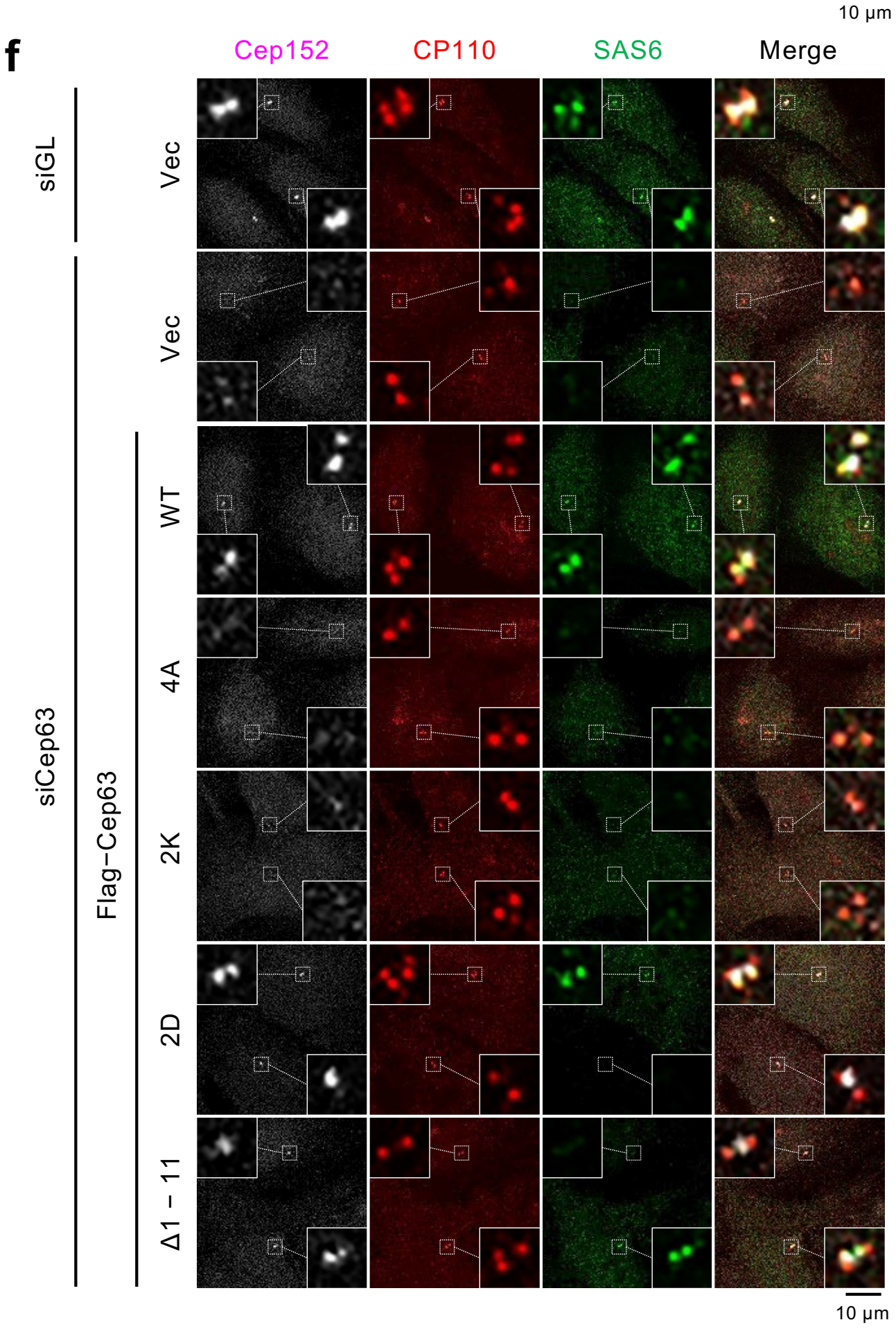

Supplementary Fig. 5. Confocal images showing abnormalities in centriole numbers in cells expressing Cep57 or Cep63 mutants impaired in the Cep57–Cep63 interaction. **(a–c)** Representative confocal images of immunostained U2OS cells stably expressing the indicated Flag-Cep57 constructs under the Cep57 and Cep57L1 RNAi conditions (siCep57 + siCep57L1). Quantified data obtained from these analyses are provided in Fig. 6b,c. Note that the panels in (**c**) show Sas6 and CP110 signals consistent with the extensive centriole disengagement. Boxes, areas of enlargement. **(d–f)** Representative confocal images of immunostained U2OS cells stably expressing the indicated Flag-Cep63 constructs under the Cep63 RNAi condition (siCep63). Analyses were carried out similarly to those in (**a–c**). Quantified data are provided in Fig. 6e,f. Note that, unlike the Cep57 4A or 2K mutant in the siCep57 + siCep57L1 cells in (**a–c**), premature centriole disengagement is not apparent in these cells. Boxes, areas of enlargement.

Ahn, J., et al. Supplementary Fig. 6

a

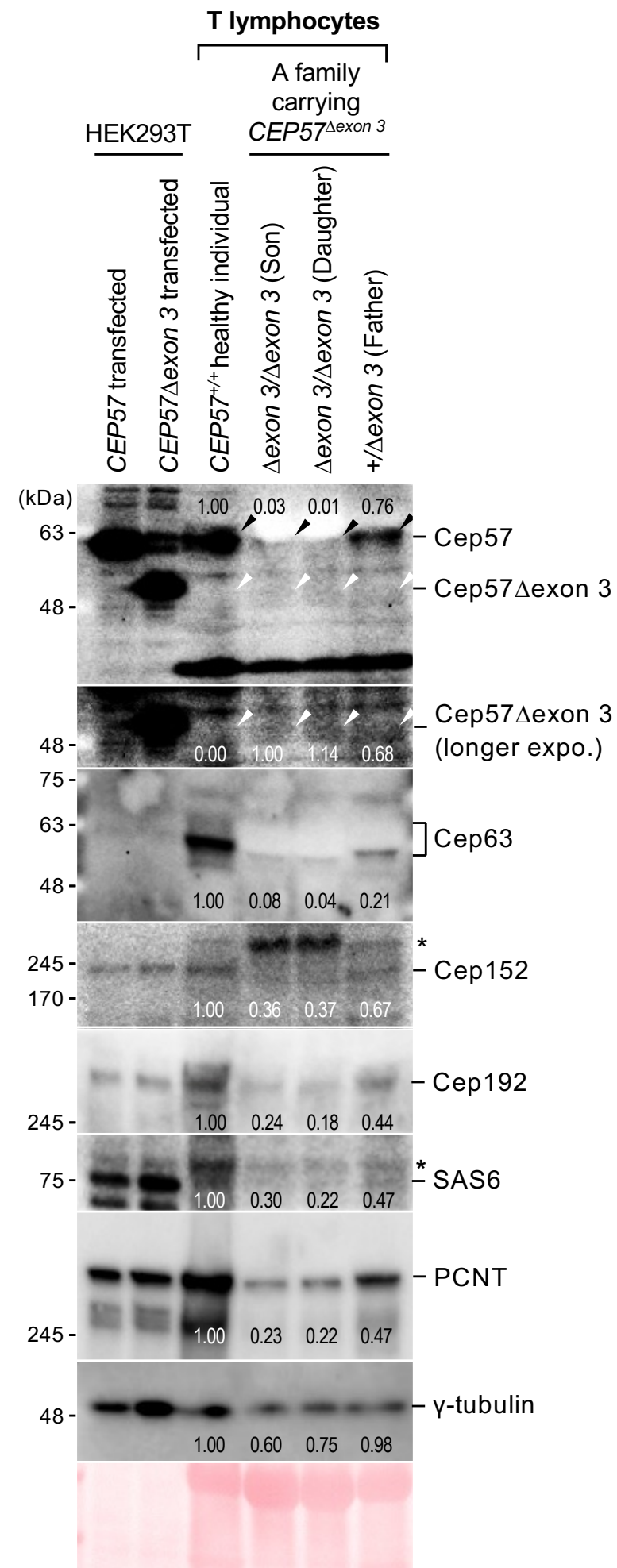

Ponceau S

Inner PCM scaffolds — outer PCM scaffolds

	Cep57	Cep63	Cep152	Cep192	SAS6	PCNT	γ-tubulin	Healthy
Son	0.033	0.082	0.357	0.240	0.302	0.231	0.595	1.000
Daughter	0.014	0.035	0.373	0.182	0.217	0.224	0.753	1.000
Father	0.764	0.210	0.666	0.441	0.470	0.468	0.980	1.000

0 — 0.5 — 1

Total protein levels

b

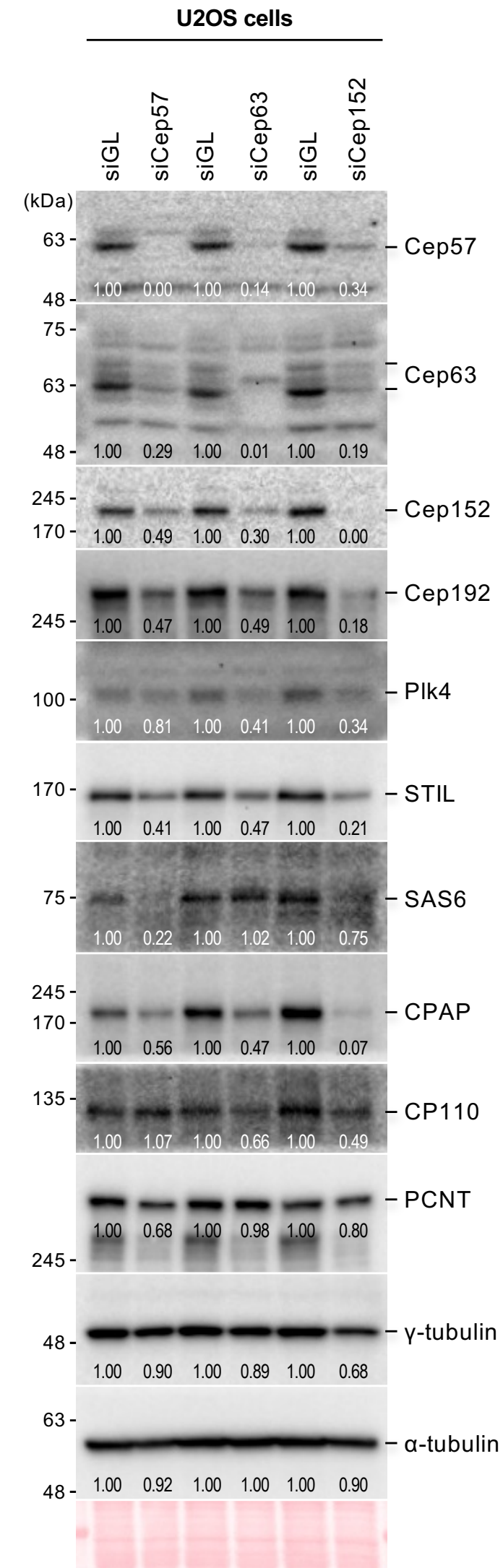

Ponceau S

Inner PCM scaffolds — Procentriole and cartwheel proteins — Distal end and outer PCM scaffolds

	Cep57	Cep63	Cep152	Cep192	Plk4	STIL	Sas6	CPAP	CP110	PCNT	γ-tubulin	α-tubulin	siGL
siCep57	0.002	0.290	0.493	0.471	0.808	0.411	0.217	0.555	1.073	0.683	0.899	0.916	1.000
siCep63	0.135	0.010	0.303	0.487	0.414	0.465	1.021	0.474	0.655	0.984	0.890	0.996	1.000
siCep152	0.337	0.193	0.002	0.181	0.342	0.206	0.749	0.065	0.487	0.796	0.683	0.895	1.000

0 — 0.5 — 1

Total protein levels

c

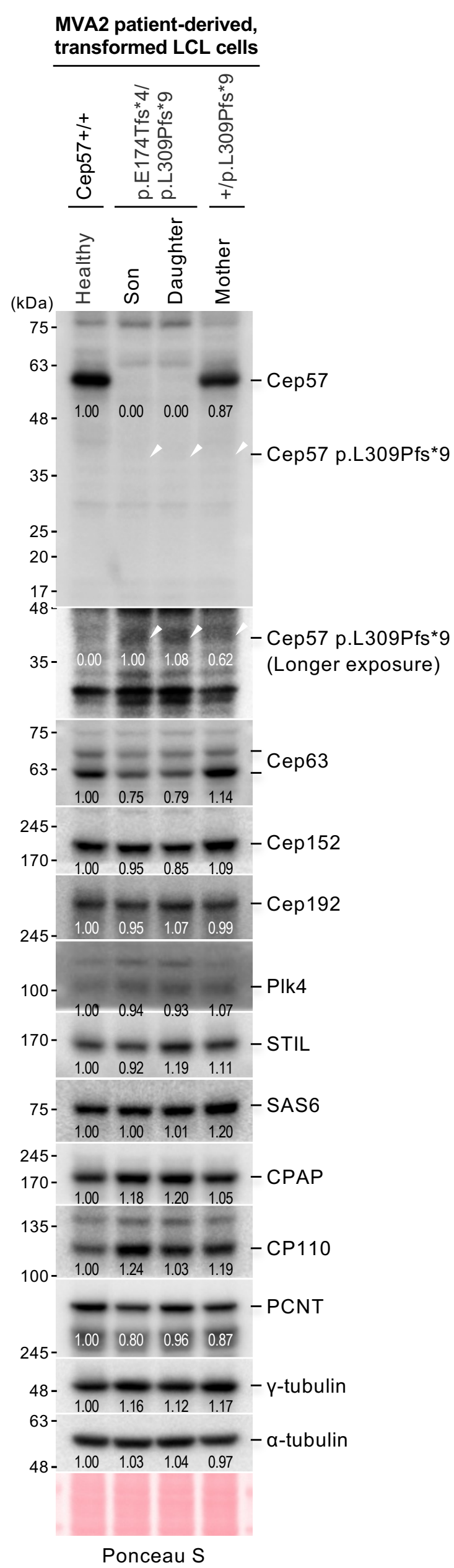

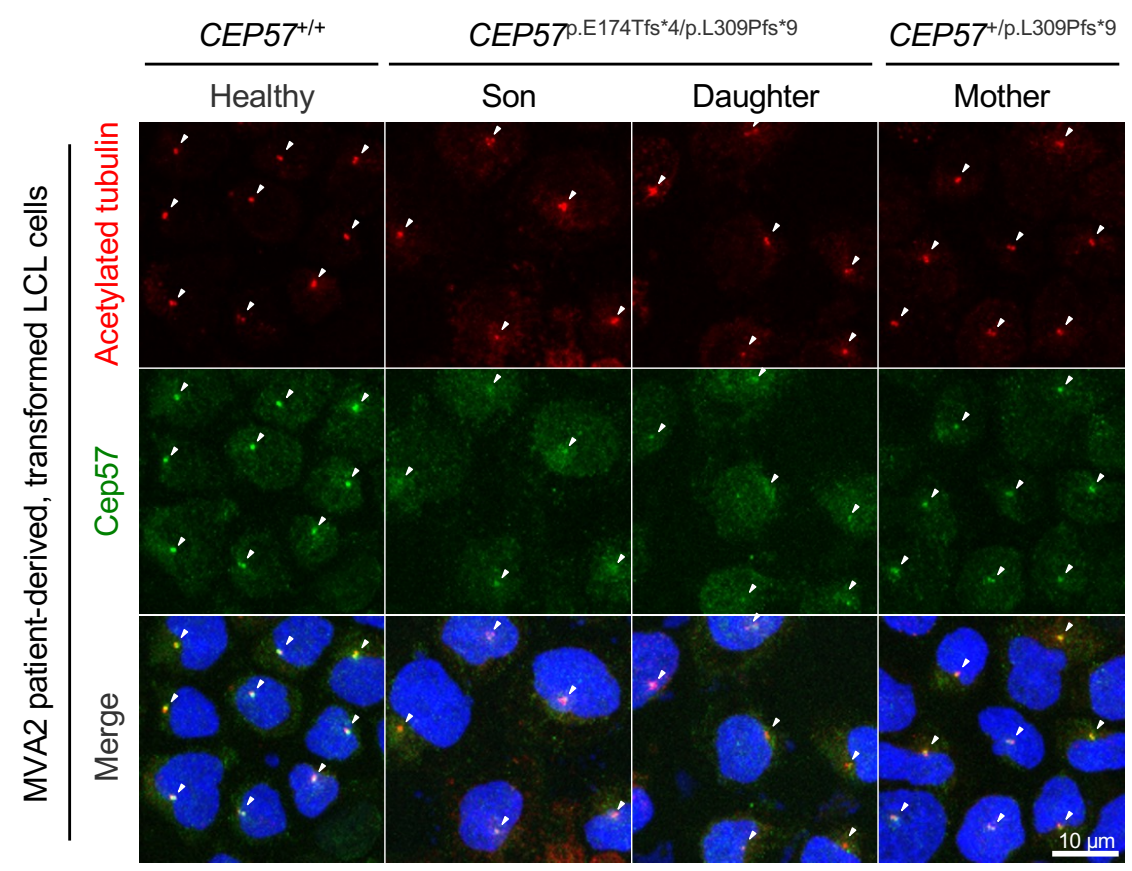

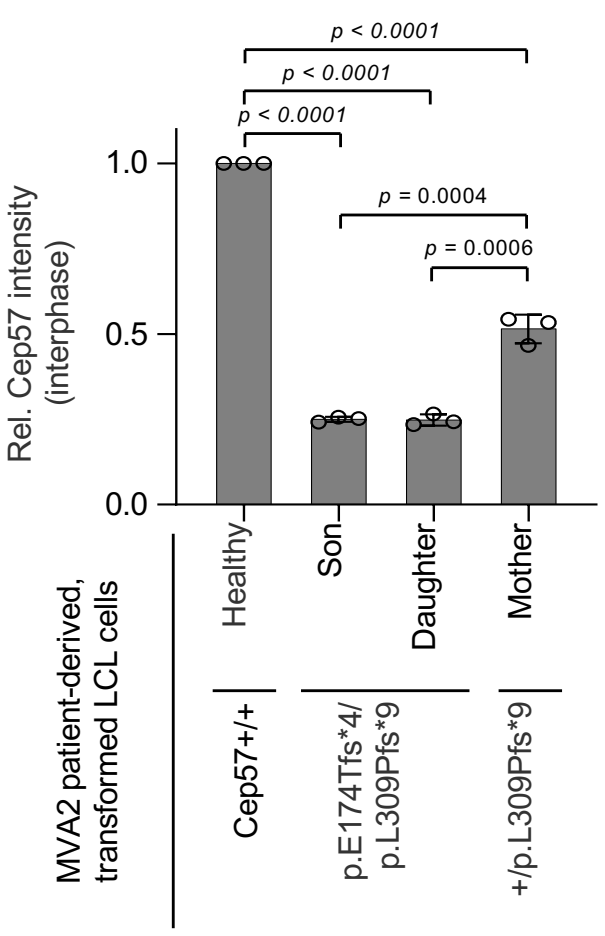

Inner PCM scaffolds | Procentriole and cartwheel proteins | Distal end and outer PCM scaffolds

	Cep57	Cep63	Cep152	Cep192	Plk4	STIL	SAS6	CPAP	CP110	PCNT	γ-tubulin	α-tubulin	Healthy
Son	0.005	0.749	0.950	0.951	0.936	0.921	1.001	1.183	1.238	0.804	1.164	1.027	1.000
Daughter	0.003	0.786	0.848	1.072	0.933	1.193	1.011	1.198	1.028	0.956	1.121	1.043	1.000
Mother	0.874	1.135	1.091	0.990	1.073	1.113	1.195	1.054	1.192	0.872	1.172	0.970	1.000

0 0.5 1

Total protein levels

Ahn, J., et al. Supplementary Fig. 6

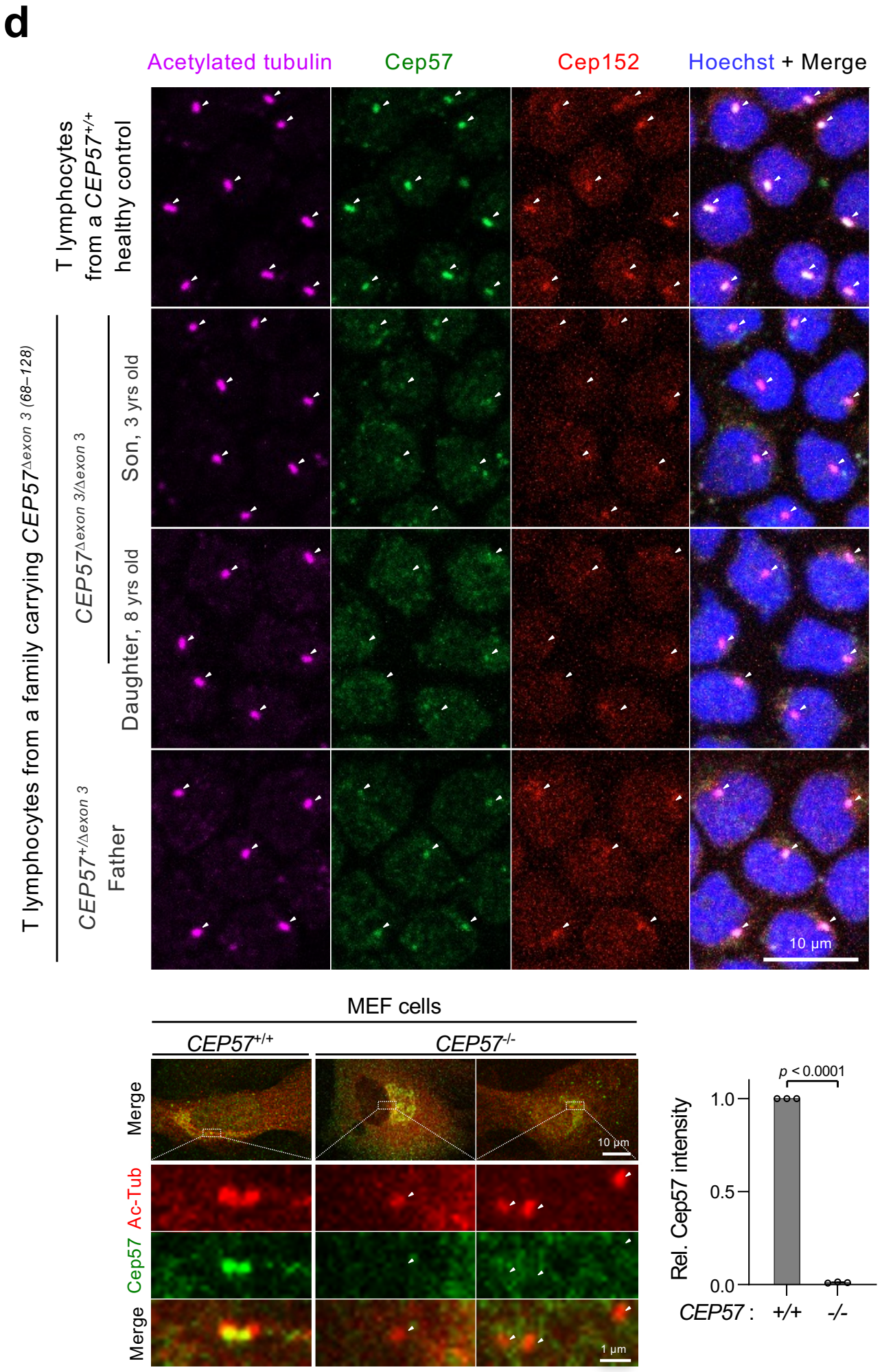

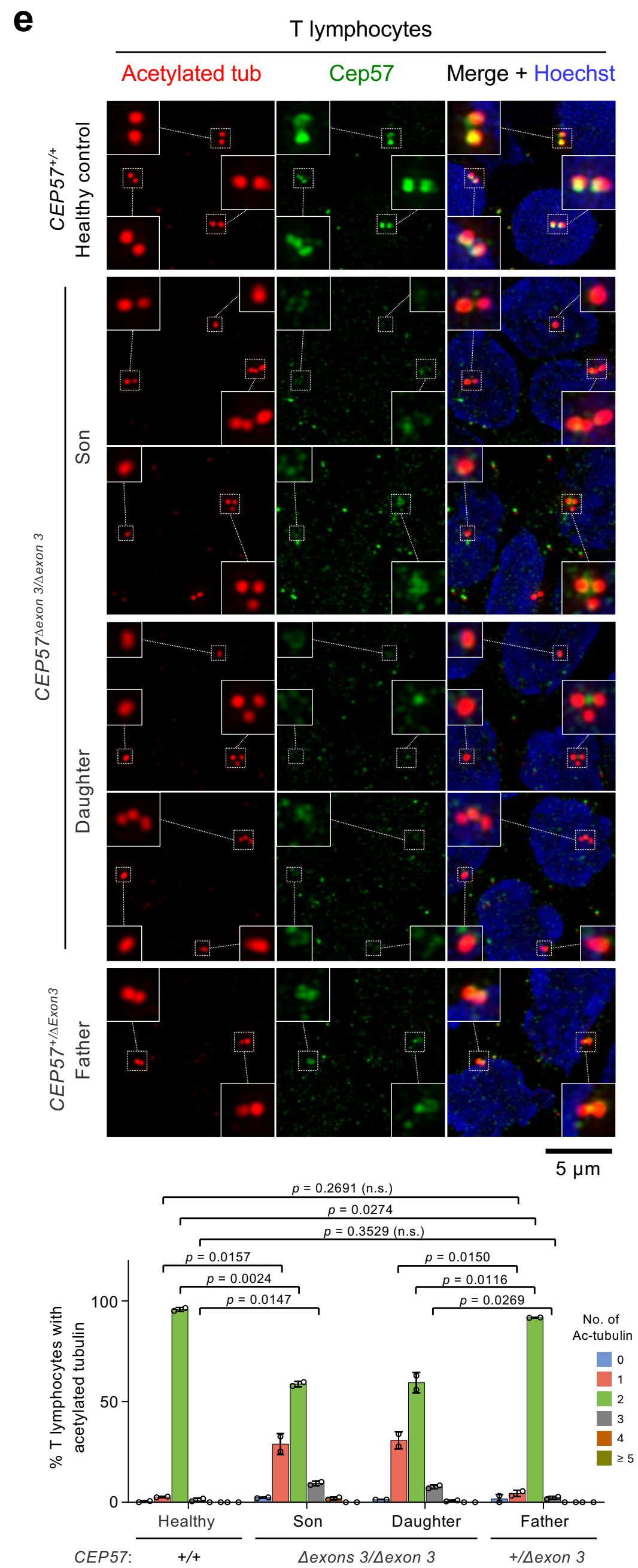

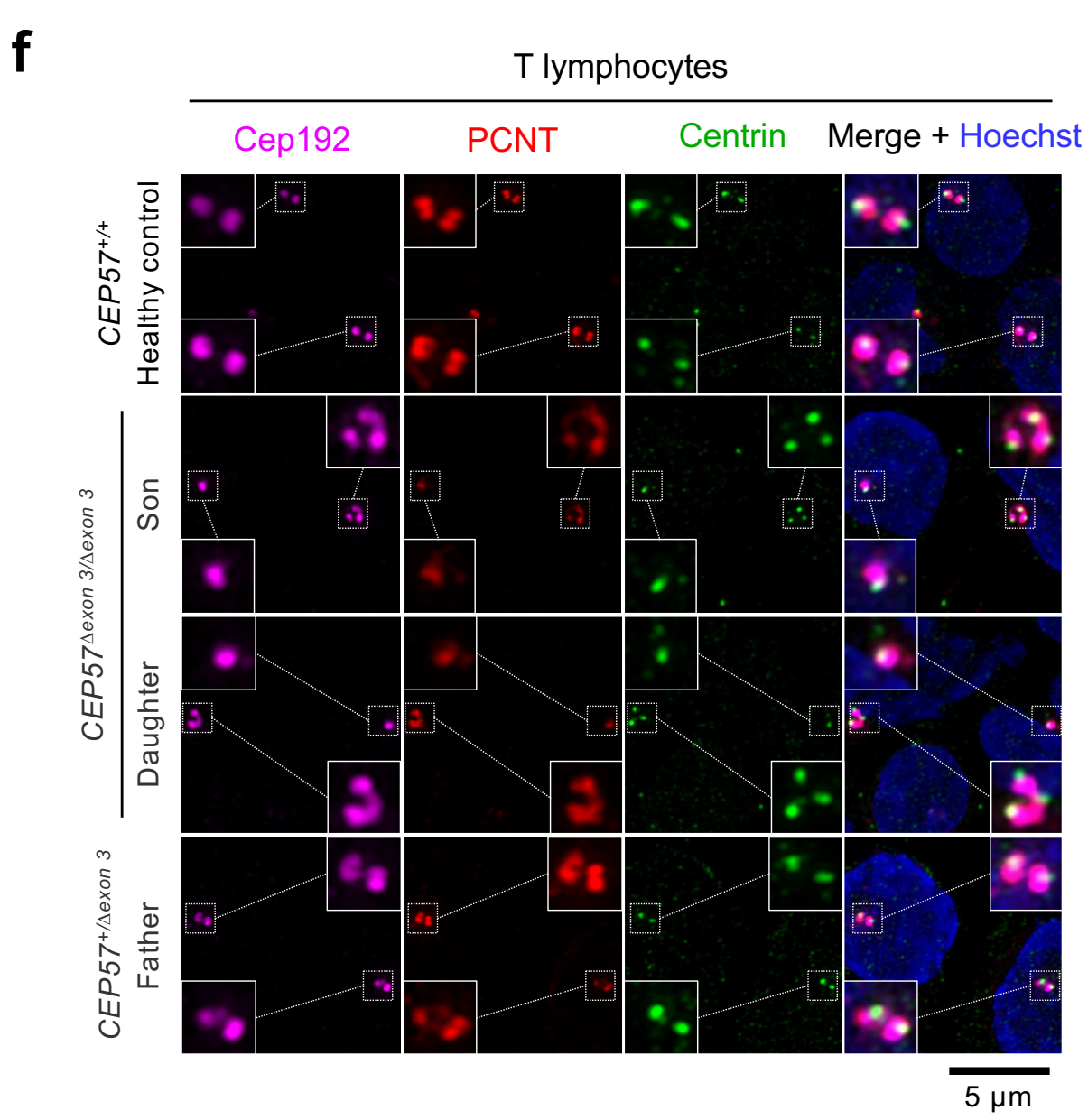

g

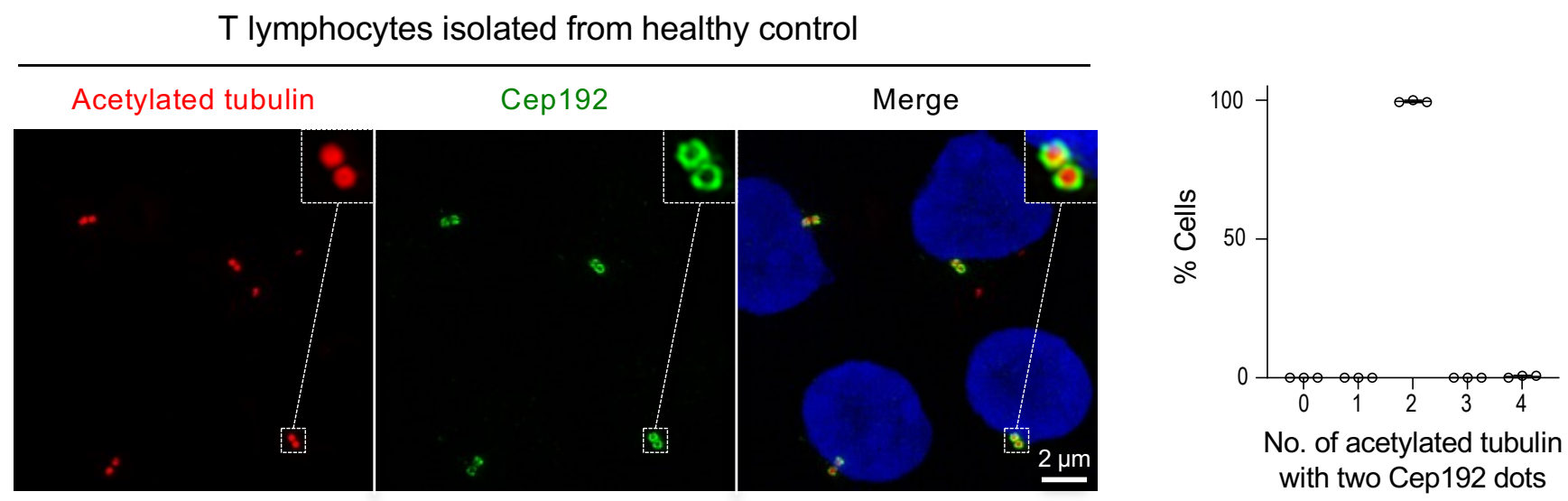

T lymphocytes isolated from healthy control
Acetylated tubulin
Cep192
Merge
2 μm
% Cells
100
50
0
0
1
2
3
4
No. of acetylated tubulin with two Cep192 dots

h

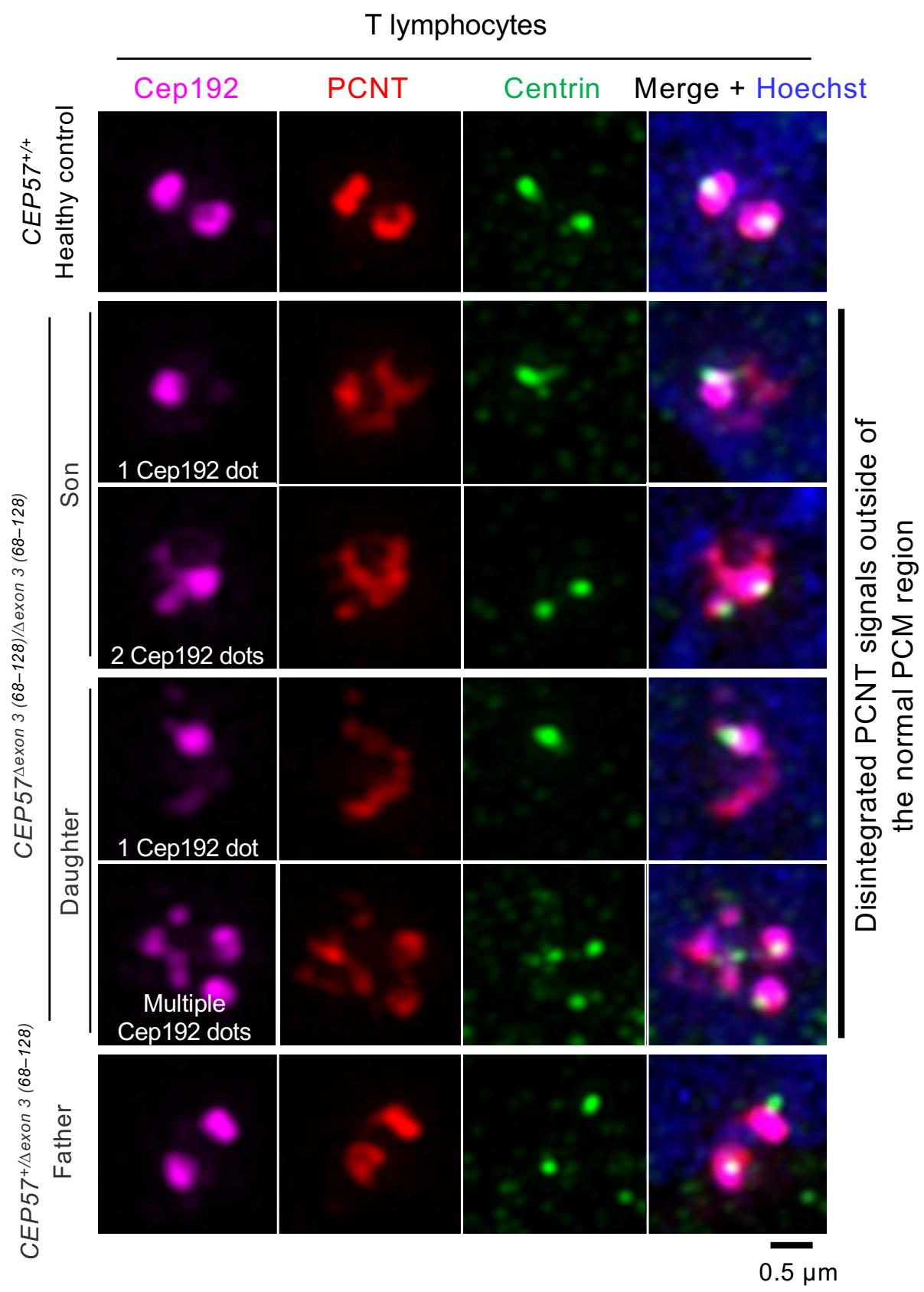

T lymphocytes
Cep192
PCNT
Centrin
Merge + Hoechst
CEP57+/+ Healthy control
CEP57Δexon 3 (68–128)/Δexon 3 (68–128)
Son
Daughter
1 Cep192 dot
2 Cep192 dots
1 Cep192 dot
Multiple Cep192 dots
CEP57+/Δexon 3 (68–128)
Father
0.5 μm
Disintegrated PCNT signals outside of the normal PCM region

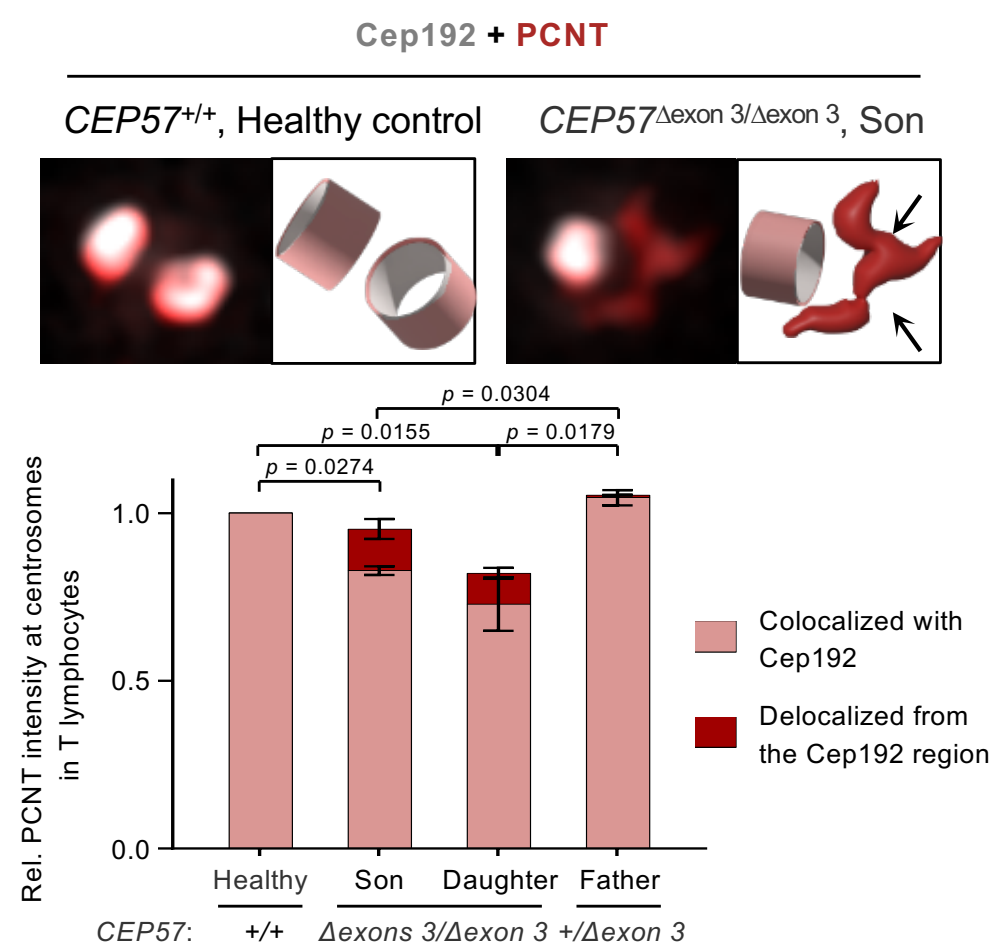

Cep192 + PCNT
CEP57+/+, Healthy control
CEP57Δexon 3/Δexon 3, Son
p = 0.0304
p = 0.0155
p = 0.0179
p = 0.0274
Rel. PCNT intensity at centrosomes in T lymphocytes
1.0
0.5
0.0
Colocalized with Cep192
Delocalized from the Cep192 region
Healthy
Son
Daughter
Father
CEP57:
+/+
Δexons 3/Δexon 3
+/Δexon 3

i

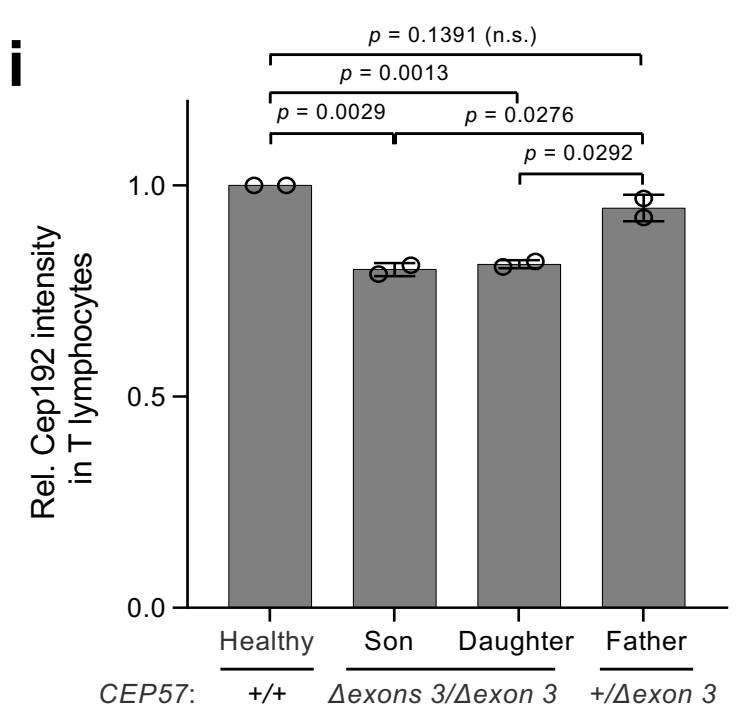

p = 0.1391 (n.s.)
p = 0.0013
p = 0.0029
p = 0.0276
p = 0.0292
Rel. Cep192 intensity in T lymphocytes
1.0
0.5
0.0
Healthy
Son
Daughter
Father
CEP57:
+/+
Δexons 3/Δexon 3
+/Δexon 3

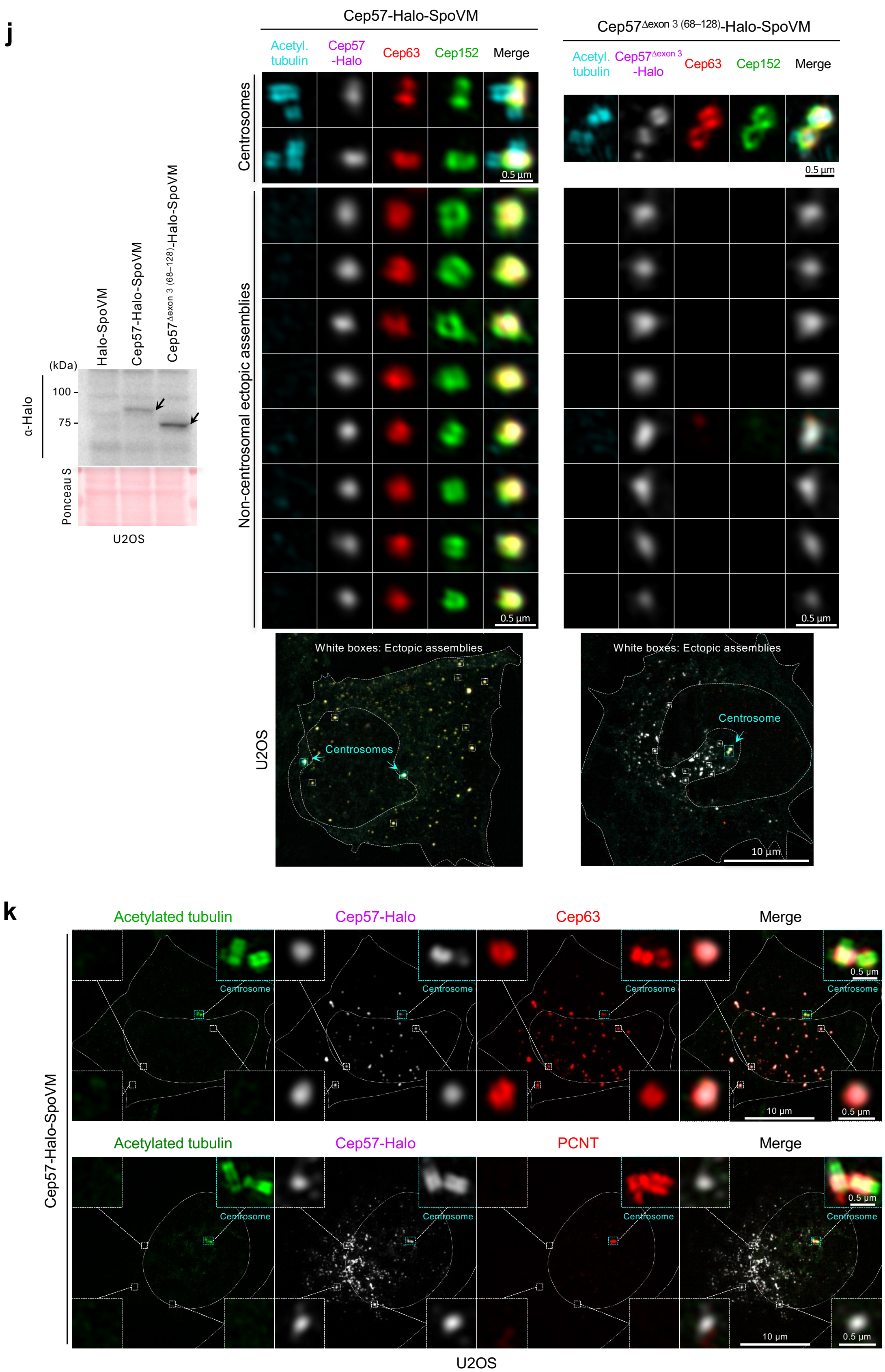
j
Cep57-Halo-SpoVM
Cep57Δexon 3 (68–128)-Halo-SpoVM
Acetyl. tubulin
Cep57 -Halo
Cep63
Cep152
Merge
Cep57Δexon 3 -Halo
Centrosomes
Non-centrosomal ectopic assemblies
0.5 µm
Halo-SpoVM
Cep57-Halo-SpoVM
Cep57Δexon 3 (68–128)-Halo-SpoVM
(kDa)
100
75
α-Halo
Ponceau S
U2OS
White boxes: Ectopic assemblies
Centrosomes
Centrosome
10 µm
k
Acetylated tubulin
Cep57-Halo
Cep63
PCNT
Merge
Centrosome
0.5 µm
10 µm
Cep57-Halo-SpoVM
U2OS

Ahn, J., et al. Supplementary Fig. 6

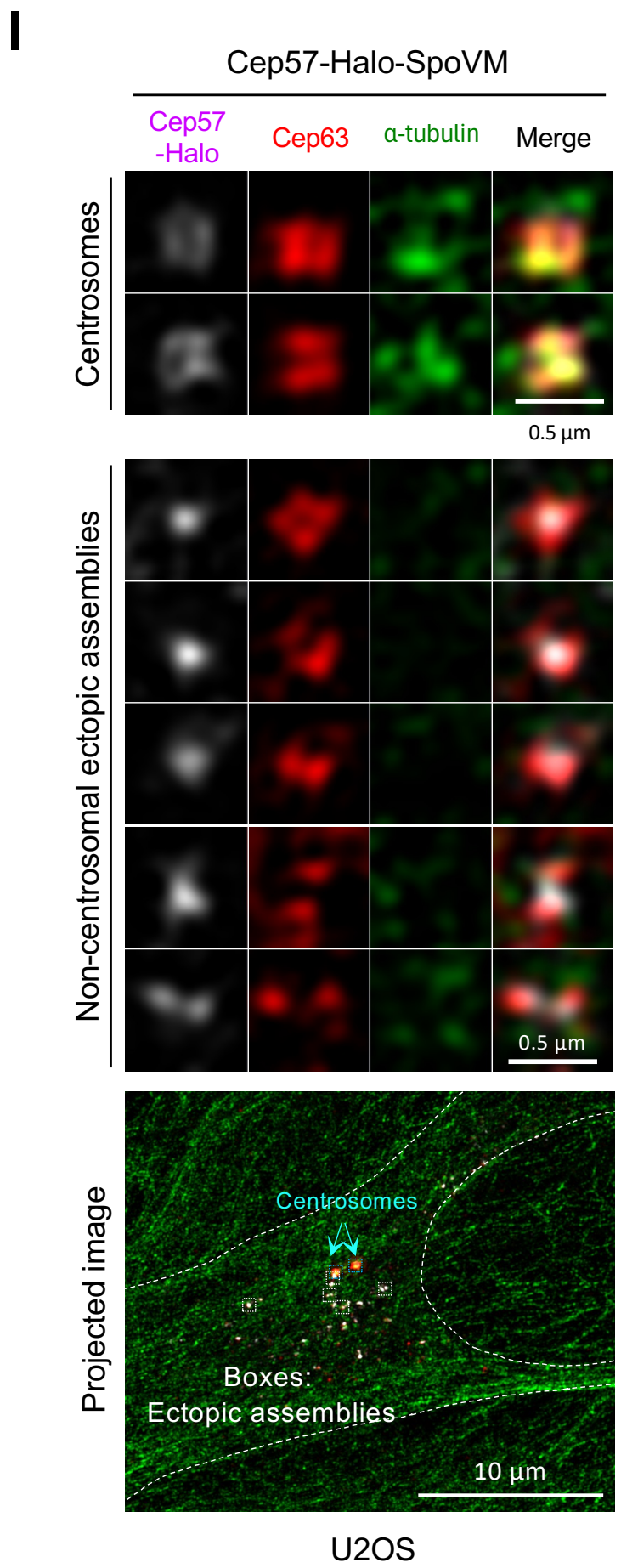

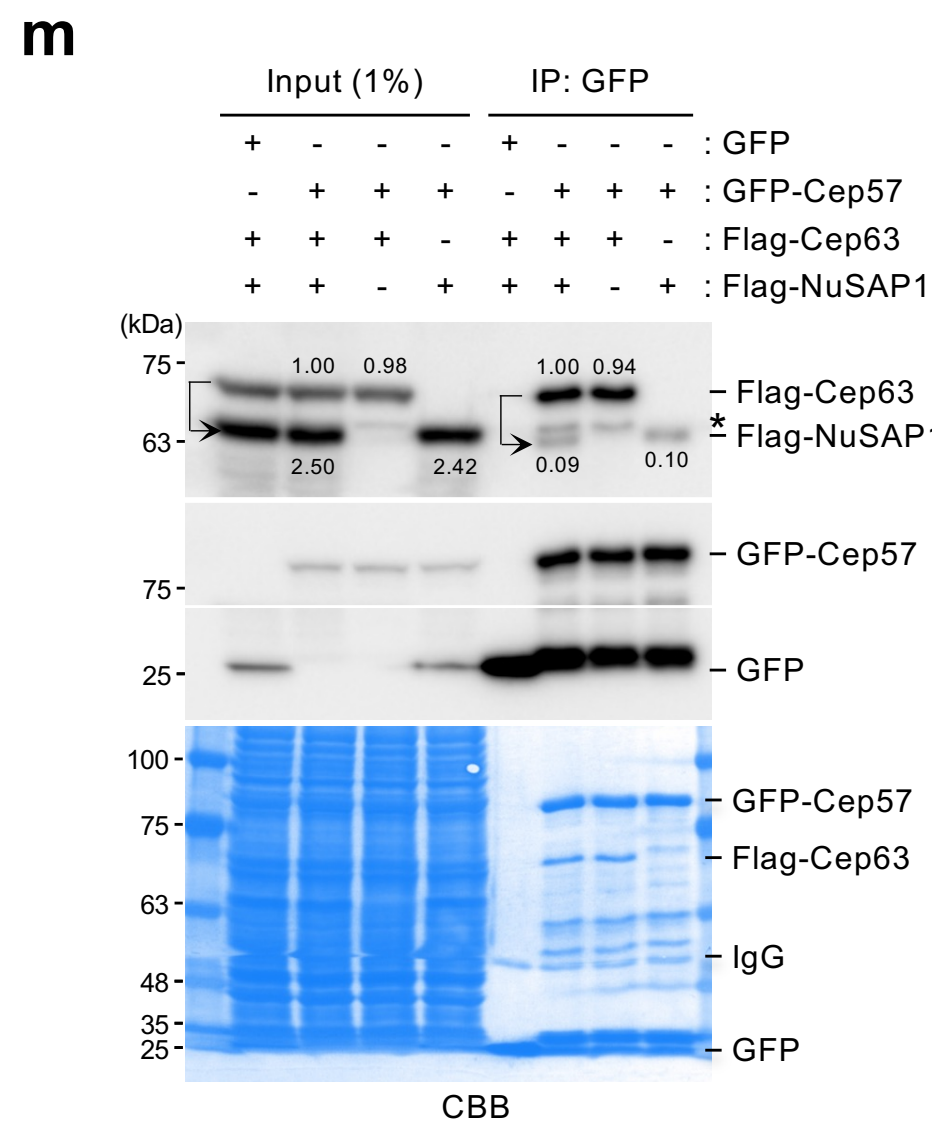

Supplementary Fig. 6. Loss of Cep57 exon 3 region-mediated interaction with Cep63 precludes normal centriole duplication by failing to establish Cep57–Cep63–Cep152 assemblies and undermining the stability of various inner PCM and centriolar proteins. **(a)** Immunoblotting analyses of the total lysates of the primary T cells purified from the indicated individuals. Arrowheads, the Cep57Δexon 3 mutant detected only in persons carrying the Δ*exon 3* mutation. Arrowheads indicate the respective protein signals. Asterisks, cross-reacting proteins. Numbers, relative signal intensities. The hit map (below) shows selective depletion of proteins at the inner PCM and procentrioles. **(b)** Immunoblotting analyses of the total lysates of U2OS cells silenced for control luciferase (siGL), Cep57 (siCep57), Cep63 (siCep63), or Cep152 (siCep152). The hit map on the right illustrates total protein levels of various centriolar components under the indicated conditions. Note that the depletion of Cep57, Cep63, or Cep152 greatly diminished the levels of inner PCM scaffolds and moderately those of procentriole and cartwheel proteins. The hit map (below) shows a pattern similar to that of (**a**). **(c, left)** Immunoblotting analyses of the total lysates of the indicated LCL cells bearing the indicated *CEP57* mutations. Arrowheads indicate the truncated Cep57 p.L309Pfs*9 mutant detected in the cells carrying the respective mutation. The p.E174Tfs*4 mutant was not clearly detectable. Numbers, relative signal intensities. The hit map (below) shows that, unlike those in (**a**) and (**b**), all the proteins analyzed are stable. **(c, right)** Confocal imaging and quantified data showing a substantial level of the p.L309Pfs*9 (and also likely p.E174Tfs*4) mutant localized to centrosomes, even though the antibody developed against the full-length Cep57 likely under-decorates these truncated mutants. Arrowheads, centrosome signals. Quantification was performed with images from three independent experiments [per experiment: $n \geq 54$ (total $n = 164$) for healthy control; $n \geq 51$ (total $n = 155$) for son; $n = 50$ (total $n = 150$) for daughter; $n \geq 56$ (total $n = 174$) for mother]. Bars, means of three experiments ± s.d.; *P* values, unpaired two-tailed *t*-tests. **(d, top)** Confocal imaging of the primary T cells purified from the indicated individuals (top). Quantified intensities for Cep57 and Cep152 are provided in Fig. 6a,c Arrowheads, centrosome signals. **(d, bottom)** The specificity of the Cep57 antibody used in this study is shown with quantified intensity data obtained from MEF $CEP57^{+/+}$ and $CEP57^{-/-}$ cells [3]. Arrowheads, centrosome sites. Quantification was performed with confocal images of immunostained cells from three independent experiments [per experiment: $n \geq 21$ (total $n = 64$) for $CEP57^{+/+}$; $n = 20$ (total $n = 60$) for $CEP57^{-/-}$]. Bars, means of three experiments ± s.d.; *P* values, unpaired two-tailed *t*-tests. Boxes, areas of enlargement. **(e)** Representative 3D-SIM images and quantification of immunostained primary T cells obtained from two independent experiments [per experiment: $n \geq 165$ (total $n = 338$) for healthy control; $n \geq 125$ (total $n = 290$) for son; $n \geq 216$ (total $n = 443$) for daughter; $n \geq 36$ (total $n = 97$) for father]. Bars, means of two experiments ± s.d.; *P* values, unpaired two-tailed *t*-tests. Boxes, areas of enlarge ment. **(f)** 3D-SIM imaging of the immunostained primary T cells obtained from the indicated individuals. Quantified data are provided in Fig. 6e,f. Boxes, areas of enlargement. **(g)** 3D-SIM imaging and quantification of acetylated tubulin and Cep192 signals showed that all the primary T cells examined exhibited no more than two acetylated tubulin signals, indicating that they did not duplicate centrioles. As expected from unstimulated lymphocytes in the blood, no mitotic cells were detected under our experimental conditions. Note that primary or naïve T cells in the bloodstream are in a quiescent state unless they are challenged by antigen stimulation. Boxes, areas of enlargement. **(h)** Representative 3D-SIM images showing PCNT signals decolocalized from the Cep192 location in the Δ*exon 3* mutant. Quantified PCNT intensities (right) and area (provided in Fig. 6g) were obtained from two independent experiments [per experiment: $n \geq 114$ (total $n = 251$) for healthy control; $n \geq 84$ (total $n = 202$) for son; $n \geq 86$ (total $n = 185$) for daughter; $n \geq 83$ (total $n = 173$) for father]. Bars, means of two experiments ± s.d.; *P* values, unpaired two-t ailed *t*-tests. **(i)** Quantification of centrosome-associated Cep192 intensities in T lymphocytes using images obtained as in (**f**). The data were obtained from two independent experiments [per experiment: $n = 100$ (total $n = 200$) for healthy control; $n = 100$ (total $n = 200$) for son; $n = 100$ (total $n = 200$) for daughter; $n \geq 70$ (total $n = 147$) for father]. Bars, means of two experiments ± s.d.; *P* values, unpaired two-tailed *t*-tests. **(j)** Immunoblotting and 3D-SIM imaging of U2OS cells expressing Cep57-Halo-SpoVM or its respective Cep57Δexon 3 mutant. Cep57 assemblies were decorated with a HaloTag JF646, while acetylated tubulin, Cep63, and Cep152 were detected by immunostaining. The presence or absence of acetylated tubulin signals indicates the centrosomal or ectopic (i.e., non-centrosomal) sites, respectively. The individual images are generated by enlarging the centrosome and the dotted square regions marked in the cells below. **(k)** Representative 3D-SIM images of U2OS cells showing that Cep57-Halo-SpoVM, but not the $Cep57^{\Delta exon\ 3}$-Halo-SpoVM, effectively recruits Cep63 but not PCNT to ectopic sites. Note that centrosomal localization of Cep63 and PCNT (indicated by the acetylated tubulin signals) is normal, as shown. Boxes, areas of enlargement. **(l)** A representative 3D-SIM image showing that Cep57-Halo-SpoVM-induced in vivo self-assemblies do not contain a detectable level of α-tubulin signals. Boxes, areas of enlargement. **(m)** Immunoprecipitation and immunoblotting analyses carried out with HEK293T cells co-transfected with the indicated constructs. Numbers, relative signal intensities. Asterisk, Flag-Cep63 degradation product. CBB, Coomassie Brilliant Blue.

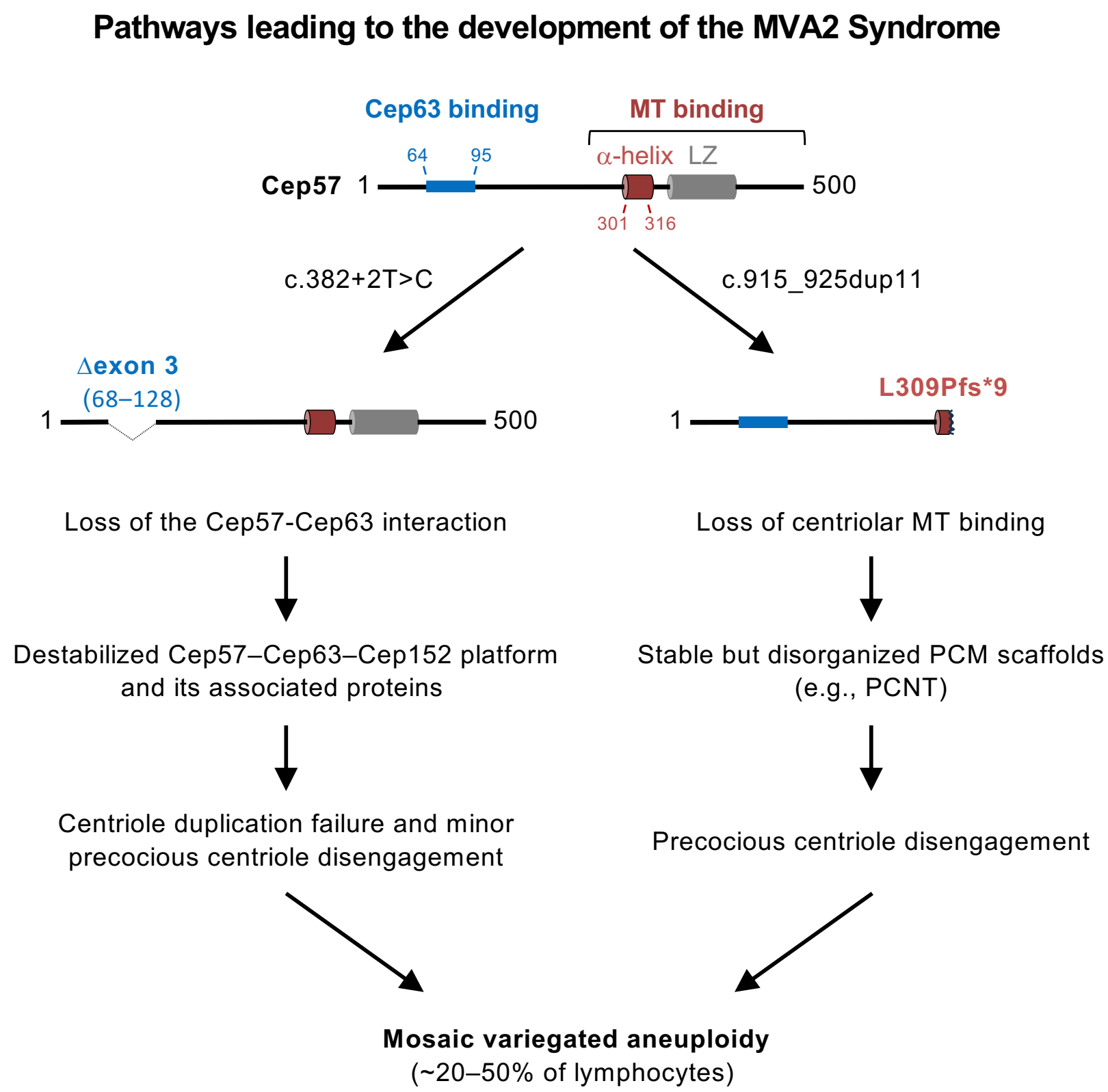

Supplementary Fig. 7. Schematic diagram illustrating pathways underlying the development of the MVA2 syndrome. The massive aneuploidies observed in the MVA2 syndrome can be caused by mutations depriving Cep57 of either Cep63 binding (Fig. 4) by the Δ*exon 3* mutation or centriolar MT-binding (Fig. 2) by the c.915_925dup11 mutation. This finding demonstrates the dual structural and functional properties of Cep57 required for anchoring Cep63–Cep152 assemblies to the centriolar MTs and properly organizing the scaffold proteins that associate with them, which are critical for Plk4-dependent centriole duplication.

Supplementary Table 1. Data collection and refinement statistics for Cep57(65–115) dimer (PDB: 21JQ)

	Cep57(65–115); Native	Cep57(65–115); Pt-derivative
Space group	$P6_122$	$P6_122$
Unit cell dimensions		
a, b, c (Å)	70.47, 70.47, 156.15	70.71, 70.71, 160.90
α, β, γ (°)	90, 90, 120	90, 90, 120
Wavelength (Å)	0.9795	1.0718
Resolution (Å)	50.0-2.1 (2.14-2.10)[b]	50.0-2.6 (2.64-2.60)[b]
R_{sym}[a]	8.2 (29.7)	11.9 (95.7)
$I/\sigma(I)$	29.7 (5.1)	57.9 (5.9)
Completeness (%)	92.8 (86.7)	98.6 (98.4)
Redundancy	5.0	29.4
**Refinement**		
Resolution (Å)	50.0-2.1	
Number of reflections	13,110	
R_{work}[c] / R_{free}	22.6 / 26.1	
Number of atoms		
Protein	897	
Water	99	
RMSD		
Bond lengths (Å)	0.008	
Bond angles (°)	0.963	
Ramachandran plot (%)		
Most favored region	100	
Average B-values (Å^2)		
Protein	35.2	
Water	41.1	

[a]$R_{sym} = \Sigma |I_{obs} - I_{avg}| / I_{obs}$, where I_{obs} is the observed intensity of individual reflection and I_{avg} is the average over symmetry equivalents.
[b]The numbers in parentheses are statistics from the shell with the highest resolution.
[c]$R_{work} = \Sigma ||F_o| - |F_c|| / \Sigma |F_o|$, where $|F_o|$ and $|F_c|$ are the observed and calculated structure factor amplitudes, respectively. R_{free} was calculated with 10% of the data.

Supplementary Table 2. The X-ray crystal structure of the Cep57(63–120)•Cep63(1–87) complex (PDB: 9I7Q)

	**Cep57(63–120)•Cep63(1–87)**
Space group	$P6_122$
Unit cell dimensions	
a, b, c (Å)	122.01, 122.01, 69.99
α, β, γ (°)	90, 90, 120
Wavelength (Å)	1.0000
Resolution (Å)	61.00-2.90 (3.08-2.90)[b]
R_{sym}[a]	8.2 (289.0)
$I/\sigma(I)$	24.2 (1.9)
Completeness (%)	100.0 (100.0)
Redundancy	37.6
**Refinement**	
Resolution (Å)	45.99-2.90
Number of reflections	7189
R_{work}[c] / R_{free}	24.6 / 26.5
Number of atoms	
Protein	1037
Ligand	5
RMSD	
Bond lengths (Å)	0.008
Bond angles (°)	1.310
Ramachandran plot (%)	
Most favored region	96
Average B-values (Å^2)	
Protein	85.6
Ligand	100.4

[a]$R_{sym} = \Sigma |I_{obs} - I_{avg}| / I_{obs}$, where I_{obs} is the observed intensity of individual reflection and I_{avg} is the average over symmetry equivalents.

[b]The numbers in parentheses are statistics from the shell with the highest resolution.

[c]$R_{work} = \Sigma ||F_o| - |F_c|| / \Sigma |F_o|$, where $|F_o|$ and $|F_c|$ are the observed and calculated structure factor amplitudes, respectively. R_{free} was calculated with 10% of the data.

Supplementary Table 3. Plasmid constructs used in this study

List	Protein	Expression vector	Restriction sites	Source
Mammalian expression constructs				
pKM8077	EGFP-Cep57	pEGFP-C1 vector	BglII, SalI	This study
pKM7399	EGFP-Cep57-N (1–269)	pHR′.J-CMV-SV-puro vector	BamHI, SalI	This study
pKM7400	EGFP-Cep57-C (265–500)	pHR′.J-CMV-SV-puro vector	BamHI, SalI	This study
pKM1234	HA-Cep57	pCI-neo-HA vector	SmaI (dead), NotI	[4]
pKM2797	Flag-Cep57	pCI-neo-Flag vector	PmeI, NotI	[4]
pKM7917	Flag-Cep57 4A (I69A, L73A, L76A, I80A)	pCI-neo-Flag vector	PmeI, NotI	This study
pKM7918	Flag-Cep57 2K (L73K, L76K)	pCI-neo-Flag vector	PmeI, NotI	This study
pKM7147	Flag-Cep57	pHR′.J-CMV-SV-puro vector	AscI, NotI (end-filled)	[4]
pKM7927	Flag-Cep57 4A (I69A, L73A, L76A, I80A)	pHR′.J-CMV-SV-puro vector	AscI, NotI (end-filled)	This study
pKM7928	Flag-Cep57 2K (L73K, L76K)	pHR′.J-CMV-SV-puro vector	AscI, NotI (end-filled)	This study
pKM8080	EGFP-Cep57 PP (V307P, M314P)	pEGFP-C1 vector	Synthetic (GenScript)	This study
pKM8049	Flag-Cep57 PP (V307P, M314P)	pHR′.J-CMV-SV-puro vector	Synthetic (GenScript)	This study
pKM8008	Flag-Cep57 ΔC1 (272–353)	pHR′.J-CMV-SV-puro vector	Synthetic (GenScript)	This study
pKM7581	EGFP-Cep57L1	pEGFP-C1 vector		[5]
pKM7948	Flag-Cep57L1	pCI-neo-Flag vector	PmeI, NotI	
pKM4858	GFP-Cep63	pCI-neo-GFP vector	PmeI, NotI	[6]
pKM2798	Flag-Cep63	pCI-neo-Flag vector	PmeI, NotI	[6]
pKM1235	HA-Cep63	pCI-neo-HA vector	SmaI (dead), NotI	[6]
pKM7919	Flag-Cep63-*sil* 4A (L25A, L28A, M29A, I32A)	pCI-neo-Flag vector	PmeI, NotI	This study
pKM7920	Flag-Cep63-*sil* 2K (L28K, I32K)	pCI-neo-Flag vector	PmeI, NotI	This study
pKM7921	Flag-Cep63-*sil* 2D (M1D, L5D)	pCI-neo-Flag vector	PmeI, NotI	This study
pKM7922	Flag-Cep63-*sil* Δ11 (1–11 deletion)	pCI-neo-Flag vector	PmeI, NotI	This study
pKM5659	Flag-Cep63-*sil*	pHR′.J-CMV-SV-puro vector	AscI, SalI	[6]
pKM7923	Flag-Cep63-*sil* 4A	pHR′.J-CMV-SV-puro vector	AscI, SpeI	This study
pKM7924	Flag-Cep63-*sil* 2K	pHR′.J-CMV-SV-puro vector	AscI, SpeI	This study
pKM7925	Flag-Cep63-*sil* 2D	pHR′.J-CMV-SV-puro vector	AscI, SpeI	This study
pKM7926	Flag-Cep63-*sil* Δ11 (1–11 residue deletion)	pHR′.J-CMV-SV-puro vector	AscI, SpeI	This study
pKM8289	Cep57-HaloTag-SpoVM	pHR′.J-CMV-SV-puro	AscI, NotI	This study

		vector	(end-filled)	
pKM8292	Cep57Δexon 3-HaloTag-SpoVM	pHR′.J-CMV-SV-puro vector	AscI, XhoI	This study
pKM8312	Flag-NuSAP1	pHR′.J-CMV-SV-puro vector	AscI, BamHI	This study
E. coli expression constructs				
pKM7953	Cep57-C (272–500)	His$_6$-MBP-TEV vector	AscI, XhoI	This study
pKM7963	Cep57-C1 (272–353)	His$_6$-MBP-TEV vector	AscI, XhoI	This study
pKM7965	Cep57-C2 (353–450)	His$_6$-MBP-TEV vector	AscI, XhoI	This study
pKM7967	Cep57-C3 (442–500)	His$_6$-MBP-TEV vector	AscI, XhoI	This study
pKM8021	Cep57-C (272–500) PP	His$_6$-MBP-TEV vector	Genscript	This study
pKM7954	Cep57L1-C (253–460)	His$_6$-MBP-TEV vector	AscI, XhoI	This study
pKM8072	Cep57 (277–334)	His$_6$-MBP-TEV vector	NdeI, NotI	This study
pKM8333	Cep57 (277–334) PP	His$_6$-MBP-TEV vector	NdeI, NotI	This study
pKM7332	Cep57 (65–115)	His$_6$-MBP-TEV vector	NdeI, XhoI	This study
pKM7287	Cep57 (63–120)	pETDuet-1 vector	BamHI, NotI	[4]
	Cep63 (1–87)		NdeI, XhoI	
pKM7916	Cep57 (63–120)	pETDuet-1 vector	BamHI, NotI	This study
	Cep63 (12–87)		NdeI, XhoI	
pKM7224	Cep57 (63–120)	His$_6$-MBP-TEV vector	NdeI, XhoI	This study
pKM8208	His-Cep57 (1–67)-(GGGGS)$_3$-HaloTag-GGGGS-mSA	pETDuet-1 vector	BamHI, SacI	This study
pKM8209	His-Cep57 (1–120)-(GGGGS)$_3$-HaloTag-GGGGS-mSA	pETDuet-1 vector	BamHI, SacI	This study
pKM7545	mCherry-Cep63	pETDuet-1 vector	BamHI, NotI	This study
	mGFP-Cep152 (924–1295)		NdeI, XhoI	

Supplementary Table 4. siRNA sequences used in this study

Target Gene	Sequence (nt positions from the start codon)	Source	Type
Luciferase (GL)	CGTACGCGGAATACTTCGA	[7]	Synthetic
Cep57	CTCAATGTTAAAGCATTTAAATG (3′-UTR)	[4]	Synthetic
	GTGTGATTTTTTAAAATAGTTCT (3′-UTR)	[4]	Synthetic
Cep57L1	GAGAAGCAACTAGAATATA (nt 388–406) (Life Technologies, s226224)	[8]	Synthetic
	GCTTCAAACTGGACTTGAA (nt 657–675)	[5]	Synthetic
Cep63	GGAGCTCATGAAACAGATT (nt 78–96)	[9]	Synthetic
Cep152	GCGGATCCAACTGGAAATCTA (nt 3099–4019)	[10]	Synthetic

Supplementary Table 5. Antibodies used in this study

Antibodies	Species	Cat #	Source	Exp. dilution
Primary antibodies				
Anti-Flag (Clone M2) Affinity Gel	Mouse	A2220	Sigma	2 µg (IP)
Anti-Flag M2-Peroxidase (HRP)	Mouse	A8592	Sigma	1:10000 (IB)
Alexa Fluor 488–conjugated anti-Flag	Rabbit	5407S	Cell signaling Technology	1:100 (IF)
Anti-HA (Clone 3F10)	Rat	11867423001	Roche	1:1000 (IB)
Anti-GFP	Rabbit	Sc-8334	Santa Cruz	1:1000 (IB)
Anti-GFP	Rat	D153-8	MBL International	2 µg (IP)
Anti-MBP (8G1)	Mouse	2396	Cell signaling Technology	1:200 (IF)
Anti-Halo	Mouse	G921A	Promega	1:1000 (IB)
Anti-Cep57 (N1C1; Epitope: center region)	Rabbit	GTX115931	GeneTex	1:200 (IF)
Anti-Cep57 (Full-length)	Rat	Gift	[5]	1:1000 (IB), 1:200 (IF)
Anti-Cep57 (1-240)	Rabbit	PA5-98561	Invitrogen	1:1000 (IB)
Anti-Cep57L1 (1–64)	Rabbit	24957–1-AP, Gift	Proteintech, [8]	1:1000 (IB), 1:200 (IF)
Anti-Cep63 (Full-length)	Rabbit	06–1292	Millipore	1:1000 (IB), 1:100 (IF)
Anti-Cep152 (491–810)	Rabbit	Lab supply	[11]	1:100 (IF)
Anti-Cep152 (P7; 1350–1654)	Rabbit	Lab supply	This study	1:1000 (IB)
Alexa Fluor 647–conjugated anti-Cep152 (491-810)	Rabbit	Lab supply	This study	1:100 (IF)
Anti-Cep152 (N-terminal; GT1285)	Mouse	GTX631486	GeneTex	1:200 (IF)
Anti-Cep192 (1–647)	Rabbit	Lab supply	[11]	1:100 (IF)
Anti-Cep192 (1800-1850)	Rabbit	NBP1-28718	Novus	1:1000 (IB)
Alexa Fluor 594–conjugated anti-Cep192 (1-647)	Rabbit	Lab supply	This study	1:100 (IF)
Anti-Plk4 (6H5)	Mouse	MABC544	Millipore	1:100 (IB)
Anti-STIL (1237–1287)	Rabbit	Ab89314	Abcam	1:1000 (IB)
Anti-SAS6 (404–657)	Mouse	Sc-81431	Santa Cruz	1:200 (IB), 1:100 (IF)
Anti-CP110	Rabbit	12780-1-AP	Proteintech	1:2000 (IF)
Anti-CP110 (600-650)	Rabbit	A301-343	Bethyl	1:1000 (IB)
Anti-Centrin (Clone 20H5)	Mouse	04-1624	Millipore	1:200 (IF)
Anti-PCNT	Rabbit	Gift	[12]	1:100 (IB); 1:200 (IF)
Anti-Polyglutamylation modification (GT335)	Mouse	AG-20B-0020-C100	AdipoGen Life Science	1:300 (IF)
Anti-Acetylated tubulin (6-11B-1	Mouse	T7451	Sigma	1:300 (IF)
Anti-CPAP	Rabbit	11517-1-AP	Proteintech	1:1000 (IB)
Anti-Cep78	Rabbit	A301-800A	Bethyl	1:1000 (IB)
Anti-γ-tubulin	Mouse	T5326	Sigma	1:1000 (IB)
Anti-α-tubulin (B-5-1-2)	Mouse	T6074	Sigma	1:1000 (IB), 1:200 (IF)
Secondary antibodies (Immunofluorescence)				

Anti-rabbit IgG Alexa Fluor 647-conjugated	Donkey	A-31573	Invitrogen	1:300 (IF)
Anti-rabbit IgG Alexa Fluor 594-conjugated	Donkey	A-21207	Invitrogen	1:300 (IF)
Anti-rabbit IgG Alexa Fluor 488-conjugated	Donkey	A-21206	Invitrogen	1:300 (IF)
Anti-mouse IgG Alexa Fluor 647-conjugated	Donkey	A-31571	Invitrogen	1:300 (IF)
Anti-mouse IgG Alexa Fluor 594-conjugated	Donkey	A-21203	Invitrogen	1:300 (IF)
Anti-mouse IgG Alexa Fluor 488-conjugated	Donkey	A-21202	Invitrogen	1:300 (IF)
Anti-mouse IgG Alexa Fluor 405-conjugated	Goat	A-31553	Invitrogen	1:300 (IF)
Anti-rat IgG Alexa Fluor 647-conjugated	Donkey	A78947	Invitrogen	1:300 (IF)
Anti-rat IgG Alexa Fluor 594-conjugated	Donkey	A-21209	Invitrogen	1:300 (IF)
Anti-rat IgG Alexa Fluor 488-conjugated	Donkey	A-21208	Invitrogen	1:300 (IF)
F(ab')2 Anti-mouse IgG Alexa Fluor 488-conjugated	Goat	A-11017	Invitrogen	1:400 (IF)
F(ab')2 Anti-mouse IgG Alexa Fluor 594-conjugated	Goat	A-11020	Invitrogen	1:400 (IF)
F(ab')2 Anti-rabbit IgG Alexa Fluor 488-conjugated	Goat	A-11070	Invitrogen	1:400 (IF)
F(ab')2 Anti-rabbit IgG Alexa Fluor 594-conjugated	Goat	A-11072	Invitrogen	1:400 (IF)
Secondary antibodies (Immunoblot)				
Anti-rabbit IgG HRP-conjugated	Donkey	NA9340	GE Healthcare	1:3000 (IB)
Anti-mouse IgG HRP-conjugated	Donkey	NA9310	GE Healthcare	1:3000 (IB)
Anti-rat IgG HRP-conjugated	Goat	SA00001-15	Proteintech	1:3000 (IB)

Supplementary References:

1 Sukla, S. *et al.* Crystal structure of human Cep57 C-terminal domain reveals the presence of leucine zipper and the potential microtubule binding region. *Proteins* **92**, 1137-1143 (2024). https://doi.org/10.1002/prot.26698
2 Yeh, H. W. *et al.* Cep57 regulates human centrosomes through multivalent interactions. *Proc Natl Acad Sci U S A* **121**, e2305260121 (2024). https://doi.org/10.1073/pnas.2305260121
3 Aziz, K. *et al.* Mosaic-variegated aneuploidy syndrome mutation or haploinsufficiency in Cep57 impairs tumor suppression. *J Clin Invest* **128**, 3517-3534 (2018). https://doi.org/10.1172/JCI120316
4 Wei, Z. *et al.* Requirement of the Cep57-Cep63 interaction for proper Cep152 recruitment and centriole duplication. *Mol. Cell. Biol.* **40**, e00535-00519 (2020). https://doi.org/10.1128/MCB.00535-19
5 Zhao, H. *et al.* Cep57 and Cep57l1 function redundantly to recruit the Cep63-Cep152 complex for centriole biogenesis. *J. Cell Sci.* **133**, jcs241836 (2020). https://doi.org/10.1242/jcs.241836
6 Kim, T. S. *et al.* Molecular architecture of a cylindrical self-assembly at human centrosomes. *Nat. Commun.* **10**, 1151 (2019). https://doi.org/10.1038/s41467-019-08838-2
7 Elbashir, S. M. *et al.* Duplexes of 21-nucleotide RNAs mediate RNA interference in cultured mammalian cells. *Nature* **411**, 494-498 (2001).
8 Ito, K. K. *et al.* Cep57 and Cep57L1 maintain centriole engagement in interphase to ensure centriole duplication cycle. *J. Cell Biol.* **220**, e202005153 (2021). https://doi.org/10.1083/jcb.202005153
9 Zhao, H. *et al.* The Cep63 paralogue Deup1 enables massive de novo centriole biogenesis for vertebrate multiciliogenesis. *Nat. Cell Biol.* **15**, 1434-1444 (2013).
10 Cizmecioglu, O. *et al.* Cep152 acts as a scaffold for recruitment of Plk4 and CPAP to the centrosome. *J. Cell Biol.* **191**, 731-739 (2010).
11 Kim, T.-S. *et al.* Hierarchical recruitment of Plk4 and regulation of centriole biogenesis by two centrosomal scaffolds, Cep192 and Cep152. *Proc. Natl. Acad. Sci. USA* **110**, E4849-4857 (2013).
12 Kim, K. & Rhee, K. The pericentriolar satellite protein CEP90 is crucial for integrity of the mitotic spindle pole. *J Cell Sci* **124**, 338-347 (2011). https://doi.org/10.1242/jcs.078329

Source files for immunoblotting analyses

Fig. 2b

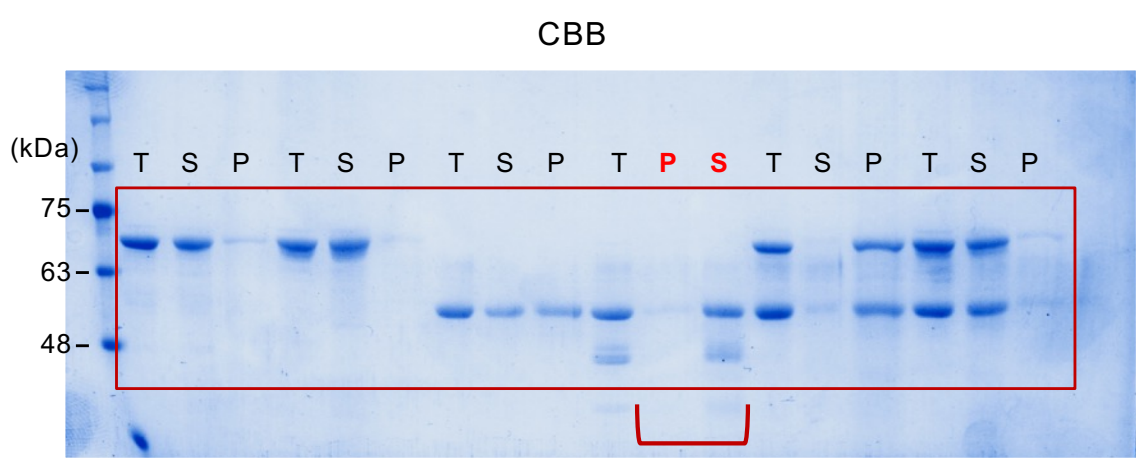

For the Fig. 2b in the text, the "S" & "P" lanes were cut out and flipped to present them in the correct order.

Fig. 2g

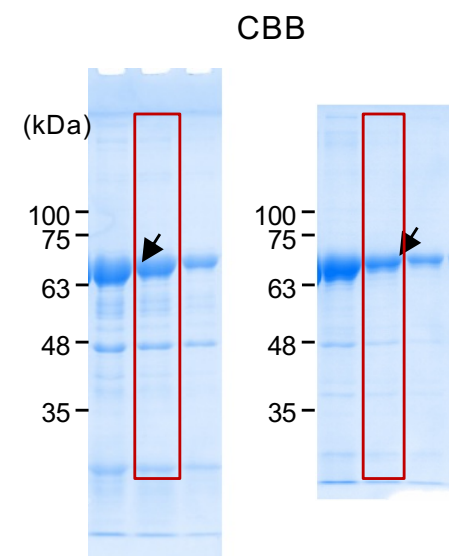

Fig. 2h

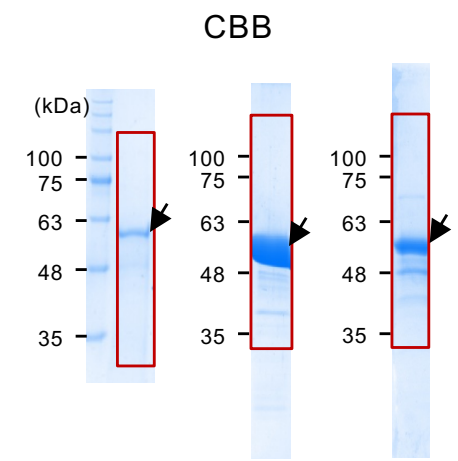

Fig. 3b

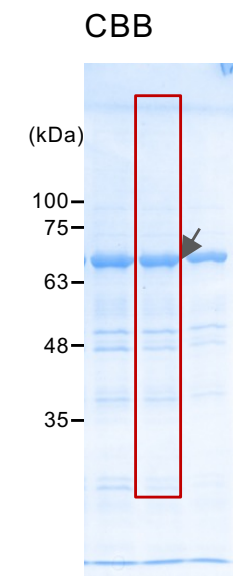

Fig. 3c

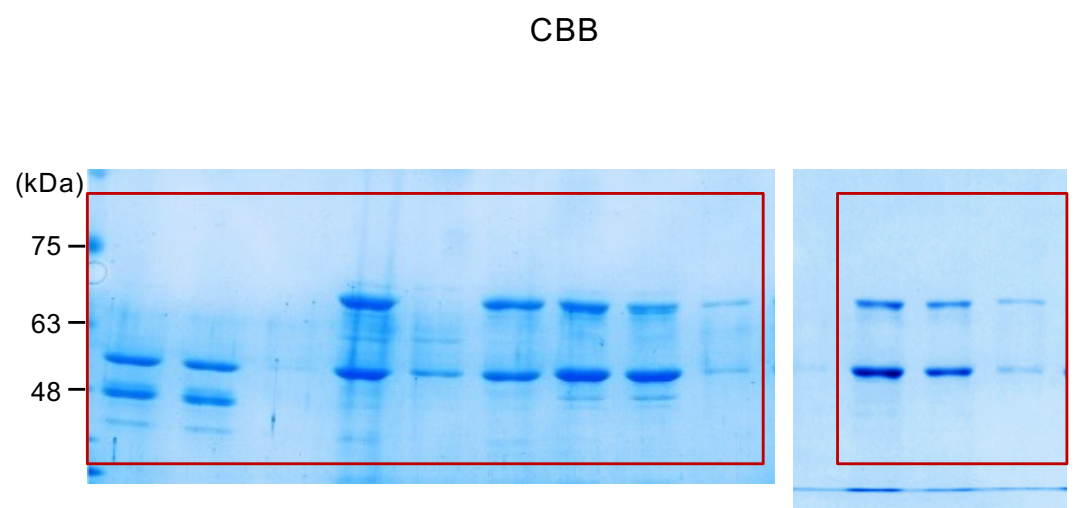

Fig. 3e

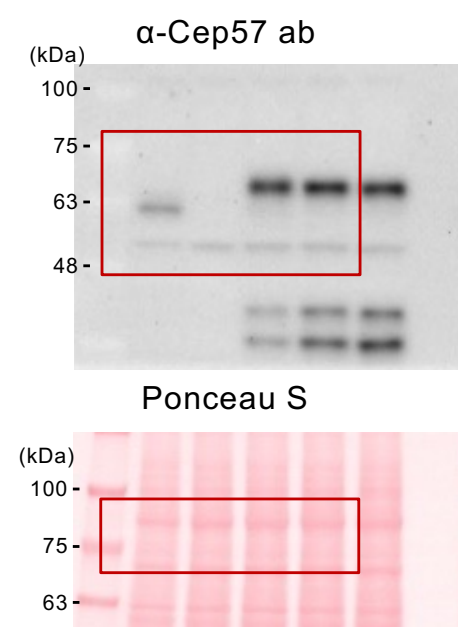

Fig. 4g

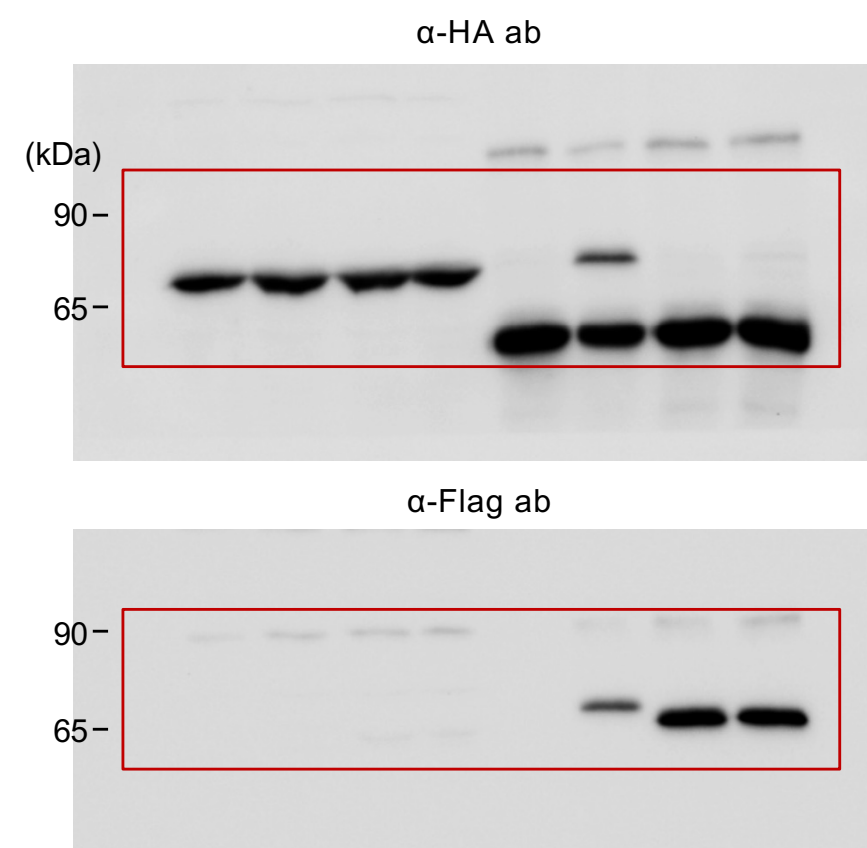

Fig. 4h

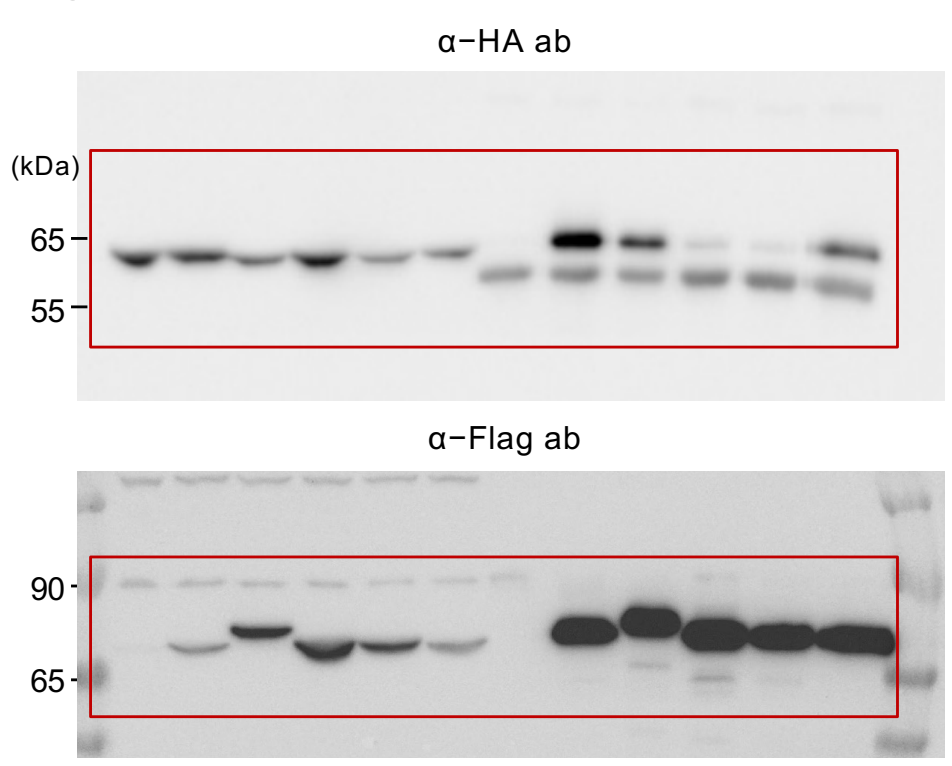

Fig. 5a

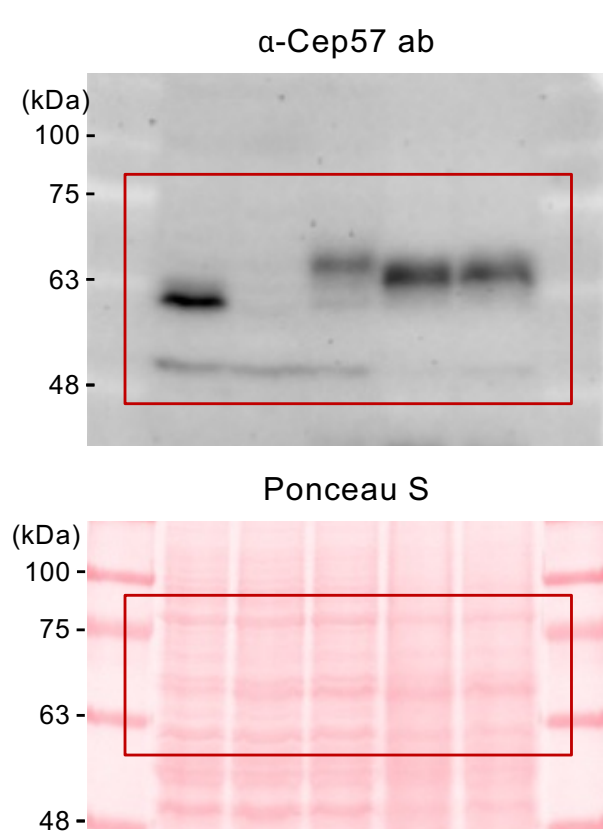

Fig. 5d

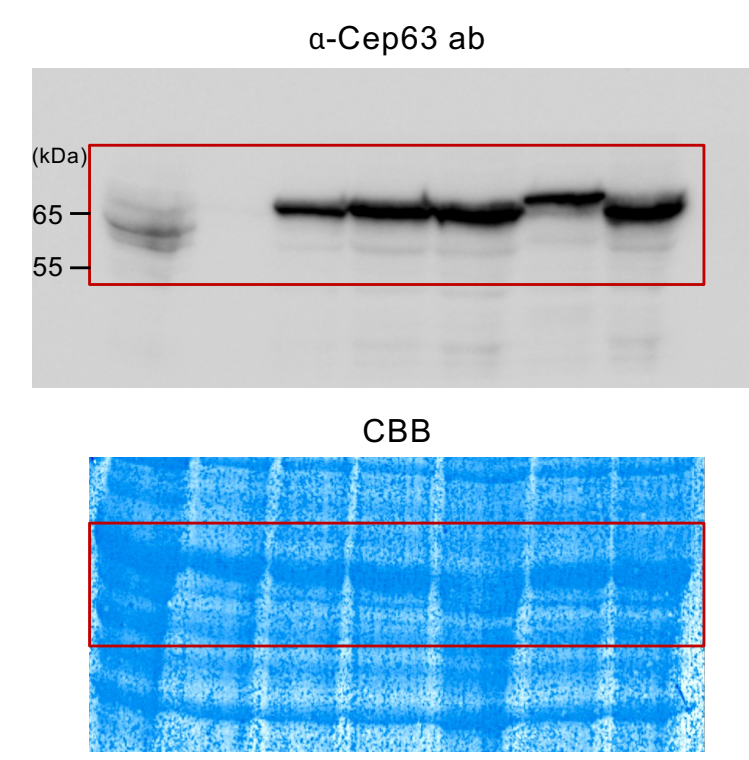

Source files for immunoblotting analyses

Supplementary Fig. 2f

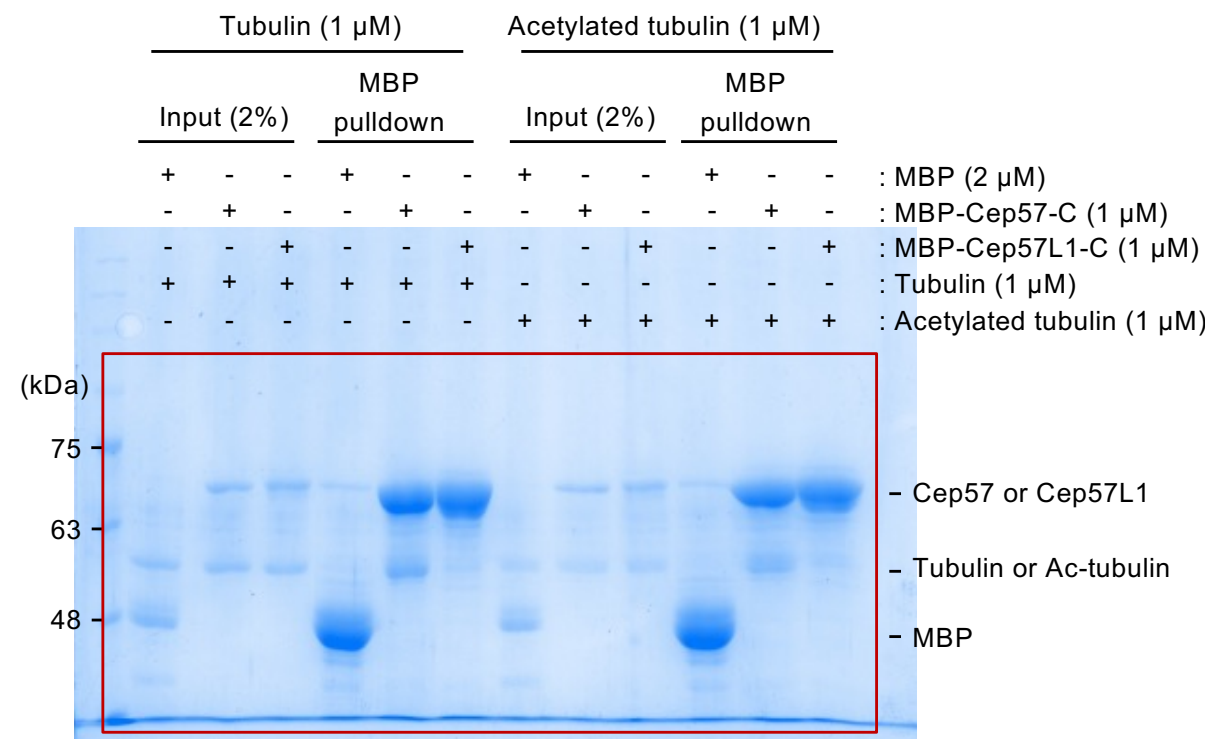

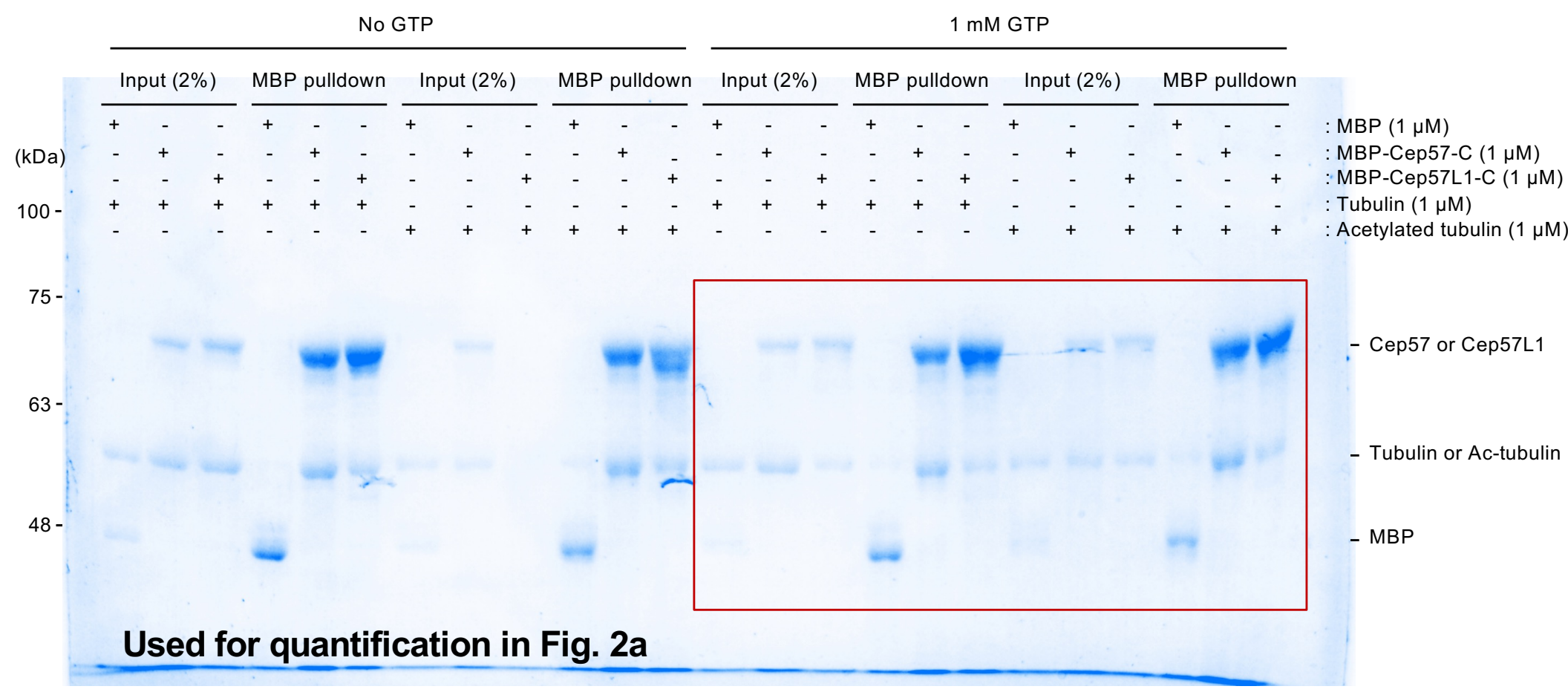

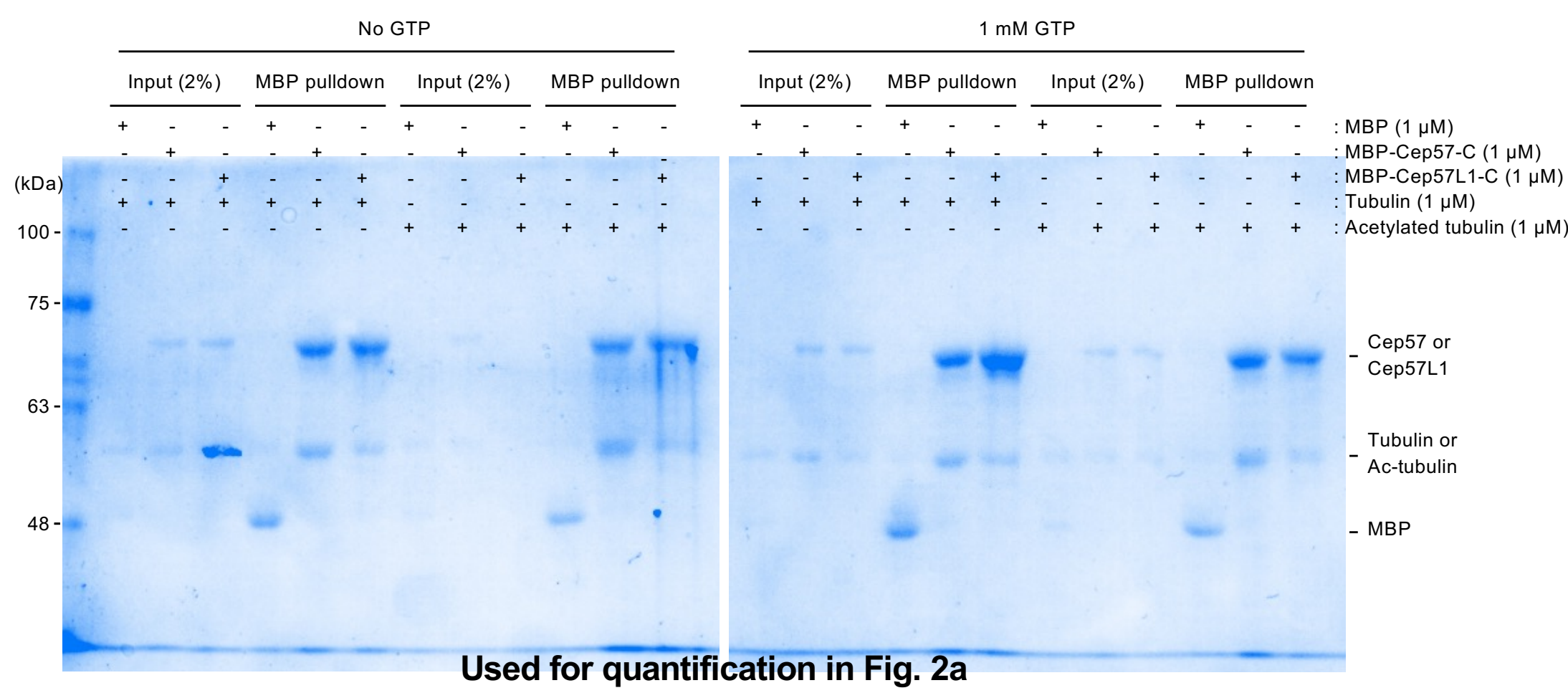

Supplementary Fig. 2g

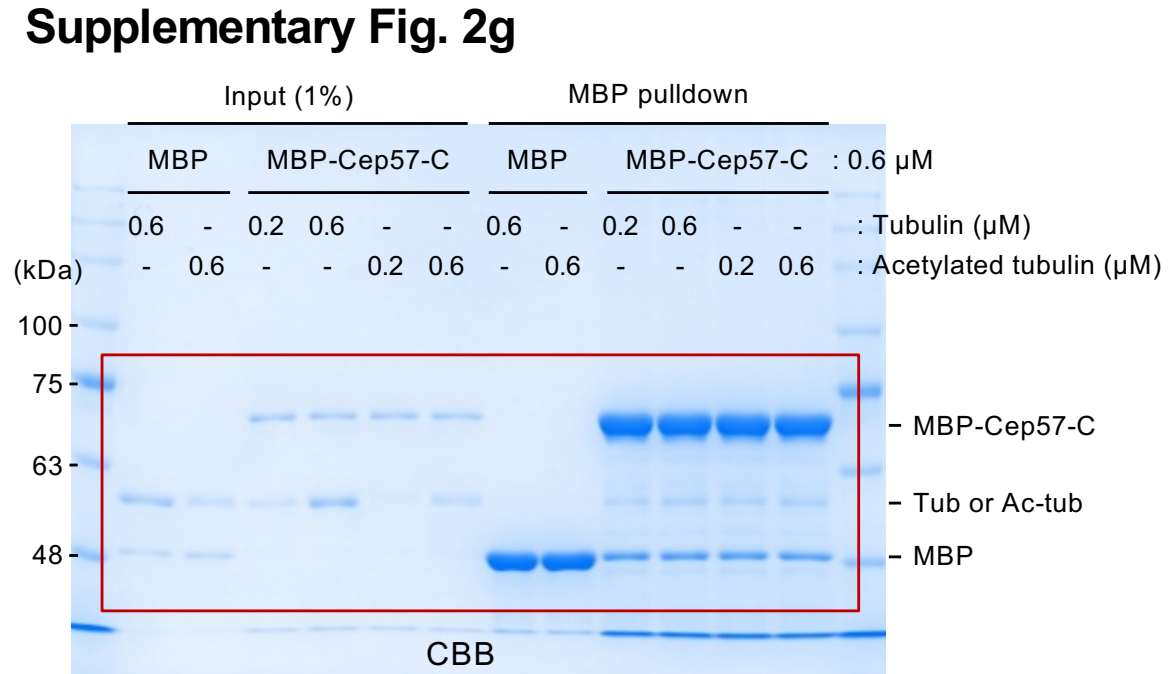

Supplementary Fig. 2h

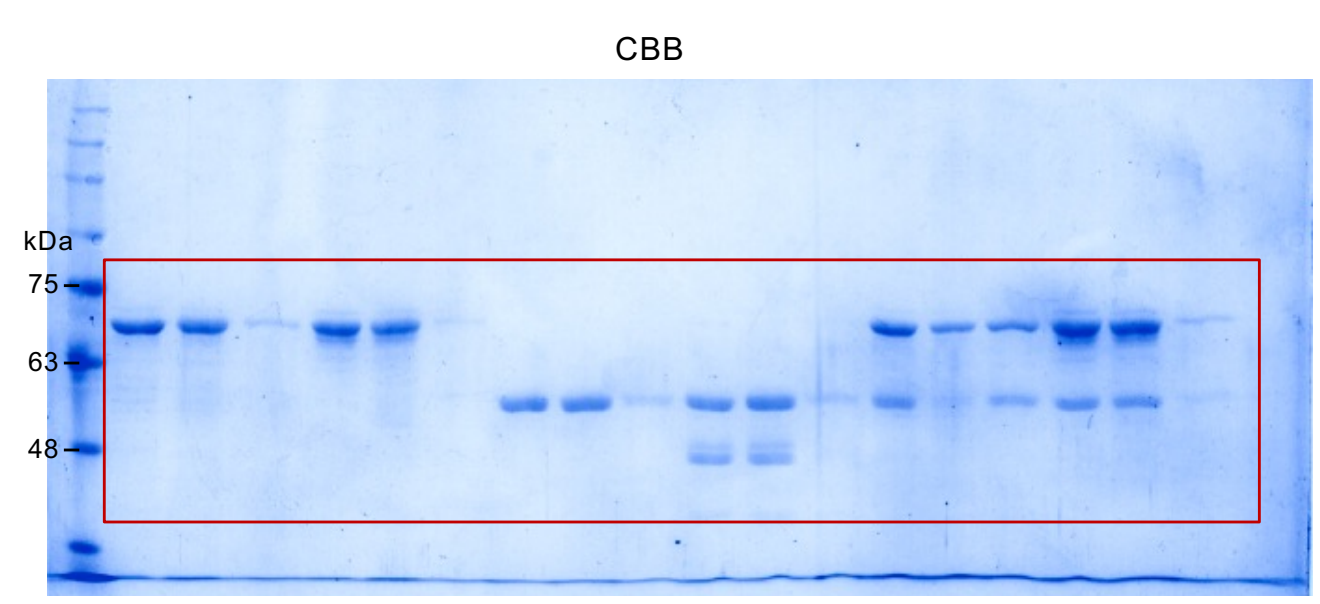

Source files for immunoblotting analyses

Supplementary Fig. 2p

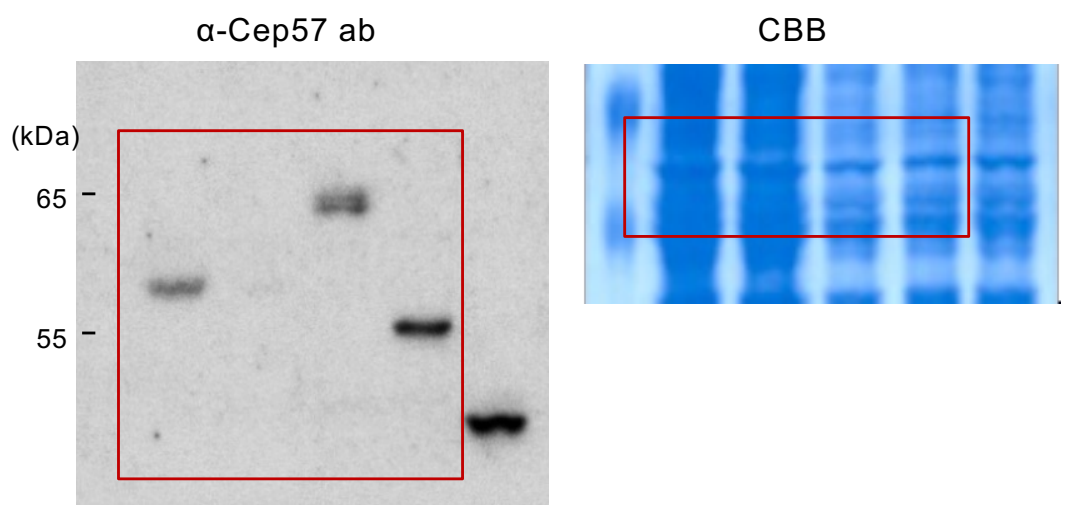

Supplementary Fig. 3a

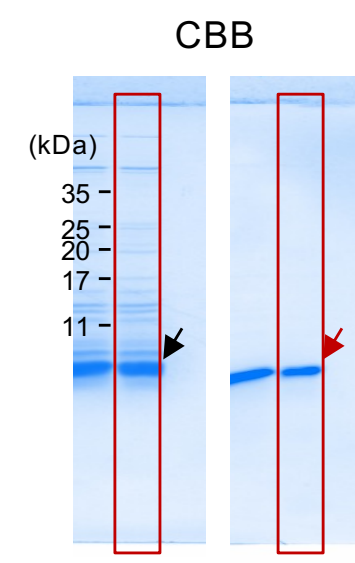

Supplementary Fig. 3b

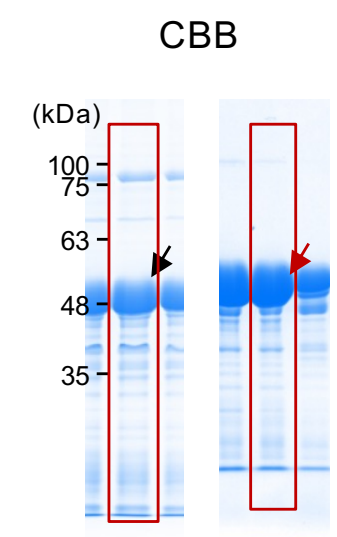

Supplementary Fig. 4a

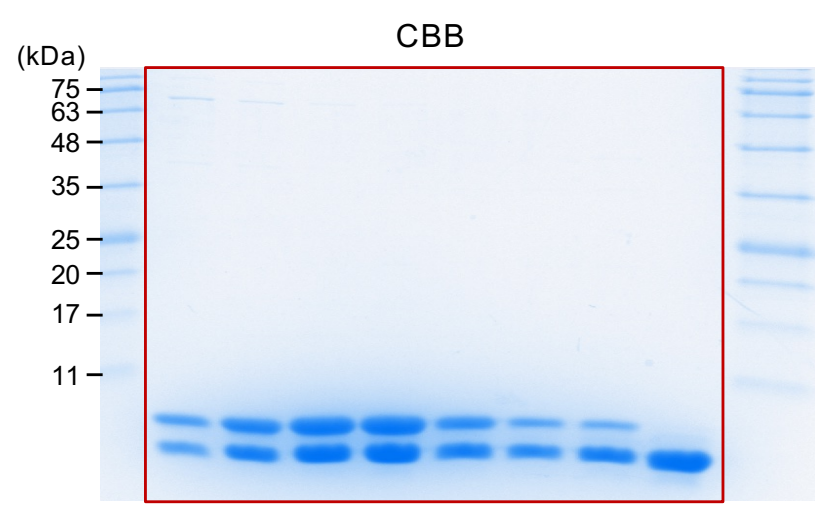

Supplementary Fig. 6b

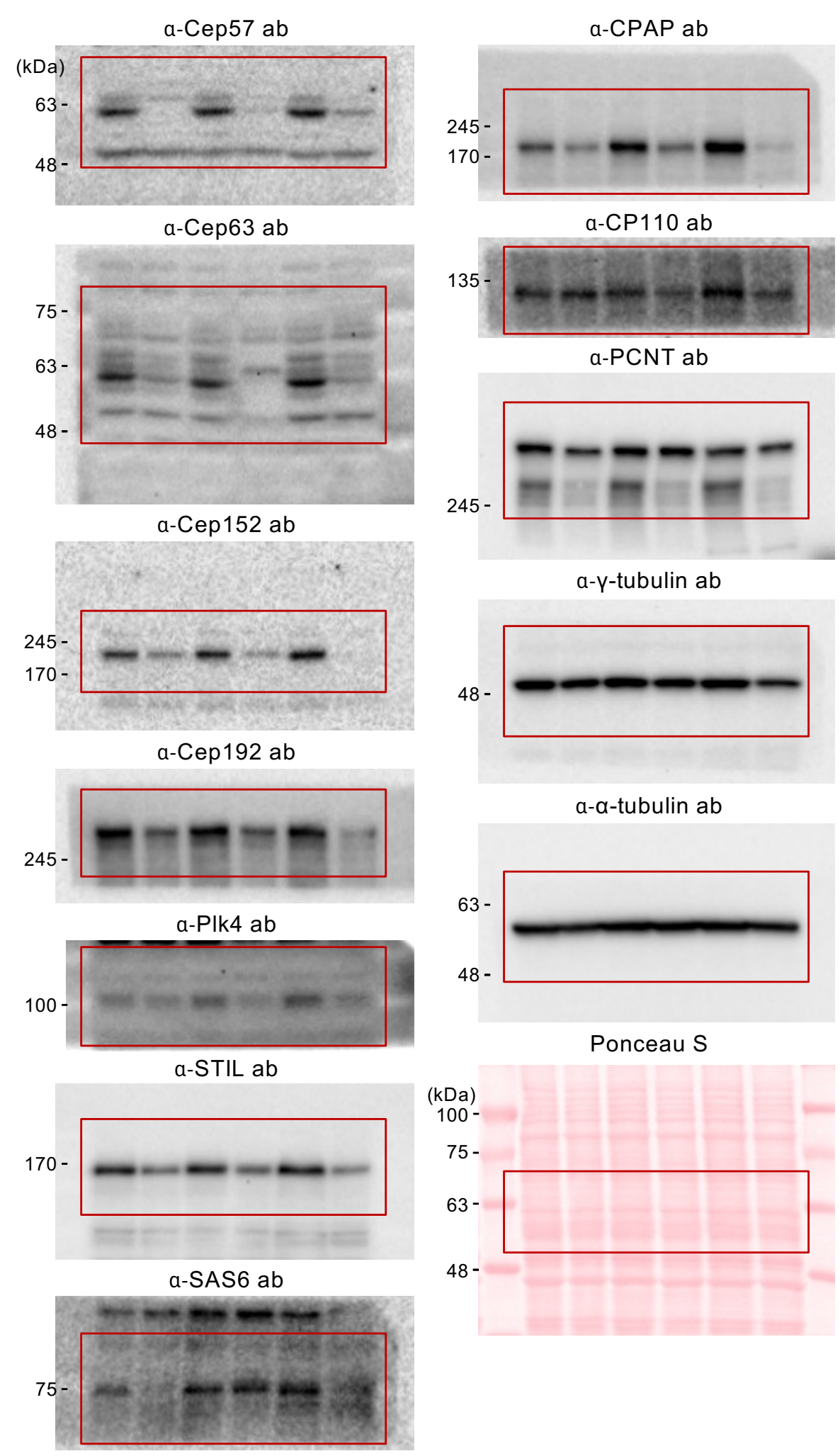

Supplementary Fig. 6a

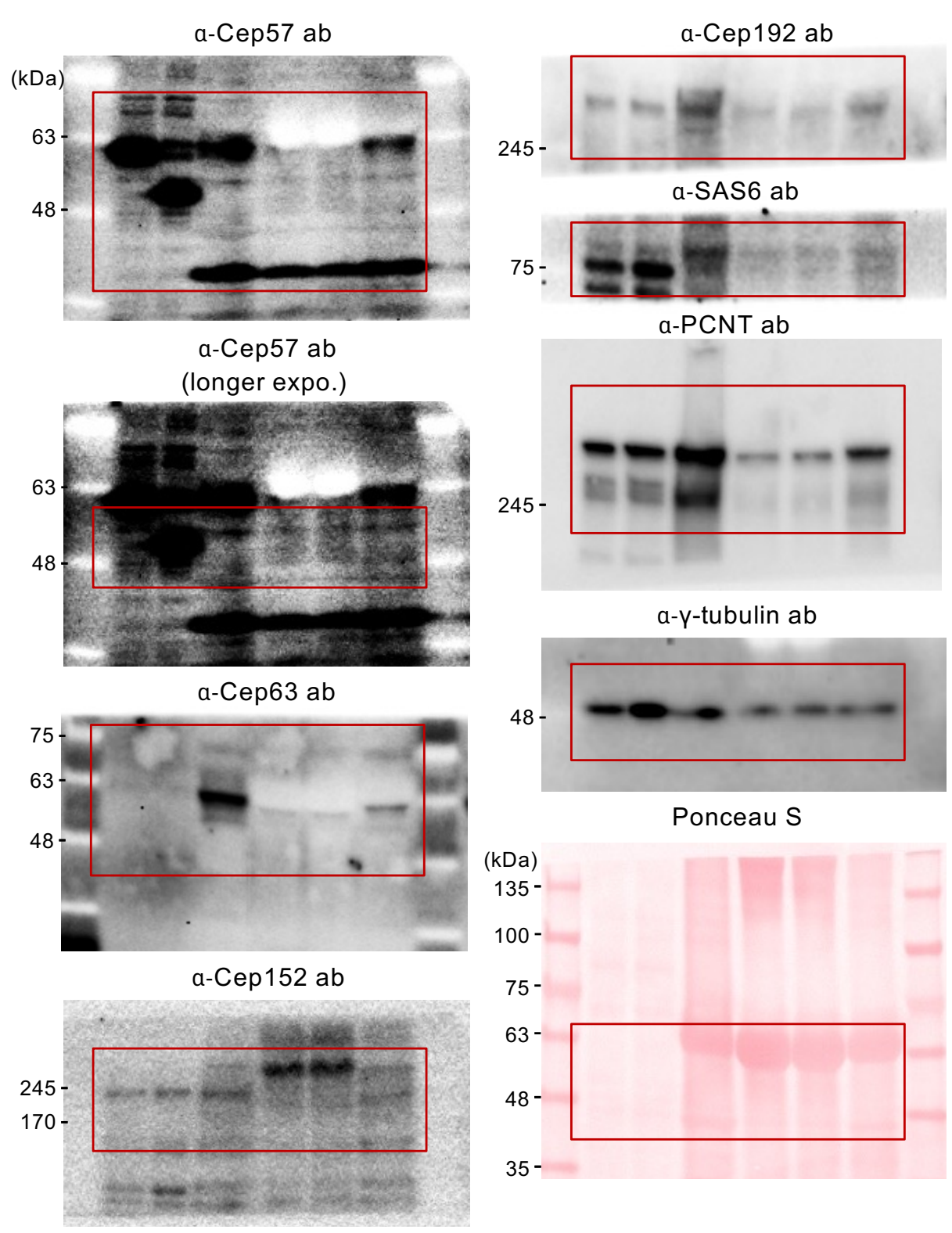

Source files for immunoblotting analyses

Supplementary Fig. 6c

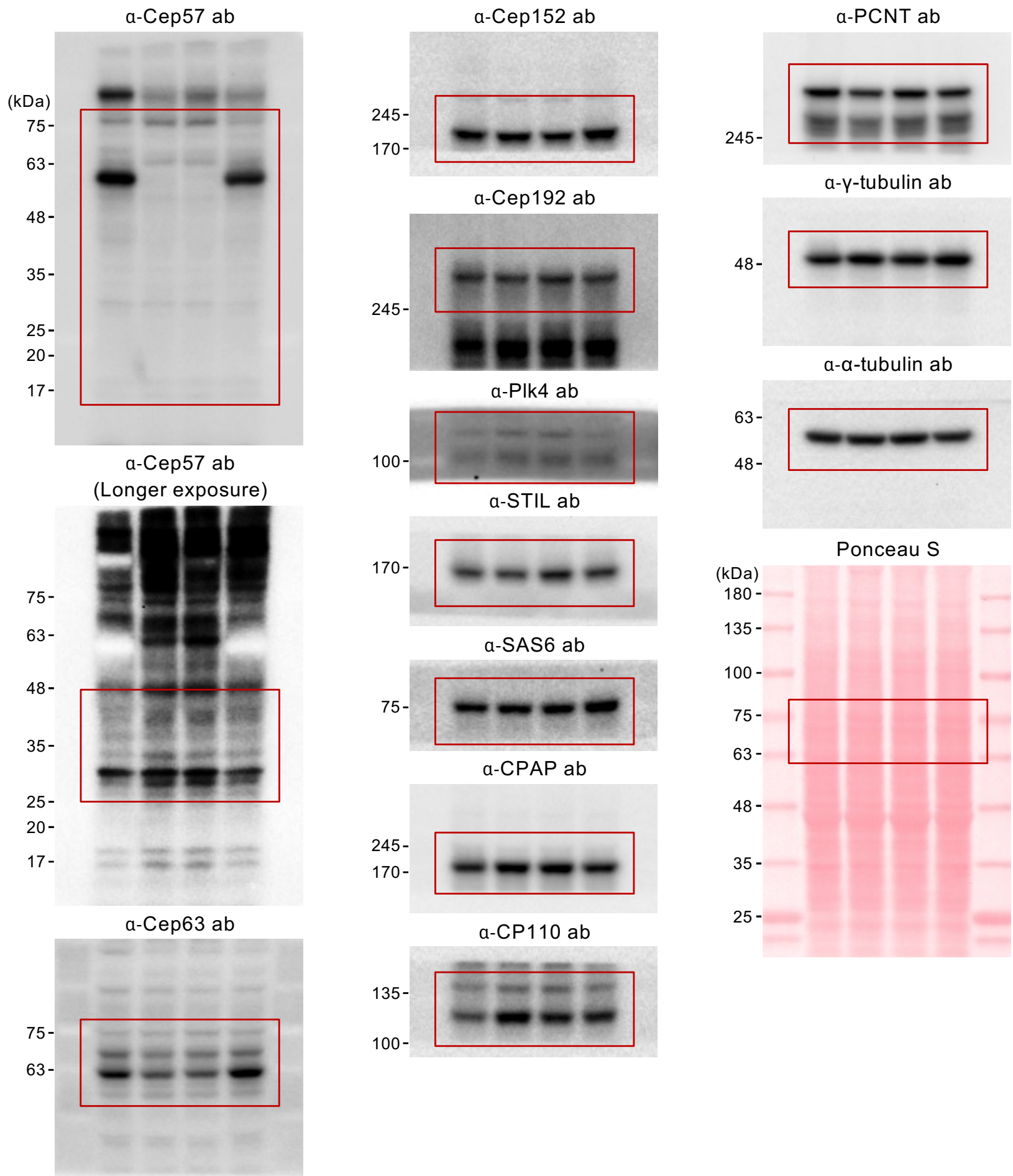

Supplementary Fig. 6j

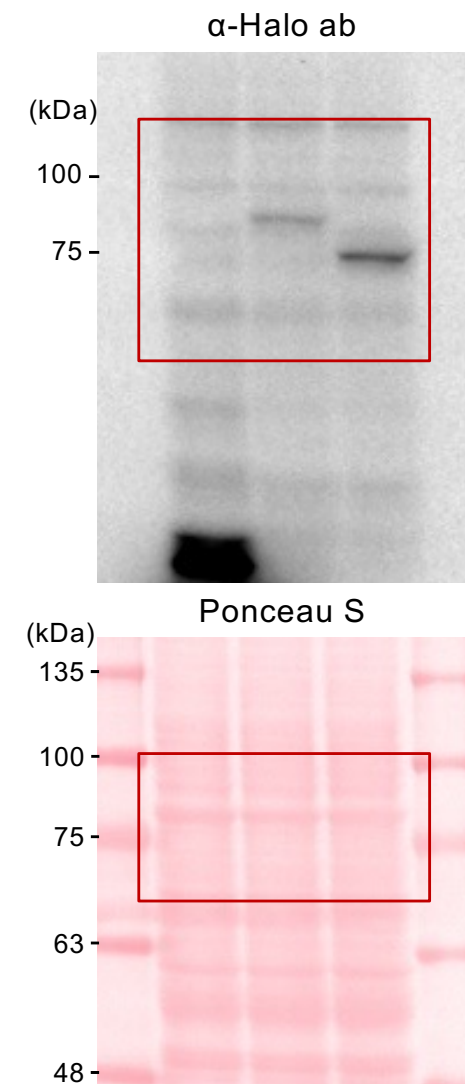

Supplementary Fig. 6m

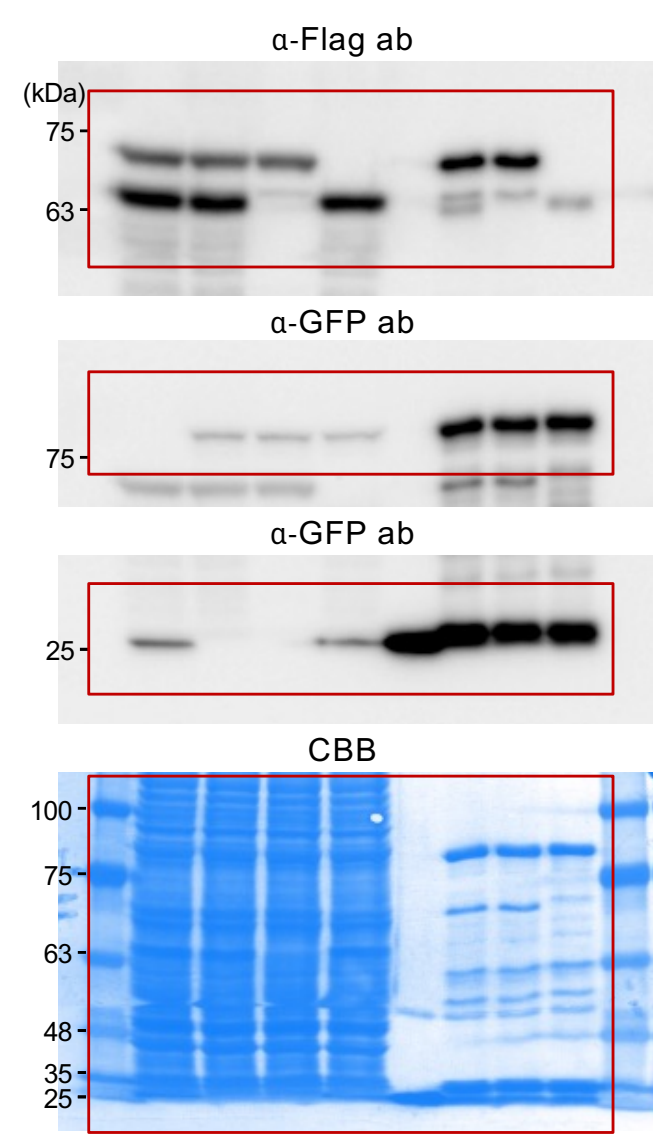

Supplement: Supplementary file 1 [file 41467_2026_77795_MOESM1_ESM.pdf]
