# Supplementary material for: Cep57 organizes pericentriolar material through dual architectural modules and prevents human mosaic variegated aneuploidy
Source: Nat Commun. 2026 Sep 30;17:11262. doi: 10.1038/s41467-026-77795-4 (PMC13720213; doi:10.1038/s41467-026-77795-4)
Supplement: Supplementary file 2 [file 41467_2026_77795_MOESM2_ESM.pdf]

nature portfolio

Peer Review File

Cep57 Organizes Pericentriolar Material Through Dual Architectural Modules and Prevents Human Mosaic Variegated Aneuploidy

Corresponding Author: Dr Kyung Lee

This file contains all reviewer reports in order by version, followed by all author rebuttals in order by version.

Version 0:

Reviewer comments:

Reviewer #1

(Remarks to the Author)
This is an excellent paper detailing interactions between Cep63 and Cep57(Cep192) and Cep152 that brings new insight into the control of centriole duplication. The study maps the interaction between Cep57 and microtubules and also with Cep63. The region interacting with Cep63 is deleted in the mosaic variegated aneuploidy (MVA) genetic disorder characterized by chromosome missegregation. Cep63 also binds Cep192, the partner of Plk4 that drives centriole duplication. I have no concerns about the data and I believe that the body of the paper is acceptable for publication without needing any modification. The only change I would suggest to the authors is to re-write their abstract to make it more widely accessible. This would mean removing the detailed description of precise defects and giving a description of the molecular interactions they have uncovered and ther importance for recruiting Plk4 and peri-centriolar material. In other words those features that lead to the model presented in Fig. 7.

(Remarks on code availability)

Reviewer #2

(Remarks to the Author)
This work consolidates and extends current understanding of PCM organization by providing structural insight into how Cep57 integrates into the Cep63–Cep152 scaffold through a combination of super-resolution imaging, structural biology, and biochemical reconstitution. The study identifies a helical region (residues 301–316) of Cep57 that is critical for Cep57-C oligomerization, microtubule binding, and centrosomal localization. In addition, it defines the binding interface of the Cep57–Cep63 complex. Functional mutagenesis further demonstrates that this interaction is essential for the stability of the Cep57–Cep63–Cep152 structural platform and for Plk4-dependent centriole duplication. These findings provide a mechanistic explanation for how exon 3 deletion in Cep57 disrupts this structural axis, thereby linking genetic observations in MVA2 syndrome to defects in PCM organization. However, rather than introducing a fundamentally new conceptual framework, this study builds upon established models of centrosomal architecture and centriole duplication by defining molecular interfaces and clarifying the architectural hierarchy that stabilizes PCM attachment to centriolar microtubules.

Overall, the work represents a careful and meaningful refinement of the current centrosome assembly model (PMID: 32152252, PMID: 32503940, PMID: 30858376; PMID: 37707473), adding structural resolution and disease relevance. While it provides valuable mechanistic detail, its advances are incremental in nature and may be better suited for a specialized journal with a focused readership in centrosome and cell division biology.

COMMENTS

Figure 2a. Cep57 appears to bind more strongly to MTs than to acetylated MTs. Could the authors comment on the possible basis for this difference? If this effect is related to MT dynamics or stability, it would be helpful to clarify whether Taxol treatment alters this binding behavior, for example by performing MT pelleting experiments.

Figure 2c. It would be helpful to include control experiments using MBP–Cep57-C alone to clarify that the observed absorbance signal reflects MT polymerization rather than potential aggregation of Cep57 (Fig. 2f). This would strengthen confidence in the interpretation of the assay.

Figure 2e. MTs appear more curved in the presence of CEP57-C. Could the authors clarify what might account for this increased curvature?

The authors state that Cep57 (301–316) adopts a helical conformation. It would be helpful to provide direct structural evidence supporting this claim. For example, circular dichroism analysis of this fragment without the MBP tag could help confirm its secondary structure. In addition, it would be informative to determine whether the PP mutation alters the CD profile.

Does the N-terminal 1–11 region of Cep63 contribute to the formation of the high-molecular-weight Cep57(63–120)–Cep63(1–87) complex?

The electron-density map corresponding to the interacting region of the Cep57–Cep63 complex is not shown. Including this map would help assess the quality of the structural model and the confidence of the assigned interface.

Figure 6. In the invitro reconstitution assay, It would be important to determine whether full-length Cep57 behaves similarly under these conditions. Given that full-length Cep57 associates with MTs, does the exon 3 region remain essential for Cep63/Cep152 recruitment in the presence of MTs? Clarifying whether exon 3–dependent assembly is preserved in the context of the full-length protein would strengthen the physiological relevance of this experiment.

Figure 6. Do the ectopic assemblies associate with MTs? Given that the pull-down assay suggests stronger interaction with MTs than with acetylated MTs, it would be helpful for the authors to clarify whether the ectopic structures colocalize with or depend on MTs.

The manuscript suggests that complete loss of the Cep57–Cep63 interaction due to the Δexon 3 mutation is required for premature centriole disengagement and centriole overduplication. Could the authors provide additional evidence supporting this conclusion?

In the Discussion, the statement “Unlike the dimeric Cep57L1, the α-helix (301–316)-containing Cep57 functions as…” may benefit from clarification. Although the Cep57L1-C fragment has been shown to form a dimer, there is no direct evidence demonstrating that full-length Cep57L1 exists exclusively as a dimer, particularly given that it contains additional coiled-coil domains that may contribute to higher-order oligomerization. The authors may wish to temper this statement or clarify the experimental basis for this comparison.

A recent study demonstrated that NuSAP mediates the centrosomal recruitment of Cep57 (PMID: 41616107). This finding should be incorporated into the Discussion and considered alongside the present structural and functional data. Notably, the NuSAP-binding site on Cep57 overlaps with the Cep63-binding interface, suggesting potential competition or regulatory coordination between these interactions. This overlap may further clarify the hierarchical organization and regulatory control of Cep57 within the PCM assembly pathway.

(Remarks on code availability)
I am not an expert in MINFLUX microscopy.

Reviewer #3

(Remarks to the Author)
Ahn, Park and co-workers present a detailed study providing advanced molecular insights into the role of Cep57 in organizing inner pericentriolar material assemblies. This reviewer has focused on evaluation of the high-resolution microscopy aspects in this article, in particular the MINFLUX super-resolution experiments and associated data processing strategies.

The key utility of MINFLUX in this work is related to its comparatively high resolution along the optic axis (z), which allows to accurately map protein organizations and quantify confined spatial regions such as Cep57 association with acetylated tubulin for tilted orientation of the centrioles. The MINFLUX imaging of proteins labeled by indirect immunofluorescence in fixed cells, with dark-state blinking of suitable organic dyes in thiol-containing buffer, is described in sufficient detail in terms of choices of labelling, acquisition and processing.

The authors should clarify the following aspects in a revision of their manuscript:

The fact that the proximal ends of Cep57 and acetylated tubulin localization sets were visually leveled with respect to each other (Fig. 1c) should be clearly stated in the main text, and the rationale for this choice made more clear by the authors. Perhaps other data corroborates this? Technically, it would appear that a displacement from the proximal end cannot be excluded based on independent microscopy recordings, in the absence of direct co-registration of both targets.

On p. 7, l. 2ff, it is asserted based on the corresponding data that the PP mutant exhibited sparser localizations and fewer

large clusters compared to the WT, both of which is apparent, and that these were “scattered over a substantially expanded PCM space”. The latter conclusion should be more carefully derived, including why the criterion of “90% of all localizations” to define the spatial region is suitable. At generally much lower concentration, and with some potentially unspecific contributions, isn’t the finding actually that of a certainly less prominent clustering and generally lower protein concentration? Perhaps this is explained elsewhere, but what is the significance of the expanded space? The authors may expand on this point.

The authors should also clearly state if the comparisons were made for recordings of similar durations and similar imaging conditions, as would be adequate.

The MINFLUX imaging sequence(s) used should be stated in the Supplementary Information.
The following comments should further be taken into consideration to improve the clarity of arguments, accuracy and readability:

p. 4, l. 16: suggest to delete “at high spatio-temporal resolution”. The aspect of following a protein or proteins in time is not realized here and this early statement in the Results section could be somewhat misleading.

p. 4, l. 15: a reference to three-dimensional implementation of MINFLUX should additionally include Gwosch, Pape, Balzarotti et al. Nature Methods 2020

p. 4, l. 21: suggest to delete “of applications with noise”

p. 4, l. 27: suggest to delete “using MATLAB” (procedures are described separately in the Methods)

p. 4, l. 32: suggest to reword to: “the stochastic nature of sampling fluorophores” for clarity

p. 4, l. 38; suggest to replace “coexist” by “are found in closest proximity when concentrically aligned”

p. 4, l. 47: “but marginally stricter” should be improved to convey the exact meaning, the wording could be more precise.

p. 6, l. 45: suggest to delete “respective”

p. 25, l. 28: the correct reference for the used instrument is to Schmidt et al. Nature Communications 2021.

p. 26, l. 5f: a suitable reference for the applied scaling in z is to Gwosch, Pape, Balzarotti et al. Nature Methods 2020 (already cited elsewhere; the other existing reference is not specific to this subject)

p. 26, l. 20: suggest to reword to “trace-based (i.e., requiring at least 5 localizations in a trace)” if correct

p. 26, l. 37ff: please motivate the use of 200 and 500 nm heights of the rods in the alignment template. Perhaps it could be useful to state clearly at the outset here, or also in the main text along with the first description of MINFLUX imaging results, that the localization sets did not extend further out in the “height” dimension.

Fig. 1d: The authors should clarify what the “95%” designation refers to.

Extended Data Fig. 1e bottom: What is the significance of the label “Trace length 17.06”? Please clarify.

(Remarks on code availability)

Version 1:

Reviewer comments:

Reviewer #2

(Remarks to the Author)
The authors have adequately addressed all of my comments. The revised manuscript has been significantly improved, including the complete revision of the abstract, and I have no further questions.

(Remarks on code availability)

Reviewer #3

(Remarks to the Author)
The authors have fully addressed the points raised by this reviewer and improved the manuscript accordingly.

(Remarks on code availability)

Open Access This Peer Review File is licensed under a Creative Commons Attribution 4.0 International License, which permits use, sharing, adaptation, distribution and reproduction in any medium or format, as long as you give appropriate credit to the original author(s) and the source, provide a link to the Creative Commons license, and indicate if changes were made.
In cases where reviewers are anonymous, credit should be given to 'Anonymous Referee' and the source.
The images or other third party material in this Peer Review File are included in the article's Creative Commons license, unless indicated otherwise in a credit line to the material. If material is not included in the article's Creative Commons license and your intended use is not permitted by statutory regulation or exceeds the permitted use, you will need to obtain permission directly from the copyright holder.
To view a copy of this license, visit https://creativecommons.org/licenses/by/4.0/

Below are copies of three reviewers' comments and our point-by-point responses.

REVIEWER COMMENTS

Reviewer #1 (Remarks to the Author):

This is an excellent paper detailing interactions between Cep63 and Cep57(Cep192) and Cep152 that brings new insight into the control of centriole duplication. The study maps the interaction between Cep57 and microtubules and also with Cep63. The region interacting with Cep63 is deleted in the mosaic variegated aneuploidy (MVA) genetic disorder characterized by chromosome missegregation. Cep63 also binds Cep192, the partner of Plk4 that drives centriole duplication. I have no concerns about the data and I believe that the body of the paper is acceptable for publication without needing any modification. The only change I would suggest to the authors is to re-write their abstract to make it more widely accessible. This would mean removing the detailed description of precise defects and giving a description of the molecular interactions they have uncovered and ther importance for recruiting Plk4 and peri-centriolar material. In other words those features that lead to the model presented in Fig. 7.

Response: As this reviewer suggested, we re-wrote the abstract to improve readability for a wider audience. Thank you for the suggestion.

Reviewer #2 (Remarks to the Author):

This work consolidates and extends current understanding of PCM organization by providing structural insight into how Cep57 integrates into the Cep63–Cep152 scaffold through a combination of super-resolution imaging, structural biology, and biochemical reconstitution. The study identifies a helical region (residues 301–316) of Cep57 that is critical for Cep57-C oligomerization, microtubule binding, and centrosomal localization. In addition, it defines the binding interface of the Cep57–Cep63 complex. Functional mutagenesis further demonstrates that this interaction is essential for the stability of the Cep57–Cep63–Cep152 structural platform and for Plk4-dependent centriole duplication. These findings provide a mechanistic explanation for how exon 3 deletion in Cep57 disrupts this structural axis, thereby linking genetic observations in MVA2 syndrome to defects in PCM organization. However, rather than introducing a fundamentally new conceptual framework, this study builds upon established models of centrosomal architecture and centriole duplication by defining molecular interfaces and clarifying the architectural hierarchy that stabilizes PCM attachment to centriolar microtubules.

Overall, the work represents a careful and meaningful refinement of the current centrosome assembly model (PMID: 32152252, PMID: 32503940, PMID: 30858376; PMID: 37707473), adding structural resolution and disease relevance. While it provides valuable mechanistic detail, its advances are incremental in nature and may be better suited for a specialized journal with a focused readership in centrosome and cell division biology.

COMMENTS

Figure 2a. Cep57 appears to bind more strongly to MTs than to acetylated MTs. Could the authors comment on the possible basis for this difference? If this effect is related to MT dynamics or stability, it would be helpful to clarify whether Taxol treatment alters this binding behavior, for example by performing MT pelleting experiments.

Response: It appears that our data presentation may not have been sufficiently clear, which may have caused some confusion. First, the original Fig 2a (now moved to the supplementary Fig. 2f,

left), along with two additional independently obtained data (provided in Supplementary Fig. 2f, right, and in the Source data file), showed that Cep57 binds to purified tubulins (Note not MTs!) more strongly than Cep57L1 does. The new Fig. 2a (left), generated by combining three independently performed tubulin-binding analyses, shows that Cep57 binds to tubulins significantly better than Cep57L1. Similar results were obtained when acetylated tubulins were used (Fig. 2a). Second, to closely examine whether tubulin acetylation alters Cep57 binding, we performed comparative MBP-Cep57-C pulldown experiments in the tubulin buffer (80 mM PIPES, pH 6.9, 2 mM $MgCl_2$, 0.5 mM EGTA) at 4 °C (Supplementary Fig. 2g). However, we found that Cep57 binding to tubulin is unaffected by acetylation. This new finding is stated on page 5, line 31.

Also, we performed two additional independent experiments in the presence of 1 mM GTP, which showed that GTP did not noticeably alter their tubulin-binding levels. Data quantified from three (no GTP) and two (1 mM GTP) independent experiments are summarized in Fig. 2a (graphs).

The suggested MT pelleting experiment with Taxol treatment is shown in Fig. 2b (for tubulin) and Supplementary Fig. 2h (for acetylated tubulin). As the reviewer commented, MT co-sedimentation by Cep57-C was more effective when the assay was performed with unacetylated rather than acetylated tubulin. The inefficient co-sedimentation of acetylated MTs could be attributable to a slower nucleation and faster shrinkage kinetics of acetylated tubulin, as reported previously (Portran D, et al., Nat Cell Biol, 2017, PMID: 28250419). This is stated on page 5, line 35.

Figure 2c. It would be helpful to include control experiments using MBP–Cep57-C alone to clarify that the observed absorbance signal reflects MT polymerization rather than potential aggregation of Cep57 (Fig. 2f). This would strengthen confidence in the interpretation of the assay.

Response: As suggested, we have now provided the MBP-Cep57-C-alone control, which shows a small increase in turbidity over time. This level of turbidity increase by the protein itself should not significantly contribute to the robust turbidity induced by MBP-Cep57-C-mediated MT polymerization.

Figure 2e. MTs appear more curved in the presence of CEP57-C. Could the authors clarify what might account for this increased curvature?

Response: As the reviewer pointed out, bundled MTs indeed showed a mixture of curved and straight MTs. To avoid misinterpretation and improve clarity for readers, we have now provided a new image along with additional images provided in Supplementary Fig. 2i. The reason for generating the curved MT bundles remains unknown.

The authors state that Cep57 (301–316) adopts a helical conformation. It would be helpful to provide direct structural evidence supporting this claim. For example, circular dichroism analysis of this fragment without the MBP tag could help confirm its secondary structure. In addition, it would be informative to determine whether the PP mutation alters the CD profile.

Response: As requested, we purified MBP-untagged Cep57 (277–334) and its PP mutant and performed circular dichroism analyses. The resulting data, showing absorbance and molar ellipticity, clearly demonstrate that the 277–334 region exhibits alpha-helicity and that the PP mutation disrupts this property (Supplementary Fig. 3a). The proteins used in the assays are shown on the right. These new results are described on page 6, line 36. As you may notice, the purity of Cep57 (277–334) is not as high as that of the PP mutant. Please note that purifying Cep57 (277–334) was particularly

challenging because it forms an oligomer and therefore co-elutes with the cleaved MBP tag. Its expression level was also very low. Various columns, including both anionic and cationic, were not helpful. Because this fragment is highly hydrophobic in the alpha-helix-forming (301–316) region, the MBP tag is necessary to improve its solubility. Not surprisingly, our multiple attempts to purify His-Cep57 (277–334) without the MBP tag were unsuccessful because of the fragment's strong tendency to self-associate and aggregate. The MBP-untagged Cep57 (277–334) WT and PP shown in Supplementary Fig. 3a (right) were the highest purity we could achieve.

To gain additional data that support the alpha-helical nature of Cep57 (277–334), we examined whether MBP-fused Cep57 (277–334) undergoes monomer/dimer–oligomer transition, a physicochemical process that is well characterized to be mediated by helix-helix interactions. The SEC and iSCAMS results provided in Supplementary Fig. 3b,c demonstrate that the oligomeric MBP-Cep57 (277–334) can be dissociated to yield the monomeric/dimeric forms upon dilution. The PP mutant remained as a monomer/dimer under all the conditions that we examined. These new results are provided on page 6, line 37. Consistent with this finding, sedimentation velocity analysis showed that MBP-Cep57 (277–334) sediments as a high MW species (Supplementary Fig. 2n).

Does the N-terminal 1–11 region of Cep63 contribute to the formation of the high-molecular-weight Cep57(63–120)–Cep63(1–87) complex?

Response: To address this question, we purified the Cep57(63–120)•Cep63(12–87) complex lacking the N-terminal 11 residues. This complex was not as tight as its parental Cep57(63–120)•Cep63(1–87) complex and therefore contained a significant degree of dimeric Cep57(63–120) that could not be eliminated during the purification. This suggests that the N-terminal Cep63 (1–11) region helps stabilize the Cep57•Cep63 complex.

Sedimentation velocity analysis revealed that Cep57(63–120)•Cep63(12–87) forms concentration-dependent, undefinable, large aggregates, which are detected as a broader peak of 4 S–6 S (Results are provided in Supplementary Fig. 4i). This finding suggests that the N-terminal Cep63 (1–11) motif may contribute to modulating the thermodynamic equilibrium of the Cep57–Cep63 interaction, allowing it to undergo the stepwise assembly of higher MW complexes. This is described on page 8, line 34, and also in the figure legend.

The electron-density map corresponding to the interacting region of the Cep57–Cep63 complex is not shown. Including this map would help assess the quality of the structural model and the confidence of the assigned interface.

Response: As suggested, the electron map, contoured at 1.0 σ, is now provided in Supplementary Fig. 4g.

Figure 6. In the invitro reconstitution assay, It would be important to determine whether full-length Cep57 behaves similarly under these conditions. Given that full-length Cep57 associates with MTs, does the exon 3 region remain essential for Cep63/Cep152 recruitment in the presence of MTs? Clarifying whether exon 3–dependent assembly is preserved in the context of the full-length protein would strengthen the physiological relevance of this experiment.

Response: As the reviewer noted, establishing an in vitro reconstitution assay using the full-length Cep57 would be important. Unfortunately, purification of the full-length Cep57 was not technically feasible because it is highly aggregated and prone to degradation. However, in our in vitro reconstitution, we view that using the C-terminal streptavidin fusion, which binds to biotinylated

nanospheres, could mimic the physical entity of the centriolar MT-bound Cep57 C-terminal domain. Under these conditions, the Cep57-N (1–120)-Halo-streptavidin fusion, containing the Cep63-binding 63–120, but not its Cep63-binding motif-deficient Cep57-N (1–67)-Halo-streptavidin form, recruits downstream Cep63 and Cep152 (as shown in Fig. 6i). This finding is consistent with the data obtained with the cell-based assay shown in Fig. 6h and Supplementary Fig. 6j that clearly demonstrated that recruitment of Cep63 and Cep152 to an SpoVM-assisted ectopic Cep57 assembly was exon 3-dependent.

Figure 6. Do the ectopic assemblies associate with MTs? Given that the pull-down assay suggests stronger interaction with MTs than with acetylated MTs, it would be helpful for the authors to clarify whether the ectopic structures colocalize with or depend on MTs.

Response: As suggested, we examined whether tubulin signals can be detectable at the ectopic assemblies under the same conditions as in Supplementary Fig. 6j. However, tubulin signals were not detectable under our imaging conditions. This finding is consistent with the view that Cep57 can work together with Cep63 and Cep152 to form an ectopic self-assembly. Cep63-Cep152 are demonstrated to form a self-assembly in vivo and in vitro (Kim, TS, et al., Nat Commun, 2019; PMID: 30858376). The finding in Supplementary Fig. 6j is stated on page 11, line 11.

The manuscript suggests that complete loss of the Cep57–Cep63 interaction due to the Δexon 3 mutation is required for premature centriole disengagement and centriole overduplication. Could the authors provide additional evidence supporting this conclusion?

Response: Multiple studies performed with RNAi-treated cultured cells and MVA2-patient-derived lymphoblastoid cell line cells (Ito K, et al., JCB, 2021, PMID: 33492359; Watanabe K, et al., Nat Commun, 2019, PMID: 30804344) and Cep57 knockout cells (Ito K, EMBO J, 2025, PMID: 39905228) demonstrated that depletion or loss of Cep57 or MVA2-linked p.L309Pfs*9 truncation mutant causes centriole disengagement and its ensuing centriole overduplication. Here, we showed that primary MVA2-linked T lymphocytes bearing the *exon 3*Δ mutation exhibit a rather predominant centriole-duplication defect, with only a low degree of centriole overduplication. As reported in the three papers listed above, centriole overduplication in various Cep57-compromised cells arises as a consequence of premature centriole disengagement. Notably, we found that Cep57 or Cep63 mutants attenuated in the Cep57–Cep63 interaction (e.g., Cep57 4A and 2K mutants in Fig. 5c) did not show any detectable level of centriole overduplication. Based on these findings, along with previous reports, we suggest that a complete loss of the Cep57–Cep63 interaction is likely required for inducing premature centriole disengagement-induced centriole overduplication. To incorporate the reviewer's comment, we toned down our statement on page 10, line 32, and also in the abstract, "likely resulting from disorganized PCM-induced premature centriole disengagement".

In the Discussion, the statement "Unlike the dimeric Cep57L1, the α-helix (301–316)-containing Cep57 functions as…" may benefit from clarification. Although the Cep57L1-C fragment has been shown to form a dimer, there is no direct evidence demonstrating that full-length Cep57L1 exists exclusively as a dimer, particularly given that it contains additional coiled-coil domains that may contribute to higher-order oligomerization. The authors may wish to temper this statement or clarify the experimental basis for this comparison.

Response: The reviewer is correct in that other parts of Cep57L1 can also engage in additional interactions. Therefore, we changed "Cep57L1" to "Cep57L1-C".

A recent study demonstrated that NuSAP mediates the centrosomal recruitment of Cep57 (PMID: 41616107). This finding should be incorporated into the Discussion and considered alongside the present structural and functional data. Notably, the NuSAP-binding site on Cep57 overlaps with the Cep63-binding interface, suggesting potential competition or regulatory coordination between these interactions. This overlap may further clarify the hierarchical organization and regulatory control of Cep57 within the PCM assembly pathway.

Response: We conducted competitive coimmunoprecipitation assays using transfected HEK293 cells. The results showed that NuSAP1 interacted with Cep57 only weakly when compared to Cep63. Moreover, it neither detectably inhibited nor promoted the Cep57–Cep63 interaction. These findings are described on page 12, line 38.

Reviewer #3 (Remarks to the Author):

Ahn, Park and co-workers present a detailed study providing advanced molecular insights into the role of Cep57 in organizing inner pericentriolar material assemblies. This reviewer has focused on evaluation of the high-resolution microscopy aspects in this article, in particular the MINFLUX super-resolution experiments and associated data processing strategies.

The key utility of MINFLUX in this work is related to its comparatively high resolution along the optic axis (z), which allows to accurately map protein organizations and quantify confined spatial regions such as Cep57 association with acetylated tubulin for tilted orientation of the centrioles. The MINFLUX imaging of proteins labeled by indirect immunofluorescence in fixed cells, with dark-state blinking of suitable organic dyes in thiol-containing buffer, is described in sufficient detail in terms of choices of labelling, acquisition and processing.

The authors should clarify the following aspects in a revision of their manuscript:

The fact that the proximal ends of Cep57 and acetylated tubulin localization sets were visually leveled with respect to each other (Fig. 1c) should be clearly stated in the main text, and the rationale for this choice made more clear by the authors. Perhaps other data corroborates this? Technically, it would appear that a displacement from the proximal end cannot be excluded based on independent microscopy recordings, in the absence of direct co-registration of both targets.

Response: As requested, visually leveling the Cep57 and acetylated tubulin signals and the rationale and justification of this choice are clearly stated in the main text (page 4, line 36). To help justify our proximal-end alignment process, we provided another 3D-SIM image showing the relative localization patterns of Cep57 and acetylated tubulin in Supplementary Fig. 1a. For clarity, our original description of the alignment process remains in the Methods section, with some modifications (see the 3D-MINFLUX image analysis section). Even though the two independently obtained maps of Cep57 and acetylated tubulin are merged, inevitably causing some degree of signal displacement between them (as the reviewer pointed out), it is evident that a significant fraction of Cep57 localizations are in close proximity to acetylated tubulin localizations in the PCM space (as shown in Fig. 1c). Therefore, we stated "in close proximity, showing considerable spatial coincidence" on page 4, line 41. Also, the potential concern regarding signal displacement in the merged image is mentioned on page 27, line 6.

On p. 7, l. 2ff, it is asserted based on the corresponding data that the PP mutant exhibited sparser

localizations and fewer large clusters compared to the WT, both of which is apparent, and that these were "scattered over a substantially expanded PCM space". The latter conclusion should be more carefully derived, including why the criterion of "90% of all localizations" to define the spatial region is suitable. At generally much lower concentration, and with some potentially unspecific contributions, isn't the finding actually that of a certainly less prominent clustering and generally lower protein concentration? Perhaps this is explained elsewhere, but what is the significance of the expanded space? The authors may expand on this point.

Response: As suggested, the justification for using the centrally localized 90% for comparative analysis, which we arbitrarily used to minimize the influence of sparsely distributed localization events, is stated on page 7, line 14. While the expression level of Cep57 WT and its PP mutant is similar, as shown in Fig. 3e, the PP mutant shows sparse distribution over a much larger space. Since the PP mutant exhibits markedly diminished MT binding and pericentriolar localization, we attribute this localization defect to its inability to form an oligomeric state (due to the lack of alpha-helicity in the 301–316 region). Consequently, cells expressing the PP mutant show a defect in centriole duplication, as shown in Fig. 3f and Supplementary Fig. 3e. These findings are all explained, where appropriate. However, as the reviewer pointed out, "scattered over a substantially expanded PCM space" could be misleading. Therefore, we changed "expanded PCM space", which could mean the mitotic expansion of the PCM, to "wider PCM region".

The authors should also clearly state if the comparisons were made for recordings of similar durations and similar imaging conditions, as would be adequate.

Response: The requested information is clearly provided on page 26, line 1.

The MINFLUX imaging sequence(s) used should be stated in the Supplementary Information.

Response: As requested, the imaging process and the condition of the MINFLUX buffer are described in detail in the 2nd paragraph of the Methods section: MINFLUX data acquisition.

The following comments should further be taken into consideration to improve the clarity of arguments, accuracy and readability:

p. 4, l. 16: suggest to delete "at high spatio-temporal resolution". The aspect of following a protein or proteins in time is not realized here and this early statement in the Results section could be somewhat misleading.

Response: The reviewer is correct. The wording is changed accordingly.

p. 4, l. 15: a reference to three-dimensional implementation of MINFLUX should additionally include Gwosch, Pape, Balzarotti et al. Nature Methods 2020

Response: This is taken care of. Thanks!

p. 4, l. 21: suggest to delete "of applications with noise"

Response: This is taken care of. Thanks!

p. 4, l. 27: suggest to delete "using MATLAB" (procedures are described separately in the Methods)

Response: This is taken care of. Thanks!

p. 4, l. 32: suggest to reword to: "the stochastic nature of sampling fluorophores" for clarity

Response: The wording is changed, as suggested. Thanks!

p. 4, l. 38; suggest to replace "coexist" by "are found in closest proximity when concentrically aligned"
Response: As suggested, we changed the wording accordingly. Thanks!

p. 4, l. 47: "but marginally stricter" should be improved to convey the exact meaning, the wording could be more precise.
Response: As suggested, we detailed the data and stated that, except for one centriole, all 11 counts were closely comparable between the 3-nm cutoff and the 5-localization cutoff filters (page 4, line 50).

p. 6, l. 45: suggest to delete "respective"
Response: This is taken care of. Thanks!

p. 25, l. 28: the correct reference for the used instrument is to Schmidt et al. Nature Communications 2021.
Response: This is taken care of. Thanks!

p. 26, l. 5f: a suitable reference for the applied scaling in z is to Gwosch, Pape, Balzarotti et al. Nature Methods 2020 (already cited elsewhere; the other existing reference is not specific to this subject)
Response: This is taken care of. Thanks!

p. 26, l. 20: suggest to reword to "trace-based (i.e., requiring at least 5 localizations in a trace)" if correct
Response: This is taken care of. Thanks!

p. 26, l. 37ff: please motivate the use of 200 and 500 nm heights of the rods in the alignment template. Perhaps it could be useful to state clearly at the outset here, or also in the main text along with the first description of MINFLUX imaging results, that the localization sets did not extend further out in the "height" dimension.
Response: As suggested, the detailed reasons for using 200-nm- and 500-nm-tall rods in the alignment templates for acetylated tubulins and Cep57 signals, respectively, are provided on page 26, line 42.

Fig. 1d: The authors should clarify what the "95%" designation refers to.
Response: We have now made it clear that 95% of the total Cep57 signals, excluding the 5% outliers, were used for Fig. 1d, and stated this on page 27 in the Calculation of the volume of Cep57 localization section.

Extended Data Fig. 1e bottom: What is the significance of the label "Trace length 17.06"? Please clarify.
Response: The number was our average trace length, and it was entered into the figure by accident. We took it out now.

Below are copies of three reviewers' comments and our point-by-point responses.

REVIEWER COMMENTS

Reviewer #1 (Remarks to the Author):

This is an excellent paper detailing interactions between Cep63 and Cep57(Cep192) and Cep152 that brings new insight into the control of centriole duplication. The study maps the interaction between Cep57 and microtubules and also with Cep63. The region interacting with Cep63 is deleted in the mosaic variegated aneuploidy (MVA) genetic disorder characterized by chromosome missegregation. Cep63 also binds Cep192, the partner of Plk4 that drives centriole duplication. I have no concerns about the data and I believe that the body of the paper is acceptable for publication without needing any modification. The only change I would suggest to the authors is to re-write their abstract to make it more widely accessible. This would mean removing the detailed description of precise defects and giving a description of the molecular interactions they have uncovered and ther importance for recruiting Plk4 and peri-centriolar material. In other words those features that lead to the model presented in Fig. 7.

Response: As this reviewer suggested, we re-wrote the abstract to improve readability for a wider audience. Thank you for the suggestion.

Reviewer #2 (Remarks to the Author):

This work consolidates and extends current understanding of PCM organization by providing structural insight into how Cep57 integrates into the Cep63–Cep152 scaffold through a combination of super-resolution imaging, structural biology, and biochemical reconstitution. The study identifies a helical region (residues 301–316) of Cep57 that is critical for Cep57-C oligomerization, microtubule binding, and centrosomal localization. In addition, it defines the binding interface of the Cep57–Cep63 complex. Functional mutagenesis further demonstrates that this interaction is essential for the stability of the Cep57–Cep63–Cep152 structural platform and for Plk4-dependent centriole duplication. These findings provide a mechanistic explanation for how exon 3 deletion in Cep57 disrupts this structural axis, thereby linking genetic observations in MVA2 syndrome to defects in PCM organization. However, rather than introducing a fundamentally new conceptual framework, this study builds upon established models of centrosomal architecture and centriole duplication by defining molecular interfaces and clarifying the architectural hierarchy that stabilizes PCM attachment to centriolar microtubules.

Overall, the work represents a careful and meaningful refinement of the current centrosome assembly model (PMID: 32152252, PMID: 32503940, PMID: 30858376; PMID: 37707473), adding structural resolution and disease relevance. While it provides valuable mechanistic detail, its advances are incremental in nature and may be better suited for a specialized journal with a focused readership in centrosome and cell division biology.

COMMENTS

Figure 2a. Cep57 appears to bind more strongly to MTs than to acetylated MTs. Could the authors comment on the possible basis for this difference? If this effect is related to MT dynamics or stability, it would be helpful to clarify whether Taxol treatment alters this binding behavior, for example by performing MT pelleting experiments.

Response: It appears that our data presentation may not have been sufficiently clear, which may have caused some confusion. First, the original Fig 2a (now moved to the supplementary Fig. 2f,

left), along with two additional independently obtained data (provided in Supplementary Fig. 2f, right, and in the Source data file), showed that Cep57 binds to purified tubulins (Note not MTs!) more strongly than Cep57L1 does. The new Fig. 2a (left), generated by combining three independently performed tubulin-binding analyses, shows that Cep57 binds to tubulins significantly better than Cep57L1. Similar results were obtained when acetylated tubulins were used (Fig. 2a). Second, to closely examine whether tubulin acetylation alters Cep57 binding, we performed comparative MBP-Cep57-C pulldown experiments in the tubulin buffer (80 mM PIPES, pH 6.9, 2 mM $MgCl_2$, 0.5 mM EGTA) at 4 °C (Supplementary Fig. 2g). However, we found that Cep57 binding to tubulin is unaffected by acetylation. This new finding is stated on page 5, line 31.

Also, we performed two additional independent experiments in the presence of 1 mM GTP, which showed that GTP did not noticeably alter their tubulin-binding levels. Data quantified from three (no GTP) and two (1 mM GTP) independent experiments are summarized in Fig. 2a (graphs).

The suggested MT pelleting experiment with Taxol treatment is shown in Fig. 2b (for tubulin) and Supplementary Fig. 2h (for acetylated tubulin). As the reviewer commented, MT co-sedimentation by Cep57-C was more effective when the assay was performed with unacetylated rather than acetylated tubulin. The inefficient co-sedimentation of acetylated MTs could be attributable to a slower nucleation and faster shrinkage kinetics of acetylated tubulin, as reported previously (Portran D, et al., Nat Cell Biol, 2017, PMID: 28250419). This is stated on page 5, line 35.

Figure 2c. It would be helpful to include control experiments using MBP–Cep57-C alone to clarify that the observed absorbance signal reflects MT polymerization rather than potential aggregation of Cep57 (Fig. 2f). This would strengthen confidence in the interpretation of the assay.

Response: As suggested, we have now provided the MBP-Cep57-C-alone control, which shows a small increase in turbidity over time. This level of turbidity increase by the protein itself should not significantly contribute to the robust turbidity induced by MBP-Cep57-C-mediated MT polymerization.

Figure 2e. MTs appear more curved in the presence of CEP57-C. Could the authors clarify what might account for this increased curvature?

Response: As the reviewer pointed out, bundled MTs indeed showed a mixture of curved and straight MTs. To avoid misinterpretation and improve clarity for readers, we have now provided a new image along with additional images provided in Supplementary Fig. 2i. The reason for generating the curved MT bundles remains unknown.

The authors state that Cep57 (301–316) adopts a helical conformation. It would be helpful to provide direct structural evidence supporting this claim. For example, circular dichroism analysis of this fragment without the MBP tag could help confirm its secondary structure. In addition, it would be informative to determine whether the PP mutation alters the CD profile.

Response: As requested, we purified MBP-untagged Cep57 (277–334) and its PP mutant and performed circular dichroism analyses. The resulting data, showing absorbance and molar ellipticity, clearly demonstrate that the 277–334 region exhibits alpha-helicity and that the PP mutation disrupts this property (Supplementary Fig. 3a). The proteins used in the assays are shown on the right. These new results are described on page 6, line 36. As you may notice, the purity of Cep57 (277–334) is not as high as that of the PP mutant. Please note that purifying Cep57 (277–334) was particularly

challenging because it forms an oligomer and therefore co-elutes with the cleaved MBP tag. Its expression level was also very low. Various columns, including both anionic and cationic, were not helpful. Because this fragment is highly hydrophobic in the alpha-helix-forming (301–316) region, the MBP tag is necessary to improve its solubility. Not surprisingly, our multiple attempts to purify His-Cep57 (277–334) without the MBP tag were unsuccessful because of the fragment's strong tendency to self-associate and aggregate. The MBP-untagged Cep57 (277–334) WT and PP shown in Supplementary Fig. 3a (right) were the highest purity we could achieve.

To gain additional data that support the alpha-helical nature of Cep57 (277–334), we examined whether MBP-fused Cep57 (277–334) undergoes monomer/dimer–oligomer transition, a physicochemical process that is well characterized to be mediated by helix-helix interactions. The SEC and iSCAMS results provided in Supplementary Fig. 3b,c demonstrate that the oligomeric MBP-Cep57 (277–334) can be dissociated to yield the monomeric/dimeric forms upon dilution. The PP mutant remained as a monomer/dimer under all the conditions that we examined. These new results are provided on page 6, line 37. Consistent with this finding, sedimentation velocity analysis showed that MBP-Cep57 (277–334) sediments as a high MW species (Supplementary Fig. 2n).

Does the N-terminal 1–11 region of Cep63 contribute to the formation of the high-molecular-weight Cep57(63–120)–Cep63(1–87) complex?

Response: To address this question, we purified the Cep57(63–120)•Cep63(12–87) complex lacking the N-terminal 11 residues. This complex was not as tight as its parental Cep57(63–120)•Cep63(1–87) complex and therefore contained a significant degree of dimeric Cep57(63–120) that could not be eliminated during the purification. This suggests that the N-terminal Cep63 (1–11) region helps stabilize the Cep57•Cep63 complex.

Sedimentation velocity analysis revealed that Cep57(63–120)•Cep63(12–87) forms concentration-dependent, undefinable, large aggregates, which are detected as a broader peak of 4 S–6 S (Results are provided in Supplementary Fig. 4i). This finding suggests that the N-terminal Cep63 (1–11) motif may contribute to modulating the thermodynamic equilibrium of the Cep57–Cep63 interaction, allowing it to undergo the stepwise assembly of higher MW complexes. This is described on page 8, line 34, and also in the figure legend.

The electron-density map corresponding to the interacting region of the Cep57–Cep63 complex is not shown. Including this map would help assess the quality of the structural model and the confidence of the assigned interface.

Response: As suggested, the electron map, contoured at 1.0 σ, is now provided in Supplementary Fig. 4g.

Figure 6. In the invitro reconstitution assay, It would be important to determine whether full-length Cep57 behaves similarly under these conditions. Given that full-length Cep57 associates with MTs, does the exon 3 region remain essential for Cep63/Cep152 recruitment in the presence of MTs? Clarifying whether exon 3–dependent assembly is preserved in the context of the full-length protein would strengthen the physiological relevance of this experiment.

Response: As the reviewer noted, establishing an in vitro reconstitution assay using the full-length Cep57 would be important. Unfortunately, purification of the full-length Cep57 was not technically feasible because it is highly aggregated and prone to degradation. However, in our in vitro reconstitution, we view that using the C-terminal streptavidin fusion, which binds to biotinylated

nanospheres, could mimic the physical entity of the centriolar MT-bound Cep57 C-terminal domain. Under these conditions, the Cep57-N (1–120)-Halo-streptavidin fusion, containing the Cep63-binding 63–120, but not its Cep63-binding motif-deficient Cep57-N (1–67)-Halo-streptavidin form, recruits downstream Cep63 and Cep152 (as shown in Fig. 6i). This finding is consistent with the data obtained with the cell-based assay shown in Fig. 6h and Supplementary Fig. 6j that clearly demonstrated that recruitment of Cep63 and Cep152 to an SpoVM-assisted ectopic Cep57 assembly was exon 3-dependent.

Figure 6. Do the ectopic assemblies associate with MTs? Given that the pull-down assay suggests stronger interaction with MTs than with acetylated MTs, it would be helpful for the authors to clarify whether the ectopic structures colocalize with or depend on MTs.

Response: As suggested, we examined whether tubulin signals can be detectable at the ectopic assemblies under the same conditions as in Supplementary Fig. 6j. However, tubulin signals were not detectable under our imaging conditions. This finding is consistent with the view that Cep57 can work together with Cep63 and Cep152 to form an ectopic self-assembly. Cep63-Cep152 are demonstrated to form a self-assembly in vivo and in vitro (Kim, TS, et al., Nat Commun, 2019; PMID: 30858376). The finding in Supplementary Fig. 6j is stated on page 11, line 11.

The manuscript suggests that complete loss of the Cep57–Cep63 interaction due to the Δexon 3 mutation is required for premature centriole disengagement and centriole overduplication. Could the authors provide additional evidence supporting this conclusion?

Response: Multiple studies performed with RNAi-treated cultured cells and MVA2-patient-derived lymphoblastoid cell line cells (Ito K, et al., JCB, 2021, PMID: 33492359; Watanabe K, et al., Nat Commun, 2019, PMID: 30804344) and Cep57 knockout cells (Ito K, EMBO J, 2025, PMID: 39905228) demonstrated that depletion or loss of Cep57 or MVA2-linked p.L309Pfs*9 truncation mutant causes centriole disengagement and its ensuing centriole overduplication. Here, we showed that primary MVA2-linked T lymphocytes bearing the *exon 3*Δ mutation exhibit a rather predominant centriole-duplication defect, with only a low degree of centriole overduplication. As reported in the three papers listed above, centriole overduplication in various Cep57-compromised cells arises as a consequence of premature centriole disengagement. Notably, we found that Cep57 or Cep63 mutants attenuated in the Cep57–Cep63 interaction (e.g., Cep57 4A and 2K mutants in Fig. 5c) did not show any detectable level of centriole overduplication. Based on these findings, along with previous reports, we suggest that a complete loss of the Cep57–Cep63 interaction is likely required for inducing premature centriole disengagement-induced centriole overduplication. To incorporate the reviewer's comment, we toned down our statement on page 10, line 32, and also in the abstract, "likely resulting from disorganized PCM-induced premature centriole disengagement".

In the Discussion, the statement "Unlike the dimeric Cep57L1, the α-helix (301–316)-containing Cep57 functions as…" may benefit from clarification. Although the Cep57L1-C fragment has been shown to form a dimer, there is no direct evidence demonstrating that full-length Cep57L1 exists exclusively as a dimer, particularly given that it contains additional coiled-coil domains that may contribute to higher-order oligomerization. The authors may wish to temper this statement or clarify the experimental basis for this comparison.

Response: The reviewer is correct in that other parts of Cep57L1 can also engage in additional interactions. Therefore, we changed "Cep57L1" to "Cep57L1-C".

A recent study demonstrated that NuSAP mediates the centrosomal recruitment of Cep57 (PMID: 41616107). This finding should be incorporated into the Discussion and considered alongside the present structural and functional data. Notably, the NuSAP-binding site on Cep57 overlaps with the Cep63-binding interface, suggesting potential competition or regulatory coordination between these interactions. This overlap may further clarify the hierarchical organization and regulatory control of Cep57 within the PCM assembly pathway.

Response: We conducted competitive coimmunoprecipitation assays using transfected HEK293 cells. The results showed that NuSAP1 interacted with Cep57 only weakly when compared to Cep63. Moreover, it neither detectably inhibited nor promoted the Cep57–Cep63 interaction. These findings are described on page 12, line 38.

Reviewer #3 (Remarks to the Author):

Ahn, Park and co-workers present a detailed study providing advanced molecular insights into the role of Cep57 in organizing inner pericentriolar material assemblies. This reviewer has focused on evaluation of the high-resolution microscopy aspects in this article, in particular the MINFLUX super-resolution experiments and associated data processing strategies.

The key utility of MINFLUX in this work is related to its comparatively high resolution along the optic axis (z), which allows to accurately map protein organizations and quantify confined spatial regions such as Cep57 association with acetylated tubulin for tilted orientation of the centrioles. The MINFLUX imaging of proteins labeled by indirect immunofluorescence in fixed cells, with dark-state blinking of suitable organic dyes in thiol-containing buffer, is described in sufficient detail in terms of choices of labelling, acquisition and processing.

The authors should clarify the following aspects in a revision of their manuscript:

The fact that the proximal ends of Cep57 and acetylated tubulin localization sets were visually leveled with respect to each other (Fig. 1c) should be clearly stated in the main text, and the rationale for this choice made more clear by the authors. Perhaps other data corroborates this? Technically, it would appear that a displacement from the proximal end cannot be excluded based on independent microscopy recordings, in the absence of direct co-registration of both targets.

Response: As requested, visually leveling the Cep57 and acetylated tubulin signals and the rationale and justification of this choice are clearly stated in the main text (page 4, line 36). To help justify our proximal-end alignment process, we provided another 3D-SIM image showing the relative localization patterns of Cep57 and acetylated tubulin in Supplementary Fig. 1a. For clarity, our original description of the alignment process remains in the Methods section, with some modifications (see the 3D-MINFLUX image analysis section). Even though the two independently obtained maps of Cep57 and acetylated tubulin are merged, inevitably causing some degree of signal displacement between them (as the reviewer pointed out), it is evident that a significant fraction of Cep57 localizations are in close proximity to acetylated tubulin localizations in the PCM space (as shown in Fig. 1c). Therefore, we stated “in close proximity, showing considerable spatial coincidence” on page 4, line 41. Also, the potential concern regarding signal displacement in the merged image is mentioned on page 27, line 6.

On p. 7, l. 2ff, it is asserted based on the corresponding data that the PP mutant exhibited sparser

localizations and fewer large clusters compared to the WT, both of which is apparent, and that these were "scattered over a substantially expanded PCM space". The latter conclusion should be more carefully derived, including why the criterion of "90% of all localizations" to define the spatial region is suitable. At generally much lower concentration, and with some potentially unspecific contributions, isn't the finding actually that of a certainly less prominent clustering and generally lower protein concentration? Perhaps this is explained elsewhere, but what is the significance of the expanded space? The authors may expand on this point.

Response: As suggested, the justification for using the centrally localized 90% for comparative analysis, which we arbitrarily used to minimize the influence of sparsely distributed localization events, is stated on page 7, line 14. While the expression level of Cep57 WT and its PP mutant is similar, as shown in Fig. 3e, the PP mutant shows sparse distribution over a much larger space. Since the PP mutant exhibits markedly diminished MT binding and pericentriolar localization, we attribute this localization defect to its inability to form an oligomeric state (due to the lack of alpha-helicity in the 301–316 region). Consequently, cells expressing the PP mutant show a defect in centriole duplication, as shown in Fig. 3f and Supplementary Fig. 3e. These findings are all explained, where appropriate. However, as the reviewer pointed out, "scattered over a substantially expanded PCM space" could be misleading. Therefore, we changed "expanded PCM space", which could mean the mitotic expansion of the PCM, to "wider PCM region".

The authors should also clearly state if the comparisons were made for recordings of similar durations and similar imaging conditions, as would be adequate.

Response: The requested information is clearly provided on page 26, line 1.

The MINFLUX imaging sequence(s) used should be stated in the Supplementary Information.

Response: As requested, the imaging process and the condition of the MINFLUX buffer are described in detail in the 2nd paragraph of the Methods section: MINFLUX data acquisition.

The following comments should further be taken into consideration to improve the clarity of arguments, accuracy and readability:

p. 4, l. 16: suggest to delete "at high spatio-temporal resolution". The aspect of following a protein or proteins in time is not realized here and this early statement in the Results section could be somewhat misleading.

Response: The reviewer is correct. The wording is changed accordingly.

p. 4, l. 15: a reference to three-dimensional implementation of MINFLUX should additionally include Gwosch, Pape, Balzarotti et al. Nature Methods 2020

Response: This is taken care of. Thanks!

p. 4, l. 21: suggest to delete "of applications with noise"

Response: This is taken care of. Thanks!

p. 4, l. 27: suggest to delete "using MATLAB" (procedures are described separately in the Methods)

Response: This is taken care of. Thanks!

p. 4, l. 32: suggest to reword to: "the stochastic nature of sampling fluorophores" for clarity

Response: The wording is changed, as suggested. Thanks!

p. 4, l. 38; suggest to replace "coexist" by "are found in closest proximity when concentrically aligned"
Response: As suggested, we changed the wording accordingly. Thanks!

p. 4, l. 47: "but marginally stricter" should be improved to convey the exact meaning, the wording could be more precise.
Response: As suggested, we detailed the data and stated that, except for one centriole, all 11 counts were closely comparable between the 3-nm cutoff and the 5-localization cutoff filters (page 4, line 50).

p. 6, l. 45: suggest to delete "respective"
Response: This is taken care of. Thanks!

p. 25, l. 28: the correct reference for the used instrument is to Schmidt et al. Nature Communications 2021.
Response: This is taken care of. Thanks!

p. 26, l. 5f: a suitable reference for the applied scaling in z is to Gwosch, Pape, Balzarotti et al. Nature Methods 2020 (already cited elsewhere; the other existing reference is not specific to this subject)
Response: This is taken care of. Thanks!

p. 26, l. 20: suggest to reword to "trace-based (i.e., requiring at least 5 localizations in a trace)" if correct
Response: This is taken care of. Thanks!

p. 26, l. 37ff: please motivate the use of 200 and 500 nm heights of the rods in the alignment template. Perhaps it could be useful to state clearly at the outset here, or also in the main text along with the first description of MINFLUX imaging results, that the localization sets did not extend further out in the "height" dimension.
Response: As suggested, the detailed reasons for using 200-nm- and 500-nm-tall rods in the alignment templates for acetylated tubulins and Cep57 signals, respectively, are provided on page 26, line 42.

Fig. 1d: The authors should clarify what the "95%" designation refers to.
Response: We have now made it clear that 95% of the total Cep57 signals, excluding the 5% outliers, were used for Fig. 1d, and stated this on page 27 in the Calculation of the volume of Cep57 localization section.

Extended Data Fig. 1e bottom: What is the significance of the label "Trace length 17.06"? Please clarify.
Response: The number was our average trace length, and it was entered into the figure by accident. We took it out now.
