# Supplementary material for: Cep57 organizes pericentriolar material through dual architectural modules and prevents human mosaic variegated aneuploidy
Source: Nat Commun. 2026 Sep 30;17:11262. doi: 10.1038/s41467-026-77795-4 (PMC13720213; doi:10.1038/s41467-026-77795-4)
Supplement: Supplementary file 3 [file 41467_2026_77795_MOESM3_ESM.pdf]

nature portfolio

Corresponding author(s): Kyung S. Lee

Last updated by author(s): August 4, 2026

Reporting Summary

Nature Portfolio wishes to improve the reproducibility of the work that we publish. This form provides structure for consistency and transparency in reporting. For further information on Nature Portfolio policies, see our Editorial Policies and the Editorial Policy Checklist.

Please do not complete any field with "not applicable" or n/a. Refer to the help text for what text to use if an item is not relevant to your study.
For final submission: please carefully check your responses for accuracy; you will not be able to make changes later.

Statistics

For all statistical analyses, confirm that the following items are present in the figure legend, table legend, main text, or Methods section.

n/a	Confirmed	
☐	☑	The exact sample size (*n*) for each experimental group/condition, given as a discrete number and unit of measurement
☐	☑	A statement on whether measurements were taken from distinct samples or whether the same sample was measured repeatedly
☐	☑	The statistical test(s) used AND whether they are one- or two-sided<br>*Only common tests should be described solely by name; describe more complex techniques in the Methods section.*
☐	☑	A description of all covariates tested
☐	☑	A description of any assumptions or corrections, such as tests of normality and adjustment for multiple comparisons
☐	☑	A full description of the statistical parameters including central tendency (e.g. means) or other basic estimates (e.g. regression coefficient) AND variation (e.g. standard deviation) or associated estimates of uncertainty (e.g. confidence intervals)
☐	☑	For null hypothesis testing, the test statistic (e.g. *F*, *t*, *r*) with confidence intervals, effect sizes, degrees of freedom and *P* value noted<br>*Give P values as exact values whenever suitable.*
☒	☐	For Bayesian analysis, information on the choice of priors and Markov chain Monte Carlo settings
☒	☐	For hierarchical and complex designs, identification of the appropriate level for tests and full reporting of outcomes
☒	☐	Estimates of effect sizes (e.g. Cohen's *d*, Pearson's *r*), indicating how they were calculated

Our web collection on statistics for biologists contains articles on many of the points above.

Software and code

Policy information about availability of computer code

Data collection	Abberior 3D-MINFLUX system, ZEISS ELYRA S.1 super-resolution microscope, ChemiDoc™ Imaging System (Bio-Rad Laboratories), Tecnai 12 120kV Electron Microscope with K2 Summit, ÄKTA pure chromatography system (Cytiva), Beckman Coulter ProteomeLab XL-I analytical ultracentrifuge, Beckman Optima XL-A analytical ultracentrifuge, Refeyn TwoMP mass photometer (Refeyn Ltd.), Agilent Technologies 1260 Infinity, Wyatt DAWN HELEOS II and OPTILAB T-rEX, 22-ID beamline at the Advanced Photon Source (Argonne, IL, USA), EnSpire Multimode Plate Reader (PerkinElmer, USA), Q100 autosampler, OneMP, MassFluidix HC
Data analysis	iMSPECTOR for MINFLUX, ZEN v2.1 (Zeiss), MATLAB (version R2022b), Image Lab, ImageJ (FIJI), UNICORN 7, SEDFIT, SEDNTERP, Anton Paar DMA 5000 density meter, Anton Paar AMVn rolling-ball viscometer, SEDPHAT, ASTRA (Wyatt, version 7.3.1), HKL2000, XDS software package, CCP4 Software Suite, Coot, PHENIX, PyMOL 3.1.8, GraphPad Prism 10, Chirascan Q100, AcquireMP, DiscoverMP

For manuscripts utilizing custom algorithms or software that are central to the research but not yet described in published literature, software must be made available to editors and reviewers. We strongly encourage code deposition in a community repository (e.g. GitHub). See the Nature Portfolio guidelines for submitting code & software for further information.

Data

Policy information about availability of data

All manuscripts must include a data availability statement. This statement should provide the following information, where applicable:

- Accession codes, unique identifiers, or web links for publicly available datasets
- A description of any restrictions on data availability
- For clinical datasets or third party data, please ensure that the statement adheres to our policy

The PDB accession codes for the two crystal structures in the manuscript are 21JQ and 9I7Q (PDB validation sheets are attached). Raw data are available upon request.

Research involving human participants, their data, or biological material

Policy information about studies with human participants or human data. See also policy information about sex, gender (identity/presentation), and sexual orientation and race, ethnicity and racism.

Reporting on sex and gender	The study included a father, his daughter, and his son. Sex or gender was not considered in the study design or analyses because it was not relevant to the research question. Primary T cells were purified and analyzed by immunostainings. Sex of the patients were described "son" and "daughter".
Reporting on race, ethnicity, or other socially relevant groupings	Race, ethnicity, and other socially relevant groupings were not collected or considered in this study, as these characteristics were not relevant to the study design or analyses.
Population characteristics	Heterozygous father, his daughter and son bearing homozygous mutations were the source of blood samples.
Recruitment	The participants were recruited as members of a family carrying the mutation of interest. Blood samples were obtained from the heterozygous father and his daughter and son, who were homozygous for the mutation. Participants were selected on the basis of their relevant genotype and familial relationship; therefore, the study population was not intended to be representative of the general population.
Ethics oversight	This study complied with all relevant ethical regulations. The study protocol was reviewed by the National Institutes of Health (NIH) Institutional Review Board (IRB; ID IRB002487), which determined that the research did not constitute human subjects research under DHHS and FDA regulations; therefore, IRB approval was not required. For studies involving primary T lymphocytes, all participants provided written informed consent, and the study was approved by the AIIMS Institute Ethics Committee (Approval No. IEC-342/06.07.2018, dated 30 July 2018).

Field-specific reporting

Please select the one below that is the best fit for your research. If you are not sure, read the appropriate sections before making your selection.

☑ Life sciences ☐ Behavioural & social sciences ☐ Ecological, evolutionary & environmental sciences

For a reference copy of the document with all sections, see nature.com/documents/nr-reporting-summary-flat.pdf

Life sciences study design

All studies must disclose on these points even when the disclosure is negative.

Sample size	The sample size was determined based on the magnitude of the standard deviation and the reproducibility of the data. For statistical significance, at least three independent experiments were performed except where otherwise indicated.
Data exclusions	No data were excluded.
Replication	All experiments were repeated at least three times, except where otherwise indicated. The results reliably support the conclusions stated in the manuscript.
Randomization	For X-ray crystal structure refinement and model validation, the data were randomly split into two groups. One group was used for refinement, and the other was used for validation. For sample quantification, imaging areas were randomly selected. Randomization of animals or participants was not relevant to this study. For the FRAP experiments, condensates from the in vivo and in vitro experiments were randomly selected for analysis.
Blinding	Image acquisition and quantification were conducted in a blinded manner.

Behavioural & social sciences study design

All studies must disclose on these points even when the disclosure is negative.

Study description	
Research sample	
Sampling strategy	
Data collection	
Timing	
Data exclusions	
Non-participation	
Randomization	

Ecological, evolutionary & environmental sciences study design

All studies must disclose on these points even when the disclosure is negative.

Study description

Research sample

Sampling strategy

Data collection

Timing and spatial scale

Data exclusions

Reproducibility

Randomization

Blinding

Did the study involve field work? ☐ Yes ☐ No

Field work, collection and transport

Field conditions

Location

Access & import/export

Disturbance

Reporting for specific materials, systems and methods

We require information from authors about some types of materials, experimental systems and methods used in many studies. Here, indicate whether each material, system or method listed is relevant to your study. If you are not sure if a list item applies to your research, read the appropriate section before selecting a response.

Materials & experimental systems

n/a	Involved in the study
☐	☑ Antibodies
☐	☑ Eukaryotic cell lines
☒	☐ Palaeontology and archaeology
☒	☐ Animals and other organisms
☒	☐ Clinical data
☒	☐ Dual use research of concern
☒	☐ Plants

Methods

n/a	Involved in the study
☒	☐ ChIP-seq
☒	☐ Flow cytometry
☒	☐ MRI-based neuroimaging

Antibodies

Antibodies used

All the antibodies used in this study are listed in the Supplementary Table 5 with Source information, catalog number. Previously published antibodies are referenced. All antibodies are available upon request.

Validation

Antibodies used for immunoprecipitation and immunoblotting were internally controlled within our results.
Antibodies used for immunofluorescence were either internally controlled or validated by siRNA. Commercially available antibodies were used according to manufacturer's recommendations.

Eukaryotic cell lines

Policy information about cell lines and Sex and Gender in Research

Cell line source(s)	HEK293T, U2OS were purchased. Primary T-lymphocytes were purified from healthy and MVA patients.
Authentication	Cells have been authenticated by the vendors (ATCC and Coriell).
Mycoplasma contamination	No mycoplasma contamination was found.
Commonly misidentified lines (See ICLAC register)	No commonly misidentified cell lines were used.

Palaeontology and Archaeology

Specimen provenance	
Specimen deposition	
Dating methods	

☐ Tick this box to confirm that the raw and calibrated dates are available in the paper or in Supplementary Information.

Ethics oversight	

Note that full information on the approval of the study protocol must also be provided in the manuscript.

Animals and other research organisms

Policy information about studies involving animals; ARRIVE guidelines recommended for reporting animal research, and Sex and Gender in Research

Laboratory animals	
Wild animals	
Reporting on sex	
Field-collected samples	
Ethics oversight	

Note that full information on the approval of the study protocol must also be provided in the manuscript.

Clinical data

Policy information about clinical studies

All manuscripts should comply with the ICMJE guidelines for publication of clinical research and a completed CONSORT checklist must be included with all submissions.

Clinical trial registration	
Study protocol	
Data collection	
Outcomes	

Dual use research of concern

Policy information about dual use research of concern

Hazards

Could the accidental, deliberate or reckless misuse of agents or technologies generated in the work, or the application of information presented in the manuscript, pose a threat to:

No | Yes

- [x] No [ ] Yes — Public health
- [x] No [ ] Yes — National security
- [x] No [ ] Yes — Crops and/or livestock
- [x] No [ ] Yes — Ecosystems
- [x] No [ ] Yes — Any other significant area

Experiments of concern

Does the work involve any of these experiments of concern:

No | Yes

- [x] No [ ] Yes — Demonstrate how to render a vaccine ineffective
- [x] No [ ] Yes — Confer resistance to therapeutically useful antibiotics or antiviral agents
- [x] No [ ] Yes — Enhance the virulence of a pathogen or render a nonpathogen virulent
- [x] No [ ] Yes — Increase transmissibility of a pathogen
- [x] No [ ] Yes — Alter the host range of a pathogen
- [x] No [ ] Yes — Enable evasion of diagnostic/detection modalities
- [x] No [ ] Yes — Enable the weaponization of a biological agent or toxin
- [x] No [ ] Yes — Any other potentially harmful combination of experiments and agents

Plants

Seed stocks

Novel plant genotypes

Authentication

ChIP-seq

Data deposition

- [ ] Confirm that both raw and final processed data have been deposited in a public database such as GEO.
- [ ] Confirm that you have deposited or provided access to graph files (e.g. BED files) for the called peaks.

Data access links
May remain private before publication.

Files in database submission

Genome browser session
(e.g. UCSC)

Methodology

Replicates

Sequencing depth

Antibodies

Peak calling parameters

Data quality

Software

Flow Cytometry

Plots

Confirm that:

☐ The axis labels state the marker and fluorochrome used (e.g. CD4-FITC).

☐ The axis scales are clearly visible. Include numbers along axes only for bottom left plot of group (a 'group' is an analysis of identical markers).

☐ All plots are contour plots with outliers or pseudocolor plots.

☐ A numerical value for number of cells or percentage (with statistics) is provided.

Methodology

Sample preparation

Instrument

Software

Cell population abundance

Gating strategy

☐ Tick this box to confirm that a figure exemplifying the gating strategy is provided in the Supplementary Information.

Magnetic resonance imaging

Experimental design

Design type

Design specifications

Behavioral performance measures

Imaging type(s)

Field strength

Sequence & imaging parameters

Area of acquisition

Diffusion MRI ☐ Used ☐ Not used

Preprocessing

Preprocessing software

Normalization

Normalization template

Noise and artifact removal

Volume censoring

Statistical modeling & inference

Model type and settings

Effect(s) tested

Specify type of analysis: ☐ Whole brain ☐ ROI-based ☐ Both

Statistic type for inference

(See Eklund et al. 2016)

Correction

Models & analysis

n/a	Involved in the study
☐	☐ Functional and/or effective connectivity
☐	☐ Graph analysis
☐	☐ Multivariate modeling or predictive analysis

Functional and/or effective connectivity

Graph analysis

Multivariate modeling and predictive analysis

This checklist template is licensed under a Creative Commons Attribution 4.0 International License, which permits use, sharing, adaptation, distribution and reproduction in any medium or format, as long as you give appropriate credit to the original author(s) and the source, provide a link to the Creative Commons license, and indicate if changes were made. The images or other third party material in this article are included in the article's Creative Commons license, unless indicated otherwise in a credit line to the material. If material is not included in the article's Creative Commons license and your intended use is not permitted by statutory regulation or exceeds the permitted use, you will need to obtain permission directly from the copyright holder. To view a copy of this license, visit http://creativecommons.org/licenses/by/4.0/
