# Supplementary material for: Cep57 organizes pericentriolar material through dual architectural modules and prevents human mosaic variegated aneuploidy
Source: Nat Commun. 2026 Sep 30;17:11262. doi: 10.1038/s41467-026-77795-4 (PMC13720213; doi:10.1038/s41467-026-77795-4)
Supplement: Supplementary file 4 [file 41467_2026_77795_MOESM4_ESM.pdf]

Description of Additional Supplementary Files

Supplementary Movie 1

Description: 3D-MINFLUX images showing endogenous Cep57 localized around the centriole in U2OS cells. Supplementary Movie 2. 3D-MINFLUX images showing merged endogenous Cep57 and acetylated tubulin localizations acquired from six centrioles each in U2OS cells.

Supplementary Movie 2

Description: 3D-MINFLUX images showing merged endogenous Cep57 and acetylated tubulin localizations acquired from six centrioles each in U2OS cells.

Supplementary Movie 3

Description: 3D-MINFLUX images showing stably expressed Cep57 WT and the oligomerization-defective PP mutant localized around the centriole in U2OS cells depleted of endogenous Cep57 by RNAi.
